# Nrf2 as a therapeutic target of ginseng: A comprehensive review from preclinical evidence to clinical applications

**DOI:** 10.1016/j.jgr.2026.101079

**Published:** 2026-06-24

**Authors:** Bella Rhea Lavifa Sanjaya, Lilik Duwi Wahyudia, Min Kyung Cho

**Affiliations:** Department of Pharmacology, College of Oriental Medicine, Dongguk University, Gyeongju, 38066, South Korea

**Keywords:** Apoptosis, Ferroptosis, Ginseng, Inflammation, Nuclear factor erythroid 2-like 2, Oxidative stress, Pyroptosis

## Abstract

Ginseng is a traditional herb that has been widely used for centuries to treat various diseases. A key mechanism of ginseng is the activation of nuclear factor erythroid 2-like 2 (Nrf2), a master regulator of antioxidant gene expressions. Activation of Nrf2 helps maintain redox homeostasis, which is crucial for chronic disease management. This review aims to summarize the pleiotropic role of Nrf2 in mediating the biological effects of bioactive compounds, extracts, and formulations of ginseng. It provides a comprehensive overview of current research on the medicinal applications of ginseng for treating various diseases and highlights the mechanisms involved in Nrf2 regulation. The literature published from 2018 to 2025 were reviewed using a PubMed search to assess recent studies on the therapeutic applications of ginseng mediated by Nrf2. Ginseng activates the Nrf2 signaling pathway and modulates its upstream signaling, including Kelch-like ECH-associated protein 1 (Keap1), p62, and glycogen synthase kinase-3β (GSK-3β). It exhibits anti-oxidative stress, anti-inflammation, anti-apoptosis, anti-ferroptosis, and anti-pyroptosis properties in both *in vitro* and *in vivo* disease models. The causal relationship between Nrf2 regulation and the protective effects of ginseng was demonstrated in Nrf2/its upstream regulators knockout and knockdown models. Clinical studies including those on Korean red ginseng (KRG) and Shenmai Injection (SMI) have validated the safety and efficacy of ginseng in improving several pathological conditions. The pleiotropic pharmacological effects of ginseng are attributed to Nrf2 activation and stabilization. These findings offer significant potential for the therapeutic applications of ginseng.

## Highlights

• Ginseng bioactive compounds, extracts, and formulations induce Nrf2 signaling *in vitro* and *in vivo* disease models.
• Nrf2 activation mediates ginseng's anti-oxidant, anti-inflammatory, anti-apoptotic, anti-ferroptotic, and anti-pyroptotic effects.
• Ginseng-induced Nrf2 activation and stabilization support its potential for future therapeutic development.

## Graphical abstract

**Figure:**
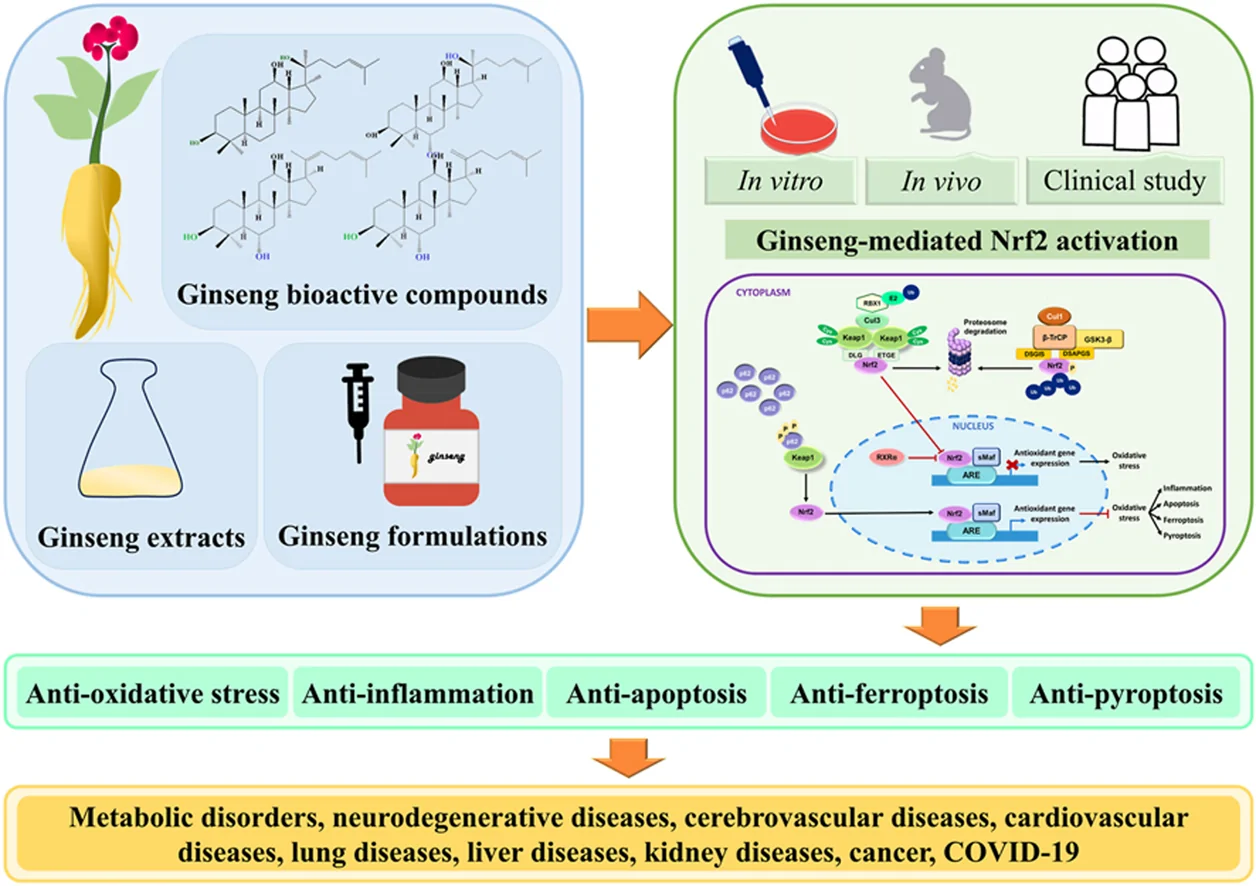

## Abbreviations:

β-TrCP: Beta-transducin repeat-containing protein
ACSL4: Acyl-CoA synthetase long-chain family member 4
ARE: Antioxidant response element
ASC: Apoptosis-associated speck-like protein containing a CARD
BACH1: BTB and CNC homology 1
Bak: Bcl-2-associated protein k
Bax: Bcl-2-associated protein x
Bcl-2: B-cell lymphoma 2
CNC: Cap ’n’ collar
COX-2: Cyclooxygenase-2
CUL3: Cullin 3
DISC: Death-inducing signaling complex
FADD: Fas-associated death domain
FTH: Ferritin heavy chain 1
GCLC: Glutamate-cysteine ligase catalytic subunit
GCLM: Glutamate-cysteine ligase modifier subunit
GLP151: Ginseng major latex-like protein151
GPX4: Glutathione peroxidase 4
GSDMD: Gasdermin D
GSK-3β: Glycogen synthase kinase-3β
GST: Glutathione S-transferase
HO-1: Heme oxygenase-1
IKK: IκB kinase
IL: Interleukin
iNOS: Inducible nitric oxide synthase
Keap1: Kelch-like ECH-associated protein 1
KRG: Korean red ginseng
LDH: Lactate dehydrogenase
LPA: Lysophosphatidic acid
MDA: Malondialdehyde
Neh: Nrf2-ECH homology
NF-κB: Nuclear factor kappa B
NLRP3: Nucleotide-binding oligomerization domain (NOD)-like receptor containing pyrin domain 3
Nrf2: Nuclear factor erythroid 2-like 2
NQO1: NAD(P)H quinone dehydrogenase 1
PPD: Protopanaxadiol
PPT: Protopanaxatriol
Rbx1: Ring-box 1
ROS: Reactive oxygen species
RXRα: Retinoid X receptor alpha
Skp1: S-phase kinase-associated protein 1
SLC7A11: Solute carrier family 7 member 11
sMafs: Small musculoaponeurotic fibrosarcoma
SMI: Shenmai injection
SOD: Superoxide dismutase
SQSTM1: Sequestosome 1
TFR: Transferrin receptor
TNF-α: Tumor necrosis factor alpha
TRADD: Tumor necrosis factor 1 receptor-associated death domain
TRAIL: Tumor necrosis factor-related apoptosis-inducing ligand

## 1 Introduction

Ginseng, belonging to the genus *Panax*, is a highly valued traditional herbal remedy used in functional foods and medicines worldwide, particularly in Korea, China, Japan, Russia, and the United States [1]. The medical constituents of various *Panax* species, including *P. ginseng*, *P. zingiberensis P. bipinnatifidus, P. quinquefolius, P. stipuleanatus, P. trifolius, P. vietnamensis, P. notoginseng*, *P. japonicus*, *P. wangianus,* and *P. pseudoginseng,* are currently being utilized in drug development across 35 countries [1]. The term “Panax” is derived from “panacea”, highlighting its historical use as a cure for various diseases and a nutritional supplement for physical strength, resistance, and longevity [2]. Ginseng has been extensively studied to treat diseases, including metabolic disorders, neurodegenerative, cerebrovascular, cardiovascular, and liver diseases, cancer, and coronavirus disease-2019 (COVID-19) [[3], [4], [5], [6], [7], [8]]. Ginseng's bioactive compounds, extracts, and formulations have been used as medical herbs or natural health products in various therapeutic applications.

The antioxidant effect of ginseng has been reported to be one of the main mechanisms for alleviating symptoms of various diseases, including diabetes, neurological, liver, kidney, vascular, and other age-related diseases [8]. Ginseng extracts, such as *P. ginseng* roots, have been shown to be effective in relieving chronic fatigue. Korean red ginseng (KRG) extract has been reported to have antipruritic, hepatoprotective, and anticancer effects [[9], [10], [11], [12]]. Shenmai injection (SMI) or Shenmaisan composed of red ginseng and Radix Ophioppogonis, is a traditional Chinese formula applied in the treatment of coronary heart disease and cancer [13]. *In vitro* and *in vivo* preclinical studies reveal the antioxidant properties of ginseng bioactive compounds, extracts, and formulations [[14], [15], [16], [17]]. Ginsenosides, the main bioactive compounds of ginseng maintain homeostasis via antioxidant activity by regulating the inflammation and cell death pathways [2,18].

Accumulation of reactive oxygen species (ROS) triggers oxidative stress, targeting cellular macromolecules and promoting disease progression. Under oxidative stress, nuclear factor erythroid 2-like 2 (Nrf2), a redox-sensitive transcription factor, is activated to control redox balance [19]. Nrf2 binds to the promoters of antioxidant genes, enhancing cellular defense against oxidative insults [20]. Nrf2 performs pleiotropic roles such as metabolic regulation, inflammation, and programmed cell death, proteostasis, mitochondrial physiology, and immune responses [20,21]. The activity of Nrf2 is inhibited by Kelch-like ECH-associated protein 1 (Keap1), an adaptor of a redox-sensitive E3 ubiquitin ligase substrate [22]. A complex regulatory system including beta-transducin repeat-containing protein (β-TrCP), glycogen synthase kinase-3β (GSK3β), small musculoaponeurotic fibrosarcoma (sMaf), and BTB and CNC homology 1 (BACH1) tightly regulates Nrf2 activity [23]. Nrf2 dysregulation has been linked to diverse diseases associated with oxidative stress [24].

Regular consumption of ginseng has been proven to increase the antioxidant defense [25]. Ginseng increases antioxidant activities and biomarkers, including catalase (CAT), glutamate-cysteine ligase catalytic subunit (GCLC), glutathione peroxidase (GPx), glutathione reductase (GR), glutathione s-transferases (GST), heme-oxygenase 1 (HO-1), superoxide dismutase (SOD), thioredoxin-1 (Trx1) via Nrf2 activation [26]. The use of ginseng shows promise in activating Nrf2 to suppress oxidative stress-related chronic diseases. In this review, we summarize the role of bioactive compounds of ginseng, its extracts, and formulations in regulating Nrf2, thus offering updated literature on the preclinical and clinical applications of ginseng as a therapeutic intervention.

## 2 Classification of ginseng compounds

**Fig. 1:**
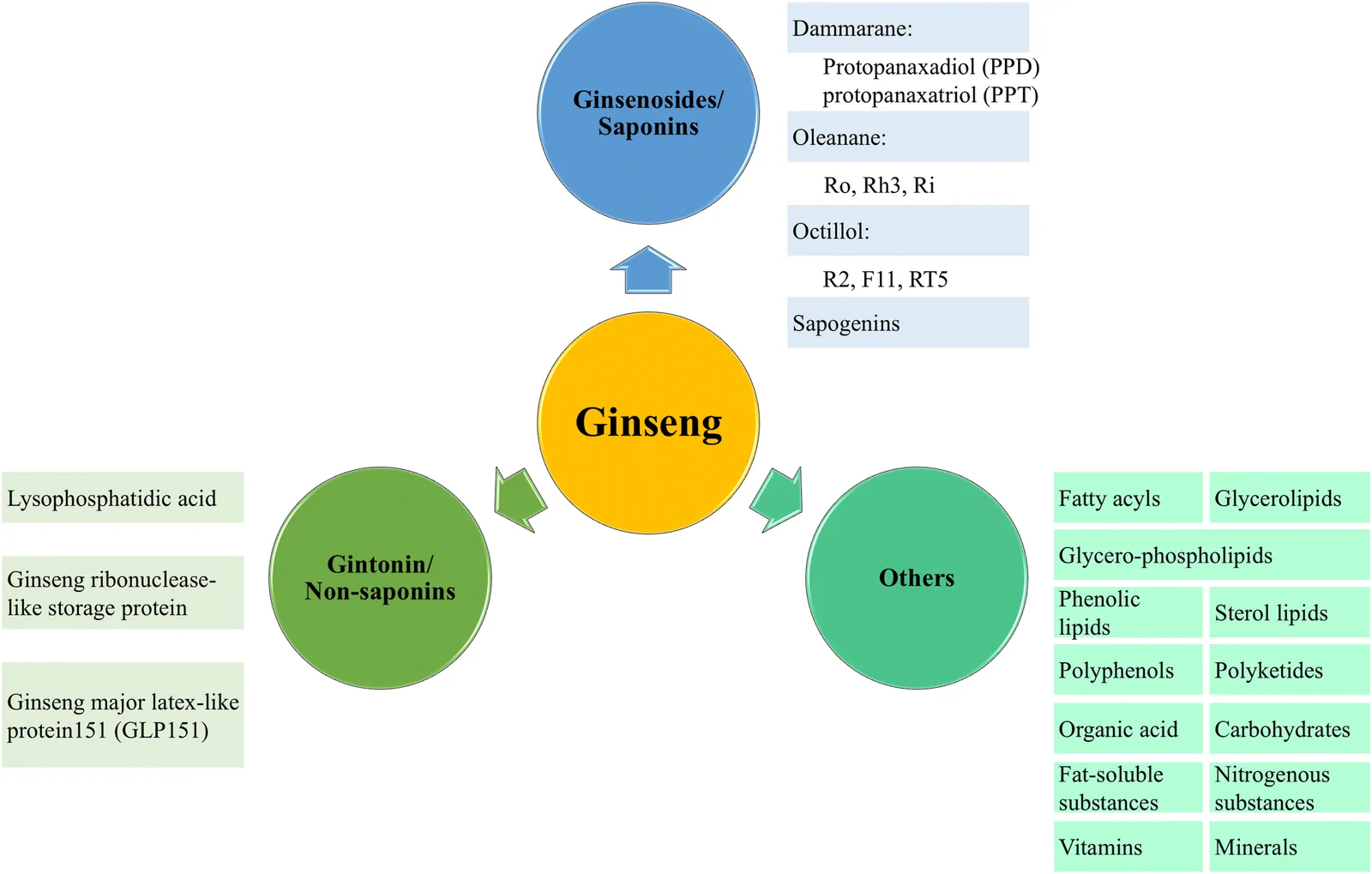
Bioactive compounds in ginseng.

Ginseng is rich in bioactive compounds categorized as ginsenosides (saponins), gintonin (non-saponins), and others (Fig. 1). Ginseng contains at least 3-6% ginsenosides, which are the main active ingredients of ginseng and are known to improve organ functions [27]. Ginsenosides are classified into a diverse group of compounds, including dammarane, oleanane, octillol, and sapogenins [28]. Dammaranes have four-ring, steroid-like structures with free or monomeric, dimeric, and trimeric sugar conjugates, which can alter nuclear-receptor binding and influence hormone-related diseases [29]. Dammarane derivatives are classified into two categories according to the number and position of hydroxyl groups: protopanaxadiol (PPD) (carbon-3 and -20) and protopanaxatriol (PPT) (carbon-3, -6, and -20) (Supplementary Fig. 1) [30]. Dammarane derivatives generally modulate adaptogenic action [30]. The fibrous roots of American ginseng contain ∼2 times more ginsenoside than the main root [31]. Heat processing or fermented biotransformation results in changes in the ginsenoside content [32,33].

Ginseng also contains non-saponin/gintonin which includes lysophosphatidic acid (LPA), ginseng ribonuclease-like storage protein, and ginseng major latex-like protein151 (GLP151) [1,27]. Gintonin, a glycolipoprotein complex, contains lipid-derived components, LPAs, lysophosphatidylinositols, and linoleic acid, which are believed to act as ligands of the LPA receptors [34]. Gintonin elicits intracellular Ca^2+^ as a second messenger, which triggers the release of neurotransmitters such as glutamate, acetylcholine, and dopamine [35]. It also contains other essential components such as fatty acyls, glycerolipids, glycerophospholipids, phenolic lipids, sterol lipids, polyphenols, polyketides, organic acid, carbohydrates, fat-soluble substances, nitrogen-containing substances, vitamins, and minerals [1].

## 3 The regulation of Nrf2

In living organisms, antioxidant systems neutralize excess ROS, thus maintaining cellular functions and homeostasis [20]. Uncontrolled ROS causes inflammation and various types of cell death that contribute to pathological alterations [36]. To respond against oxidative stress, Nrf2 acts as a master transcription factor that regulates antioxidant genes [37]. Antioxidant genes are categorized into three types of antioxidant defense: antioxidant enzymes, antioxidant substances, and repair enzymes [38]. In the first line of defense, antioxidant enzymes suppress the formation of free radicals. Antioxidant substances like glutathione (GSH), ascorbic acid, and uric acid act as scavengers, neutralizing free radicals and rendering them harmless. The third line of defense involves repair enzymes and proteolytic enzymes, which recognize and repair the damage caused by free radicals [39]. Thus, antioxidants counterbalance oxidants in the human body.

**Fig. 2:**
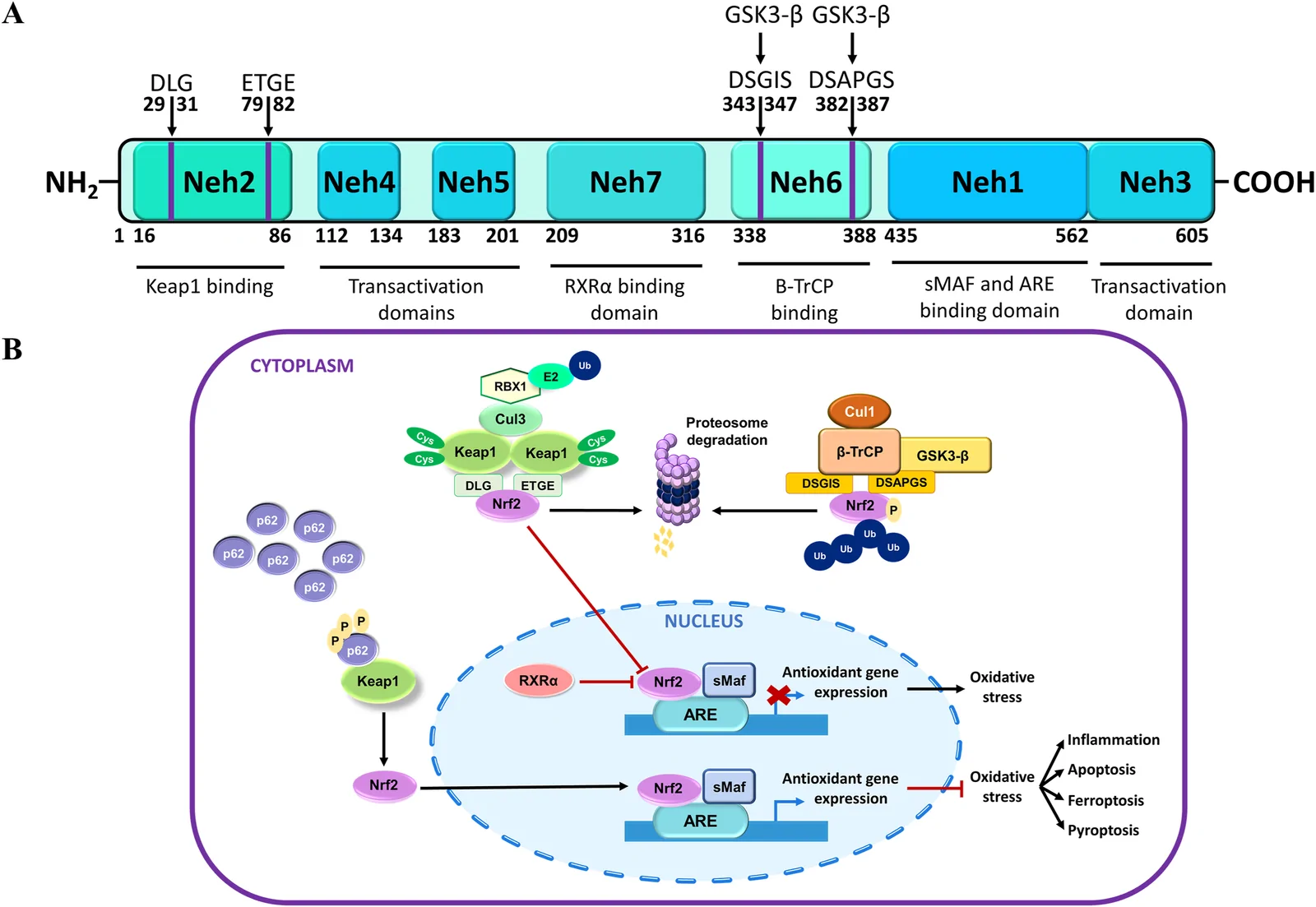
Regulatory pathways of Nrf2 signaling. (A) Functional domain of Nrf2 and (B) Molecular mechanism of Nrf2 activation and response.

Nrf2 is a family of Cap ’n’ collar (CNC) transcription factors consisting of seven Nrf2-ECH homology domains (Neh1-7) (Fig. 2A) [36]. The stability and activity of Nrf2 are controlled by its distinct functional domains. The Neh1 CNC basic-region leucine zipper (bZIP) domain is responsible for DNA binding and heterodimerization with sMaf. The C-terminal Neh3 domain is required for transactivation, while the Neh4 and Neh5 domains contribute to transcriptional activation through interactions with coactivator. DNA binding to the antioxidant response element (ARE) is mediated by the Neh 1 CNC bZIP domain. At the N-terminus of Nrf2, the Neh2 domain regulates its stability by interaction with Keap1, a substrate adaptor of the Cullin 3 (Cul3)-based E3 ligase complex [40]. Retinoid X receptor alpha (RXRα) inhibits the Nrf2-ARE signaling pathway by direct interaction with the Neh7 domain of Nrf2 [22]. GSK3β phosphorylates the Neh6 domain, promoting its interaction with β-TrCP within the Cul1 E3 ligase complex, resulting in ubiquitination and degradation of Nrf2 [22].

Keap1 is the first reported regulator of Nrf2 repression (Fig. 2B). Under normal conditions, Keap1 binds to Nrf2 in the cytoplasm and facilitates its ubiquitination through the Keap1-Cul3-Ring-box 1 (Rbx1) E3 ubiquitin ligase complex [41,42]. Within the Neh2 domain of Nrf2, the DLG and ETGE motifs are required for binding to Keap1 and ubiquitination of Nrf2 [43]. The ubiquitinated Nrf2 is then targeted for degradation by the 26S proteasome, resulting in low levels of Nrf2 in the cytoplasm. This allows Nrf2 to maintain cellular homeostasis without unnecessarily activating target genes. ROS or electrophilic molecules modify key cysteine residues on Keap1 [43]. Oxidation of cysteine residues by ROS induces a conformational change and releases Nrf2 from the Keap1-Nrf2 complex [21]. Then, this leads to Nrf2 stabilization and nuclear translocation, and consequently activates ARE-driven target genes, including those involved in detoxification, antioxidant defense, and cell survival [44].

p62/sequestosome 1 (SQSTM1) is known as a multifunctional autophagy adaptor that facilitates the degradation of aggregated or misfolded proteins [45]. p62 also participates in the Keap1-Nrf2 system by competitively binding to Keap1 via its Keap1-interacting region (KIR), thereby disrupting the Keap1-Nrf2 interaction (Fig. 2B). The phosphorylation and accumulation of p62 markedly increase its binding affinity for Keap1, facilitating the stabilization and hyperactivation of Nrf2, thereby promoting its transcriptional activity during high oxidative stress or damage [46]. Additionally, Nrf2 can induce p62 expression via ARE in the promoter of p62 creating a positive feedback loop [47].

GSK-3β/β-TrCP controls Nrf2 stability in a Keap1-independent mechanism (Fig. 2B) [48]. Phosphorylation of the Neh6 domain in Nrf2 by GSK-3β increases Nrf2 ubiquitination by β-TrCP with S-phase kinase-associated protein 1 (Skp1)-Cul1-Rbx1 ubiquitin ligase complex [49]. GSK-3β inhibitors stabilizes Nrf2, while overexpression of β-TrCP enhances its ubiquitination and degradation. Nrf2 is also controlled by the binding of RXRα in the Neh7 domain of Nrf2, which hinders ARE-driven gene expression [50]. The binding of RXRα to the Neh7 domain blocks the interactions of coactivator CREB-binding protein (CBP) to the Neh4 and Neh5 domains of Nrf2, which mediates the transactivation of Nrf2 target genes [51]. Mutation of RXRα increased ARE-driven gene expression, whereas its overexpression reversed the enhancement, supporting the antagonistic role of RXRα on Nrf2 function [50]. 3-Hydroxy-3-methylglutaryl-CoA (HMG-CoA) reductase degradation protein 1 (HRD1), a novel E3 ubiquitin ligase, enhances Nrf2 ubiquitination and subsequent degradation by directly interacting with its Neh4 and Neh5 domains [52]. Other signaling proteins such as AMP-activated protein kinase and casein kinase 2 modulate the stability and localization of Nrf2, leading to transactivation of antioxidant genes [53].

## 4 Functional roles of Nrf2

The canonical role of Nrf2 is the regulation of antioxidant responses and the protection of cells from oxidative stress and electrophilic damage. Antioxidant enzymes including SOD, GPxs, HO-1, CAT, and thioredoxin reductase (TrxR), detoxification enzymes such as glutathione s-transferase alpha 1 (GSTA1) and NAD(P)H quinone dehydrogenase 1 (NQO1), and glutathione synthesis enzymes such as GCLC and glutamate-cysteine ligase modifier subunit (GCLM) help to mitigate oxidative damage and maintain cellular homeostasis [26].

Nrf2 also plays a role in regulating inflammation, under conditions in which ROS production surpasses antioxidant defense capacity [54]. The inflammatory response is marked by Nrf2 deficiency, supporting the interplay between Nrf2 and nuclear factor-κB (NF-κB) [55]. The NF-κB family of transcription factor includes p50, and p52, RelA (p65), RelB, c-rel, controlling inflammation, immune response, and cell survival [56]. NF-κB regulates genes involved in inflammatory cytokine production, immune cell activation, and apoptosis [55]. NF-κB remains inactive due to its association with IκB (the inhibitor of κB) under resting conditions, and external stimulation initiates the phosphorylation of the IκB kinase (IKK) complex. The complex, composed of two catalytically active kinases, IKKα and IKKβ, and the regulatory subunit IKKγ (NF-κB essential modulator (NEMO)). It induces IκB phosphorylation, ubiquitination, and proteasomal proteolysis of IκB, resulting in the release of NF-κB, nuclear translocation of NF-κB and transcriptional activation of the genes [57]. The overexpression or chronic activation of NF-κB has been implicated in both the initiation and progression of multiple diseases such as liver failure, diabetes, and cancer [58]. Activation of Nrf2 is protective against chronic inflammation because it can reduce the underlying oxidative stress that exacerbates the inflammatory response. Negative regulation of Nrf2 by p65 has been attributed to competition for the transcriptional coactivator CBP (CREB-binding protein)/p300 complex. Competition between Neh4 and Neh5 of Nrf2 and Ser^276^ of p65 for CBP/p300 binding affects the transcriptional regulation of oxidative stress and inflammatory responses [55]. In addition, p65 can promote histone deacetylase 3 (HDAC3) association with MafK, leading to dissociation of heterodimer formation with Nrf2 [59]. One of the Nrf2 target genes, HO-1 has been shown to interfere with NF-κB signaling by generating carbon monoxide [55]. β-TrCP, one of the upstream regulators of Nrf2, also leads to augmentation of NF-κB activity as well as to the inhibition of Nrf2 transcription [60]. This antagonistic crosstalk between Nrf2 and NF-κB through indirect interaction has been revealed to be important in developing therapeutic strategies for inflammation-related diseases.

Nrf2 plays a central role in regulating cell survival and death by modulating oxidative stress responses. Prolonged exposure to oxidative stress triggers various types of programmed cell death, including apoptosis, ferroptosis, and pyroptosis [61]. Apoptosis is a type of programmed cell death that serves as a defense mechanism against oxidative stress. It activates a series of caspases, including initiator caspases (caspase 8 and 9) and executioner caspases (caspase 3, 6, and 7), resulting in cell death [62]. Two major apoptotic pathways proceed through death receptors in the extrinsic route and mitochondria in the intrinsic route, both of which converge on a common execution pathway. The pro-apoptotic markers involved in the extrinsic pathway are tumor necrosis factor-related apoptosis-inducing ligand (TRAIL), death-inducing signaling complex (DISC), Fas ligand, tumor necrosis factor-alpha (TNF-α), tumor necrosis factor 1 receptor-associated death domain (TRADD), Fas-associated death domain (FADD), TNF-like WEAK inducer of apoptosis (TWEAK), and nerve growth factor, while those in the intrinsic pathway are B-cell lymphoma 2 (Bcl-2) family group II and III (Bim, Bmf, Bik, Bad, Bid, Bax, and Bak [63]. Nrf2 also transcriptionally induces the anti-apoptotic protein Bcl-2 family, followed by decreased Bax, cleaved caspase 3/7, and increased cell survival, suggesting the potential role of Nrf2 in controlling apoptosis [64]. However, conflicting studies suggest that Nrf2 may contribute to caspase-independent apoptosis under severe oxidative stress or infection, resulting in an imbalance between cell survival and death signals [64].

Cellular GSH depletion and iron-dependent accumulation of lipid peroxides are hallmarks of ferroptosis [61]. Iron-driven Fenton reactions induce ROS production and lipid peroxidation. Polyunsaturated fatty acids in cell membranes are often oxidized by enzymes like acyl-CoA synthetase long-chain family member 4 (ACSL4) which disrupt cell membrane integrity leading to cell death [65]. Nrf2 controls lipid metabolism by regulating ACSL4 and lysophosphatidylcholine acyltransferase 3 (LPCAT3), and maintains iron homeostasis by inducing ferritin heavy chain1 (FTH1), transferrin receptor (TFR) and ferroportin 1 (FPN1) [66,67]. Nrf2-mediated antioxidant genes including HO-1, glutathione peroxidase 4 (GPX4), and NQO1 suppress ferroptosis by reducing lipid peroxide [61,68]. Cystine/glutamate antiporter solute carrier family 7 member 11 (SLC7A11/xCT, which is required for GSH synthesis, plays a role in suppressing ferroptosis [69]. Under specific conditions, HO-1 is induced by hemin to promote ferroptosis by increasing free iron levels via heme degradation [70]. Ferroptosis contributes to the progression of numerous diseases, including neurodegenerative, cerebrovascular, cardiovascular, kidney, and liver diseases, among others [67]. Iron homeostasis, ROS balance, glutathione synthesis, and lipogenesis regulated by Nrf2 have been suggested as pathways to explore therapeutic interventions in various diseases.

Pyroptosis is characterized by inflammatory responses, which occur through canonical (caspase 1) and non-canonical (caspase 4/5/11) pathways, releasing inflammatory cytokines [71]. In the canonical pathway, cytosolic pattern recognition receptors, which are capable of recognizing pathogen-associated molecular patterns (PAMPs) and danger-associated molecular patterns (DAMPs) assemble with nucleotide-binding oligomerization domain (NOD)-like receptor pyrin domain containing protein 3 (NLRP3), pro-caspase-1 and apoptosis-associated speck-like protein containing a CARD (ASC) to form inflammasomes when stimulated by bacterial and infections [61]. This leads to the activation of caspase 1 and cleavage of the pore-forming protein gasdermin D (GSDMD) [72]. In the non-canonical pathway, endotoxins such as lipopolysaccharides activate caspases 4/5/11, triggering the cleavage of GSDMD, and activation of pannexin-1 [73]. Pro-inflammatory cytokines, including interleukin-1β (IL-1β) and IL-18 are produced during pyroptosis, playing a crucial role in the immune response to infection and cellular stress. Excessive or dysregulated pyroptosis contributes to various autoimmune, neurodegenerative, and cardiovascular diseases and cancer [61]. The possible mechanism in the crosstalk between Nrf2 and inflammasomes has been only partially understood [74]. Compounds activating Nrf2 inhibit NLRP3 inflammasome activation in a complex manner, including indirect p62 regulation and direct targeting of inflammasome components, which remains to be evaluated [74].

## 5 Literature screening process

**Fig. 3:**
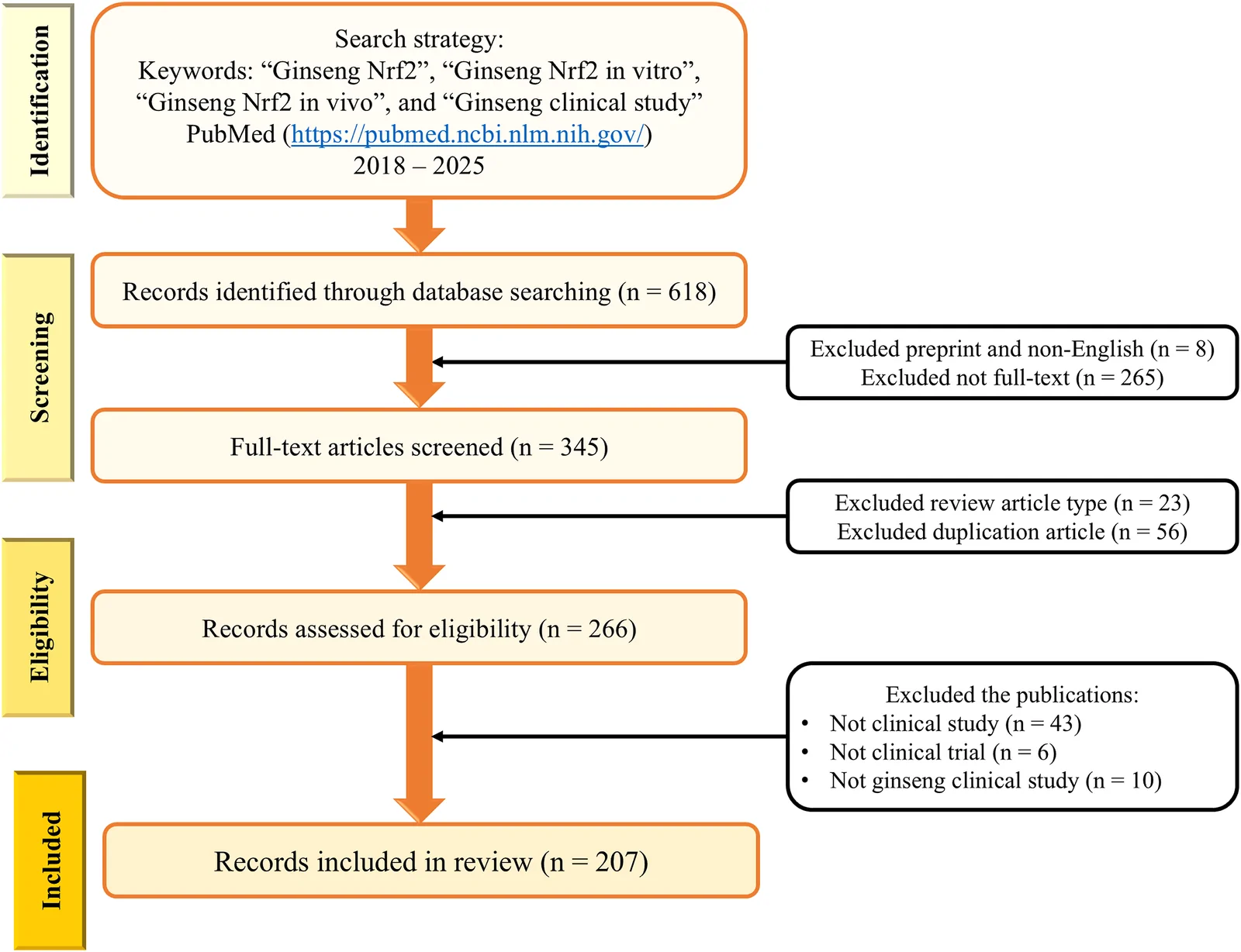
The flow-chart summary of the search process.

From 2018 to 2025, 618 recent studies on ginseng and Nrf2 were reviewed in PubMed (https://pubmed.ncbi.nlm.nih.gov/) with the keywords “ginseng nrf2”, “ginseng nrf2 *in vitro*”, “ginseng nrf2 *in vivo*” and “ginseng clinical study” (Fig. 3). Of these, 411 articles, including preprint, non-English, non-full-text, review articles, duplication articles, non-clinical study, non-clinical trial, and non-ginseng clinical study, were excluded from this study. Finally, 207 studies were included in this review. The findings of this review provide a comprehensive overview of ginseng-mediated Nrf2 activation, highlighting its therapeutic potential and providing valuable insights for drug development.

## 6 Ginseng-mediated Nrf2 activation

The bioactive compounds, extracts, and formulations of ginseng have been reported to activate Nrf2 signaling pathways in both *in vitro* and *in vivo* models through various pharmacological actions (Supplementary Table 1**,** Supplementary Table 2). Ginseng-mediated regulation of Nrf2 plays a role in anti-oxidative stress, anti-inflammation, anti-apoptosis, anti-ferroptosis, and anti-pyroptosis (Fig. 2B).

### 6.1 Anti-oxidative stress

Rb1, a glycosylated major PPD, has been shown to increase the Nrf2 signaling pathway leading to antioxidant gene induction with suppression of ROS in bronchial epithelial cells and pulmonary disease animal models [75]. Nrf2-mediated ROS inhibition by Rg3, Rh2, and F2 correlates with reduced levels of malondialdehyde (MDA) and lactate dehydrogenase (LDH), which are indicators of cellular damage [[76], [77], [78], [79], [80]]. Rg3, a metabolite of Rb1 with fewer sugar moieties, was found to attenuate oxidative stress with the activation of the Nrf2/HO1 pathway and Sirtuin 1 (SIRT1) modulation [81,82]. Compound K, derived from the hydrolysis of PPD by intestinal microflora, is believed to be a key bioactive metabolite because it is readily absorbed into plasma and exhibits improved pharmacological activities [12,32]. Compound K has been reported to cause the dissociation of Nrf2 from Keap1, leading to the activation of Nrf2-mediated anti-oxidative effects in Alzheimer's disease model and autoimmune hepatitis [83,84]. Antioxidant gene induction and anti-apoptotic effect by Nrf2-induced Bcl-2 family regulation and HO-1 induction by compound K have been reported in retinal pigment epithelial cells [85,86].

PPT, including Rg1 and Re, have been reported to induce Nrf2-mediated glutathione synthesis and antioxidant gene induction [87,88]. Rg1 increased GSK-3β inactivation leading to Nrf2 nuclear translocation [89]. Rg1, potentially showed anti-oxidative stress by enhancing the transactivation of Nrf2 to the ARE, thereby initiating the expression of downstream target genes against ischemia/reperfusion (I/R) neuronal injury, hepatotoxicity, and depression in *in vitro* studies [[90], [91], [92]]. Rg1-mediated Nrf2 induction showed improvement in metabolic disorders, cardiovascular diseases, and kidney and liver diseases [87,89,[92], [93], [94], [95], [96]]. F1, a type of deglycosylated PPT in ginseng is associated with cardioprotective effects through Keap1-mediated Nrf2 activation [80]. Notoginsenoside R1 increased Nrf2-mediated glutathione synthesis and antioxidant gene expression [89,97,98]. Rk1, a unique metabolite created by processing the ginseng plant, was confirmed as a new neuroprotective candidate for Parkinson's disease and Alzheimer's disease mediating its effects through Nrf2 activation [99,100].

Some researchers also investigated these biological activities using ginseng extract derived from roots, leaves, and flowers [16,[101], [102], [103], [104], [105]]. Ginseng root extract regulated the p62-Nrf2-Keap1 signaling pathway activating the antioxidant response *in vitro* and *in vivo* [101]. KRG exhibited a strong anti-oxidant effect via regulation of the Keap1/Nrf2 pathway in chronic inflammation and Parkinson's disease [106,107]. *P. notoginseng* saponin extracts facilitated the Nrf2-induced antioxidant system by downregulating Keap1 and inhibiting ROS [108].

Various species of ginseng are included in the composition of different formulations, including *P. ginseng* in SMI, *P. quinquefolium* in *Yixin-Fumain* granules, and *P. notoginseng* in *Fufang Lurong Juangu* capsules, and are observed to improve cardiomyopathy, sick sinus syndrome, and osteoporosis disease models [[109], [110], [111]]. These *in vivo* studies involving ginseng-containing formulations such as *P. notoginseng* in the *Guan Xin Dan Shen* formulation*, Hedansanqi Tiaozhi Tang* extract, *Tianhuang* formula, and Compound Danshen Dripping Pil, and *P. ginseng* in SMI and SPTC, a mixture of *Salvia officinalis*, *P. ginseng*, *Trigonella foenum-graeceum*, and *Cinnamomum zeylanicum* have played an important role in establishing the basis for clinical trials.

### 6.2 Anti-inflammatory effects

**Fig. 4:**
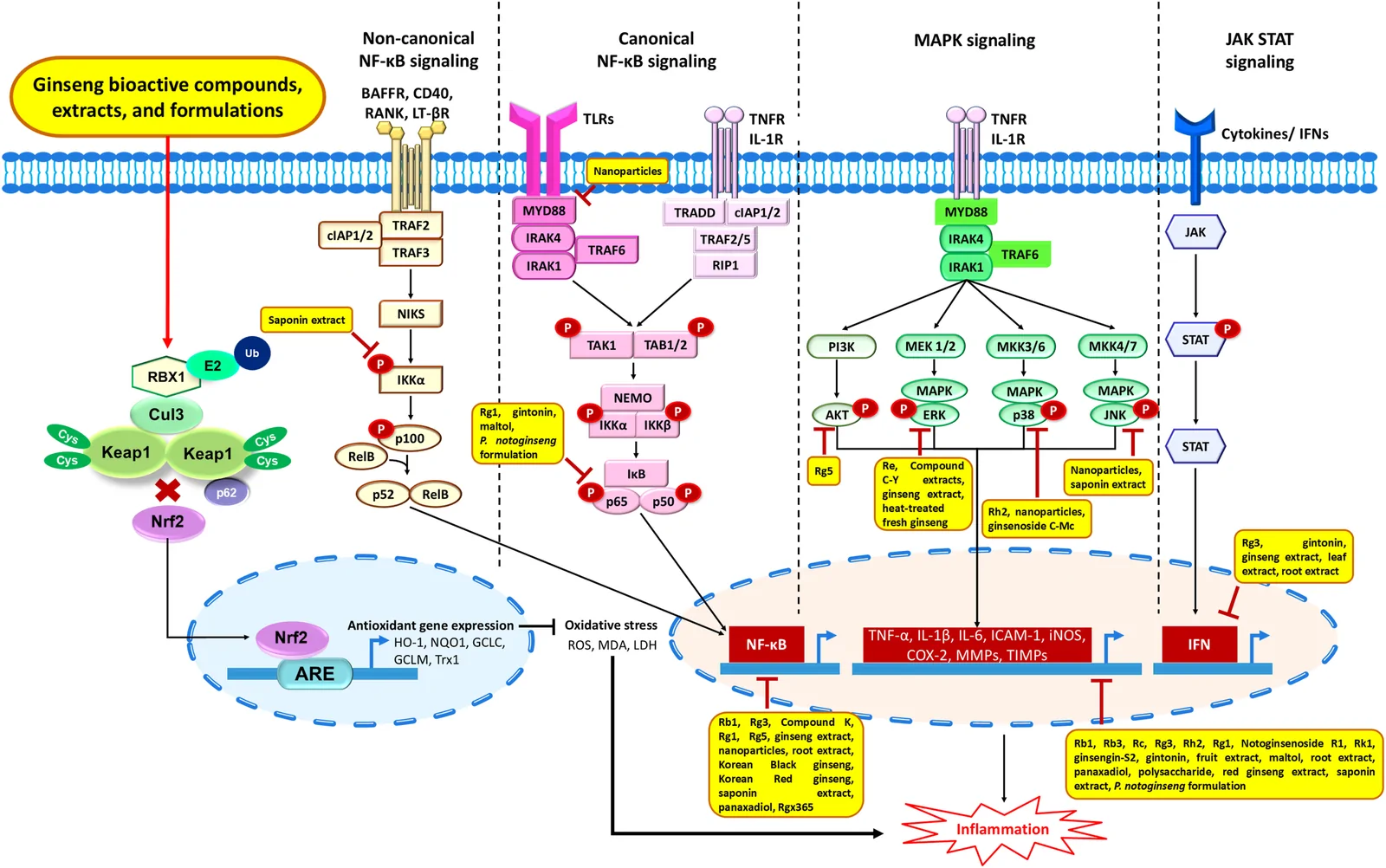
Regulations of inflammation signaling pathways by ginseng through Nrf2 activation.

An excessive amount of ROS can lead to the initiation and amplification of inflammatory responses, which are associated with numerous pathogenic conditions [54]. Bioactive compounds, extracts, and formulations of ginseng exhibit anti-inflammatory properties through Nrf2 signaling (Fig. 4). Ginseng-mediated Nrf2 activation inhibits IKK phosphorylation and NF-κB activation, subsequently leading to the suppression of inducible nitric oxide synthase (iNOS) and cyclooxygenase-2 (COX-2) [112]. Ginseng extracts have been proven to reduce pro-inflammatory mediators such as TNF-α and ILs [101,113]. Recent evidence indicates that Rg1, gintonin, and *P. ginseng* also suppresses pro-inflammatory mediators, along with the regulatory enhancement of Nrf2-target genes [[114], [115], [116], [117]]. Receptor tyrosine kinase (RTK)-linked phosphorylation of mitogen-activated protein kinase (MAPK) and protein kinase B (Akt) was inhibited by ginseng leading to suppression of proinflammatory cytokine production [[118], [119], [120]]. Cytokine-mediated signaling was inhibited by ginseng extracts including gintonin [34,121]. Saponins from *P. japonicus* and *P. notoginseng* exhibited inhibition of pro-inflammatory markers together with fibrosis [122,123]. Ginseng formulations mitigated inflammation via Nrf2 signaling and displayed improvements in ischemic stroke and pulmonary edema disease models [123,124].

### 6.3 Anti-apoptotic effects

**Fig. 5:**
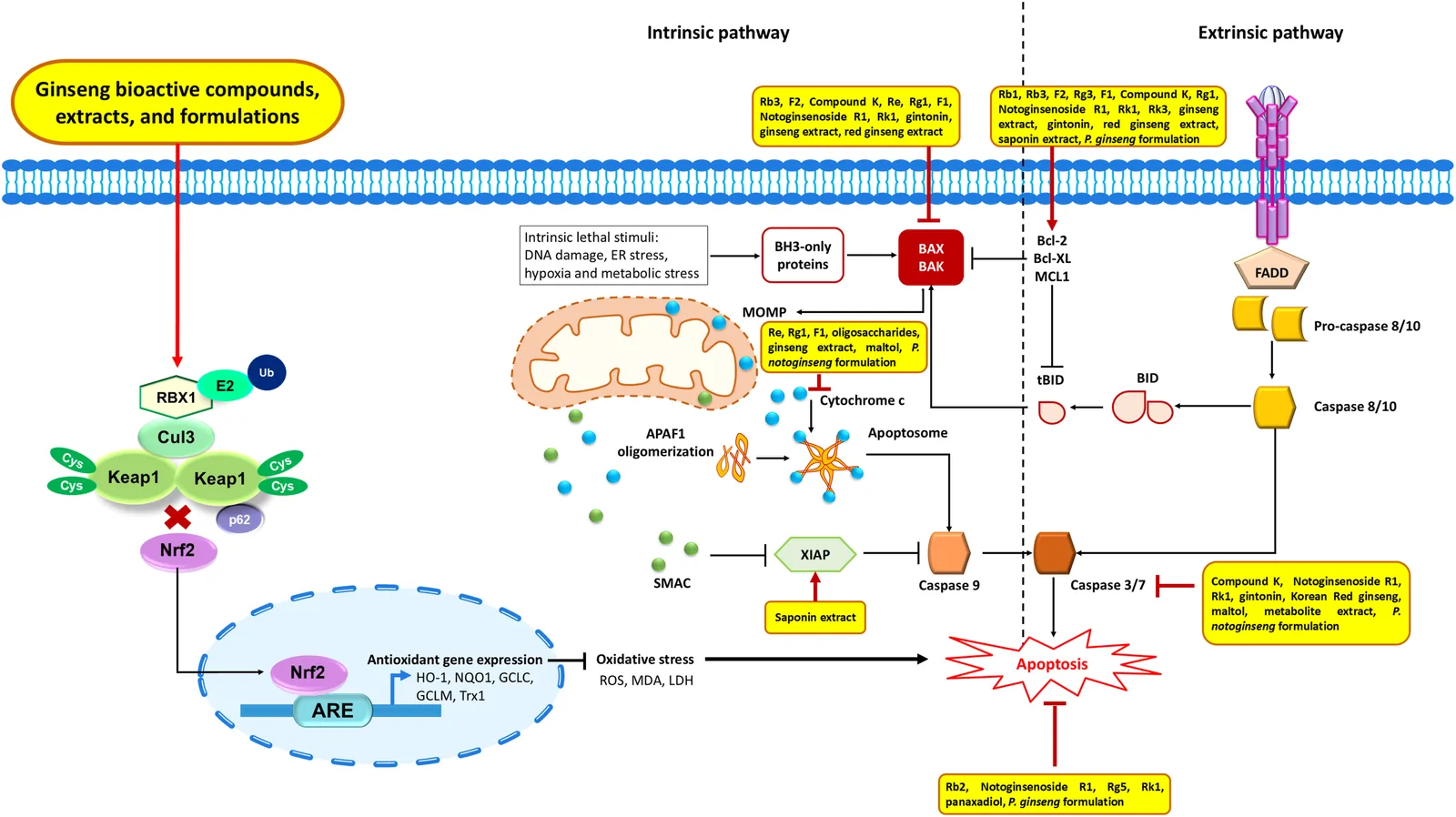
Modulation of apoptosis signaling pathways via ginseng-induced Nrf2.

Antioxidant activity has been shown to reduce ROS-dependent apoptosis [125]. Ginseng-mediated Nrf2 activation inhibits caspase cascades, thereby preventing oxidative stress-induced apoptosis (Fig. 5). Compound K (PPD) showed downregulation of pro-apoptotic markers and upregulation of anti-apoptotic markers, thereby improving neuronal injury in an *in vivo* Alzheimer's disease model [84]. Compound K exhibited an Nrf2-mediated anti-inflammatory effect in autoimmune hepatitis [83]. Ginsenoside Re (PPT) exhibits anti-apoptotic effects by modulating key components of both intrinsic and extrinsic pathways, including caspase 8, 3, and 9 [14,126]. Nrf2 and induction of the antioxidant genes under Rg1 (PPT) treatments reduced ROS and pro-apoptotic markers, resulting in lower levels of cytochrome C release as well as lower activation of initiator and executioner caspases [78,91]. Recent studies demonstrated that the upregulation of Nrf2-target genes by Notoginsenoside R1 linked to the suppression of pro-apoptotic markers [127,128]. Ginseng extracts exerted anti-apoptosis effects by upregulating anti-apoptotic markers, downregulating pro-apoptotic markers, and reducing the apoptotic rate [121,[129], [130], [131], [132]]. Gintonin inhibited oxidative stress and inflammation, followed by suppressing the apoptotic rate and improving motor dysfunction and behavioral alterations in an *in vivo* Parkinson's disease model [121]. The anti-apoptotic properties of ginseng formulations, mediated via Nrf2 activation, have demonstrated promising potential in reducing metabolic disorders and cardiovascular disease in animal models [109,133]. Most ginseng-derived bioactive compounds, extracts, and formulations have been reported to modulate the apoptosis pathway mostly by upregulating anti-apoptotic markers from the Bcl-2 family group I (Bcl-2 and Bcl-XL), while downregulating pro-apoptotic markers from the Bcl-2 family group II and III (Bak and Bax) (Fig. 5) [63].

### 6.4 Anti-ferroptotic effects

**Fig. 6:**
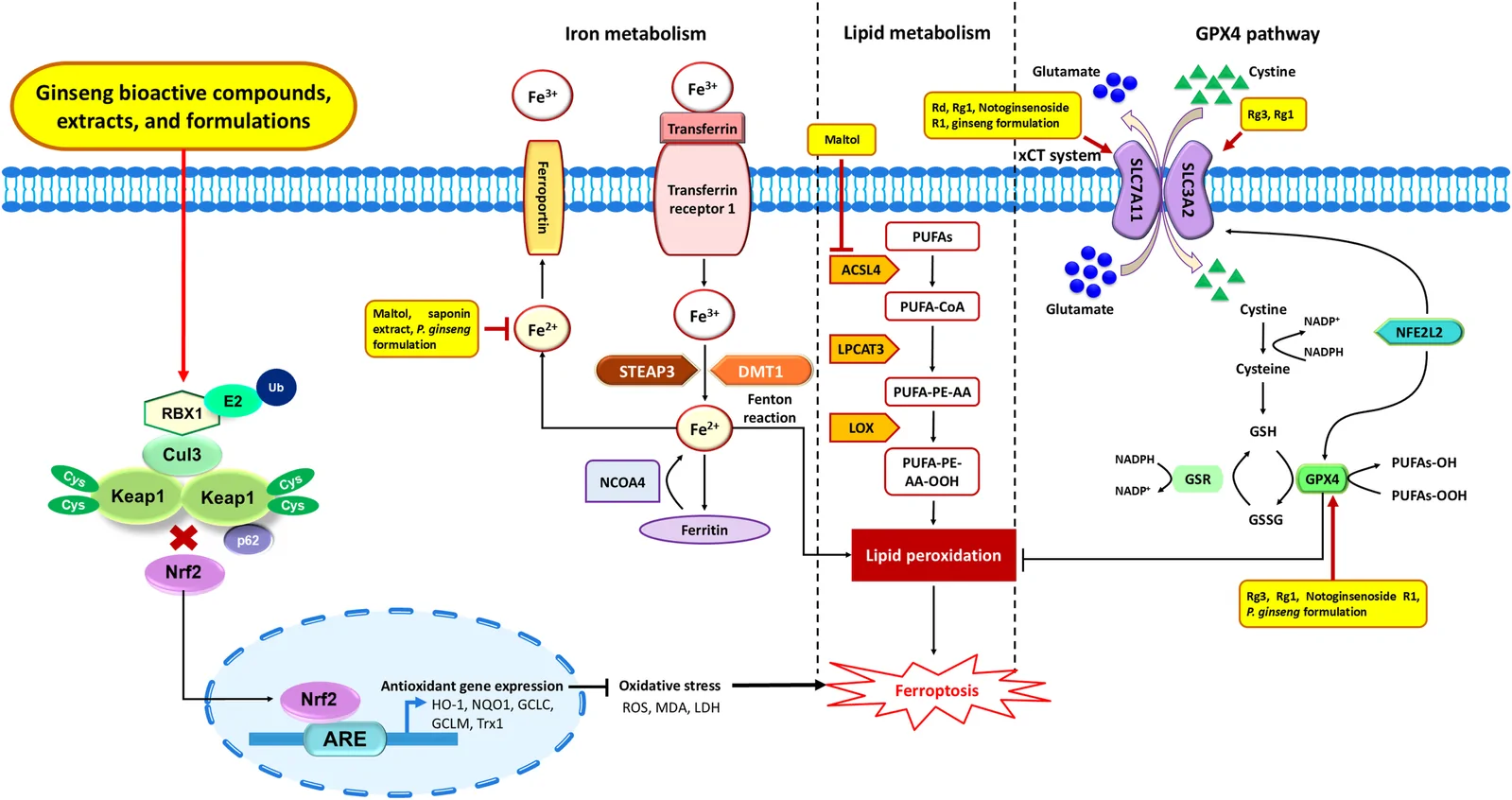
Modulatory effects of ginseng-activated Nrf2 on the ferroptosis signaling pathways.

In the Fenton reaction, hydrogen peroxide interacts with ferrous iron to produce highly reactive hydroxyl radicals, which then initiate the oxidation of polyunsaturated fatty acids leading to the formation of lipid peroxide [65]. Ginseng exhibits notable protective effects against ferroptosis which is driven by iron-dependent lipid peroxidation (Fig. 6). This anti-ferroptotic activity is mainly linked to the modulation of oxidative stress pathways, particularly through the activation of Nrf2. Activation of Nrf2 leads to the increased expression of key antioxidant and ferroptosis-suppressing genes such as GPX4 and components of the GSH system [134]. The bioactive compounds of ginseng including PPD (Rb1, Rd, and Rg3) and PPT (Rg1 and notoginsenoside R1) have been shown to regulate Nrf2 activation, which in turn upregulates key anti-ferroptosis markers including FTH, GSH, GPX4, and SLC7A11/xCT while downregulating ferroptosis-related proteins such as TFR and ACSL4, enabling it to neutralize lipid peroxide [94,[135], [136], [137]]. Current investigations indicate that activation of the Nrf2/p62 pathway by Rg5 leads to the upregulation of anti-ferroptosis markers [138]. Studies have shown that ginseng extracts enhance Nrf2 activity, which reduces oxidative stress and pro-ferroptosis markers [139,140]. Maltol, which is generated during the heat processing of red ginseng, has been shown to significantly mitigate ferroptosis [139]. Ginseng formulations have also been shown to have anti-ferroptotic effects contributing to the improvement in various disease models, including cardiovascular disease and ulcerative colitis [68,141].

### 6.5 Anti-pyroptotic effects

**Fig. 7:**
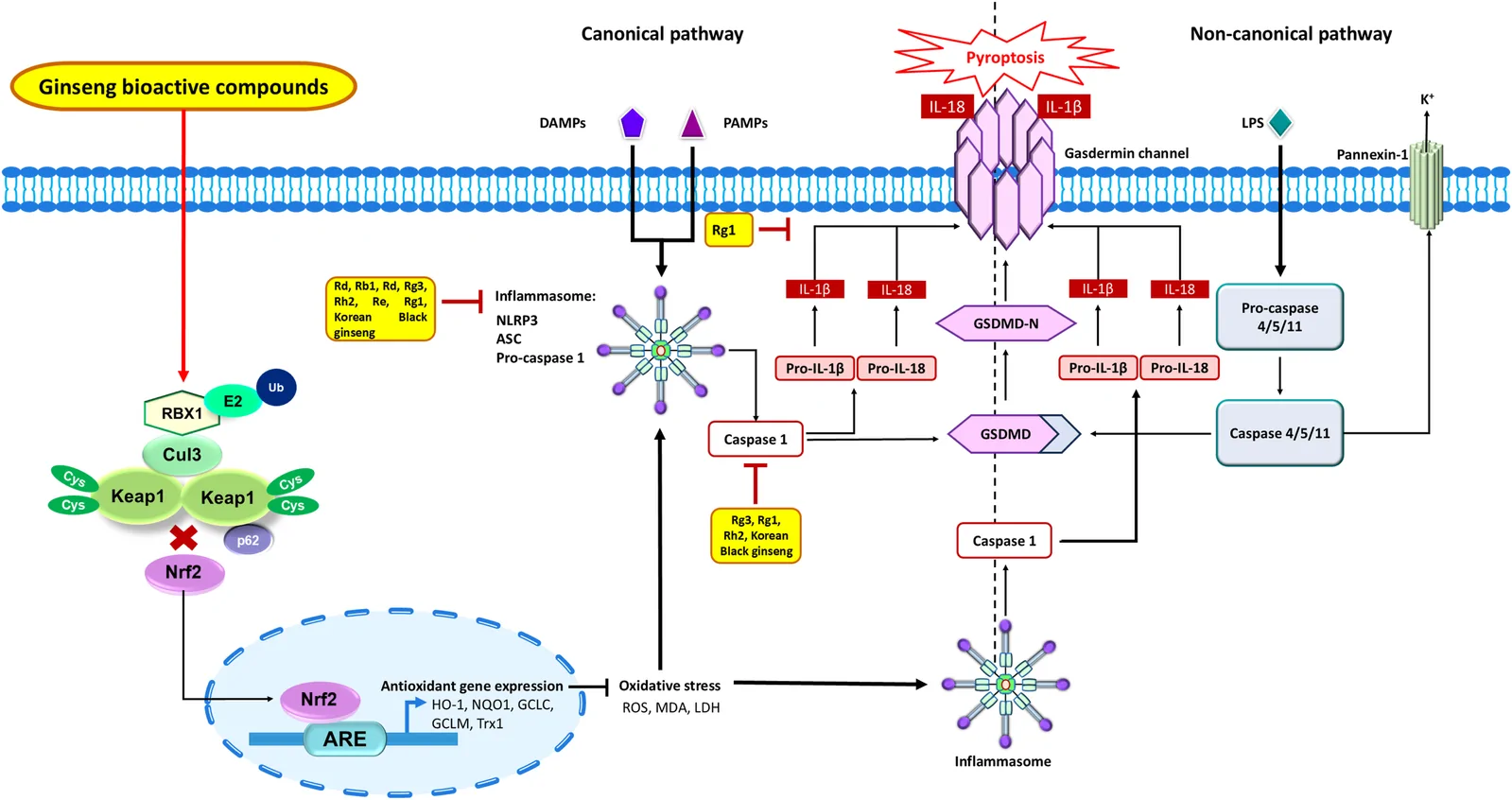
Role of ginseng-triggered Nrf2 in regulating pyroptosis signaling pathways.

Ginseng regulates the Nrf2 pathway, which plays a crucial role in modulating pyroptosis, a type of programmed cell death associated by inflammatory responses (Fig. 7). Ginsenosides have been shown to downregulate Keap1, thereby enhancing Nrf2 activation. This activation subsequently suppresses oxidative stress and inhibits NLRP3 inflammasome formation [95,142,143]. The inflammasome of multimeric complexes (NLRP3, ASC, and pro-caspase 1) has been reported to activate the gasdermin-mediated release of IL-1β and IL-18, leading to large pore formation and pyroptosis [74]. Ginsenosides of PPD (Rd, Rg3, and Rh2), PPT (Re and Rg1), and Korean black ginseng have been shown to upregulate Nrf2 target genes, thereby reducing caspase-1-dependent canonical pyroptotic markers [77,81,94,144]. Ginseng-mediated Nrf2 inductions contribute to improvements in various disease models, including metabolic disorders, cerebrovascular diseases, and liver diseases, partly through the modulation of pyroptosis [87,93,142].

## 7 Functional studies of ginseng on Nrf2

Ginseng-mediated Nrf2 induction is recognized for its protective effects in both *in vitro* and *in vivo* disease models (Supplementary Table 1, Supplementary Table 2). Cells or animals lacking Nrf2 through knockdown or knockout are considered more vulnerable to redox imbalances and external stressors associated with various diseases [145]. Nrf2-inducing activities by ginseng-containing bioactive compounds, extracts, and formulations were partially reduced or inhibited in human, mouse, and rat cells in Nrf2 knockdown cellular and animal models (Table 1). Bioactive compounds and formulations of ginseng significantly attenuated the Nrf2 target gene expressions in Nrf2 knockdown models, resulting in dysregulation of redox metabolism and inflammation [91,147,151,154,157,160,161]. Rb1 has been reported to maintain Nrf2 regulation even in Nrf2 knockdown models [146]. The Nrf2 knockdown by small interfering RNA (siRNA) abrogated Re and Rg1-mediated protective effects, as evidenced by increased apoptotic rates, elevated ROS levels, and reduced antioxidant gene expression, supporting the importance of the Nrf2-activated signaling pathway in protecting against neuronal and hepatic damage [7,90,146,150,162]. KRG-mediated Nrf2 targeting HO-1 induction in astrocytes was effectively reduced in an Nrf2 knockdown model supporting the pivotal contribution of the Nrf2-HO-1 signaling axis in traumatic brain injury [163].

**Table 1:** Molecular mechanisms under Nrf2 knockdown.

Compounds	Dose range	Models	Mechanism action		Roles	Diseases	References
			Up-regulation	Down-regulation			
**Bioactive compounds – PPD**							
Rb1	5 – 20 uM	Human liver cells, HL-7702	Nrf2	Apoptotic rate	Anti-apoptosis	Cytotoxicity	[146]
Rd	40 – 100 uM	Human alveolar carcinoma cells, A549		Nrf2, HO-1, NQO1, GCLC, MRP1, MDR1	Anti-oxidative stress	Carcinoma	[147]
Rg3	10 uM	Human embryonic lung cells, HEL	BPDE-DNA adducts		Anti-oxidative stress	Lung tumorigenesis	[148]
**Bioactive compounds – PPT**							
Re	10 – 25 uM	Human neuroblastoma cells, SH-SY5Y	HO-1, NQO1, GCLC, ROS		Anti-oxidative stress	Skin aging	[149]
Rg1	10 – 40 uM	Mouse primary cultured hepatocytes	HO-1, NQO1, GCLC, Cyp2e1		Anti-oxidative stress	Liver injury	[7]
	10 – 40 uM	Mouse primary cultured hepatocytes	Nrf2, HO-1, NQO1, MRP2, MRP3, Ugt1a1	Cyp2e1	Anti-oxidative stress	Liver injury	[150]
	0.01 – 10 uM	Rat pheochromocytoma cells, PC12	Nrf2, ARE, HO-1, NQO1, GCLC, GCLM	LDH, ROS	Anti-oxidative stress	Ischemic/reperfusion	[90]
	0.001 – 10 uM	Human hepatocellular carcinoma cells, HepG2; human embryonic kidney cells, HEK293	Nrf2, HO-1, NQO1, GCLC, GCLM		Anti-oxidative stress	Hepatic necrosis	[91]
Notoginsenoside R1	10 – 40 uM	Human umbilical vein endothelial cells, HUVEC	p-Akt	p-IRS-1	Anti-oxidative stress	Metabolic disorders, cardiovascular disease	[136]
**Bioactive compounds – Oleanane**							
Rh3	1 – 30 uM	Human retinal pigment epithelial cells, ARPE-19		Nrf2, HO-1	Anti-oxidative stress	Radiation injury	[151]
	10 uM	Human endometrial stromal cells, HESC	Nrf2	HO-1, NQO1, SOD, LDH, mitochondrial depolarization	Anti-oxidative stress	Postpartum hemorrhage	[152]
**Extract compounds**							
Korean Red ginseng	250 μg/ml	Primary human brain astrocytes		Nrf2, HO-1, Tom20, PGC-1α	Anti-oxidative stress	Brain injury	[153]
Maltol	100 uM	Mouse chondrocytes	p65	Nrf2, HO-1, TNF-α, IL-6, MMP13, PGE2, NO, ADAMTS5	Anti-oxidative stress, anti-inflammation	Osteoarthritis	[154]
*P. japonicus* saponin	100 μg/ml	Alpha mouse liver 12 cells, AML12; human hepatic stellate cells, LX-2	p-Akt, p-GSK-3β, ROS	Nrf2	Anti-oxidative stress	Liver fibrosis	[155]
*P. quinquefolius* saponin	100 μg/ml	Aortic smooth muscle cells, AoSMC	Runx2	Nrf2	Anti-oxidative stress	Cardiovascular disease	[156]
Saponin fractions	25 – 100 μg/ml	Macrophage cells, RAW 264.7	IL-6, MCP-1		Anti-inflammation	Obesity	[157]
**Formulations**							
*P. ginseng* in Jinshui Huanxian	0.864 g/ml	Human lung fibroblast cells, MRC-5	α-SMA, FN1	Nrf2	Anti-oxidative stress	Pulmonary fibrosis	[158]
*P. notoginseng* saponin in Catalpol	10 μg/ml	Human hepatocyte cells, L-02	AST, ALT	SOD, GSH	Anti-oxidative stress	Hepatotoxicity	[159]

α-SMA, Alpha-smooth muscle actin; ADAMTS5, A disintegrin and metalloproteinase with thrombospondin motifs-5; ALT, Alanine aminotransaminase; ARE, Antioxidant response element; AST, Aspartate aminotransferase; BPDE, Benzo[a]pyrene diol epoxide; Cyp2e1, Cytochrome P450 family 2 subfamily E member 1; FN1, Fibronectin 1; GCLC, Glutamate-cysteine ligase catalytic subunit; GCLM, Glutamate-cysteine ligase modifier subunit; GSH, Glutathione; GSK-3β, Glycogen synthase kinase-3 beta; HO-1, Heme oxygenase-1; ILs, Interleukins; IRS-1, Insulin receptor substrate-1; LDH, Lactate dehydrogenase; MCP-1, Monocyte chemoattractant protein-1; MDR1, Multidrug resistance protein 1; MMPs, Matrix metalloproteinases; MRP, Multidrug resistance-associated protein; NO, Nitric oxide; NQO1, NAD(P)H quinone dehydrogenase 1; Nrf2, Nuclear factor erythroid 2-like 2; PGC-1α, Peroxisome proliferator-activated receptor gamma coactivator 1-alpha; PGE2, PGE, Prostaglandin E2; ROS, Reactive oxygen species; Runx2, Runt-related transcription factor 2; SOD, Superoxide dismutase; TNF-α, Tumor necrosis factor-alpha; Tom20, Translocase of outer mitochondrial membrane 20; Ugt1a1, Uridine diphosphate glucuronosyltransferase 1A1.

The crucial role of Nrf2 regulation is further demonstrated in Nrf2 knockout models, where the enhancement of downstream gene expression is significantly suppressed [164]. Ginseng failed to reverse the impaired effects in Nrf2 knockout models with reduced antioxidant gene expressions and antioxidant enzyme activities (Table 2) [165,168,169,171]. In the cerebral ischemia models, the down-regulation of LDH release, ROS production, aquaporin 4 (AQP4), and glutamate synthetase (GS) as well as inflammatory mediators including iNOS, COX-2, IL-1β, IL-6, IL-8 by ginseng were associated with increased Nrf2-mediated protection [171,[173], [174], [175]]. The attenuation of reactive gliosis and infarct size by KRG against cerebral hypoxic-ischemic damage was not seen in Nrf2 knockout mice, suggesting the protection conferred by KRG is an Nrf2-dependent neuroprotection in acute brain injury [169,175]. The Nrf2 knockout models exhibited a higher clinical disease index, cerebral edema, infarct volume, and lower locomotor activity, leading to exacerbated pathologies in disease models of ulcerative colitis, ischemic stroke, and hippocampal injury [168,169,171,173,175].

**Table 2:** Molecular mechanisms under Nrf2 knockout.

Compounds	Dose range	Models	Mechanism action		Roles	Diseases	References
			Up-regulation	Down-regulation			
**Bioactive compounds – PPT**							
Rg1	20 mg/kg	C57BL/6J mice	HO-1, GCLC, SOD, CAT, IL-1β, IL-6, IL-8, MMP3, MMP12, PAL1, p-p53, p16, p21, body weight	NQO1, GCLM, MDA, γ-H2AX, 8-OHdG, 4-HNE	Anti-oxidative stress, anti-inflammation	Senescence	[165]
**Extract compounds**							
American ginseng	75 mg/kg	C57BL/6 mice	COX-2, clinical disease index, colitis		Anti-inflammation	Ulcerative colitis	[166]
Hydrolyzed red ginseng	50 – 300 mg/kg	C57BL/6J mice		Spontaneous alteration, latency time	Anti-oxidative stress	Learning and memory ability	[167]
Korean Red ginseng	100 mg/kg	C57BL/6 mice	Body weight, cylinder test	HO-1, NQO1, SOD2, GPx, GS, AQP4, locomotor activity, corner test, infract volume, astrocytes	Anti-oxidative stress	Ischemic stroke	[168]
	100 mg/kg	C57BL/6 mice	IL-1β, iNOS, Iba-1, GS, AQP4, brain edema	HO-1, NQO1, SOD2, GPX1, IL-10, infract volume, astrocytes	Anti-oxidative stress, anti-inflammation	Cerebral ischemia	[169]
	100 mg/kg	C57BL/6 mice	Infract volume, astrogliosis, microgliosis		Anti-oxidative stress	Cerebral ischemia	[170]
	100 mg/kg	C57BL/6 mice	GS, AQP4, infract volume, brain edema	HO-1, NQO1, SOD2, GPx	Anti-oxidative stress	Hippocampal injury	[171]
Radix et rhizome ginseng	40 mg/kg	Mice		Papilloma, number of tumors, tumors diameter	Anti-oxidative stress	Cutaneous carcinoma	[172]

γ-H2AX, Gamma-H2A histone family member X; 4-HNE, 4-hydroxynonenal; 8-OHdG, 8-hydroxy-2′-deoxyguanosine; AQP4, Aquaporin 4; CAT, Catalase; COX-2, Cyclooxygenase-2; GCLC, Glutamate-cysteine ligase catalytic subunit; GCLM, Glutamate-cysteine ligase modifier subunit; GPx, Glutathione peroxidase; GS, Glutamate synthetase; HO-1, Heme oxygenase-1; Iba-1, Ionized calcium-binding adaptor molecule 1; ILs, Interleukins; iNOS, Inducible nitric oxide synthase; MDA, Malondialdehyde**;** MMPs, Matrix metalloproteinases; NQO1, NAD(P)H quinone dehydrogenase 1; PAL1, Phenylalanine ammonia-lyase 1; SOD, Superoxide dismutase.

Nrf2 expression is increased by inhibiting its degradation [176]. Nrf2 accumulation and persistent Nrf2 activation by mutation and silencing of Keap1 affect the dissociation of the Nrf2-Keap1 complex [177]. Table 3 shows the effect of the knockdown of Keap1 and p62 on Nrf2 regulation during ginseng treatment. Rh3 in Keap1 knockdown model accelerated the induction of Nrf2 expression along with HO-1 and NQO1 [152]. Besides, *P. quinquefolius* saponin in Keap1 knockdown model in aortic smooth muscle cells was associated with the downregulation of Runt-related transcription factor 2 (Runx2), controlling vascular cell calcification, and ameliorating oxidative stress [156]. In Keap1 knockdown models, there is an absence of interaction of Keap1 with Nrf2, resulting in Nrf2 accumulation to mitigate oxidative stress [180]. The dissociation of Keap1 and Nrf2 is also affected by the accumulation of p62 [46]. In p62 knockdown model, the expression of Keap1 is enhanced. This reversed ginseng-mediated Nrf2 induction, and the expression of downstream target genes associated with ameliorating oxidative stress, inflammation, and apoptosis [101,142,178,179]. On the other hand, Rd-mediated anti-pyroptosis through the Nrf2 pathway was not modulated in the p62 knockdown model, suggesting that partial deficiency caused by p62 knockdown may not be sufficient to reverse the effect of Rd on Keap1, Nrf2, or its downstream proteins [142].

**Table 3:** Molecular mechanisms under Nrf2-upperstream modifications.

Inducers	Compounds	Dose range	Models	Related molecular targets		Roles	Diseases	References
				Up-regulation	Down-regulation			
Kd-Keap1	Rh3	10 uM	Human leukemia monocytic cells, THP-1	Nrf2, HO-1, NQO1	SOD, ROS, LDH	Anti-oxidative stress	Postpartum hemorrhage	[152]
	*P. quinquefolius* saponin	100 μg/ml	Aortic smooth muscle cells, AoSMC	Nrf2	Keap1, Runx2	Anti-oxidative stress	Cardiovascular disease	[156]
Kd-p62	Rd	20 uM	Human leukemia monocytic cells, THP-1	ROS, IL-1β, damaged mitochondria		Anti-inflammation	Ulcerative colitis	[178]
		5 – 20 uM	Primary cortical neurons	Keap1	Nrf2, HO-1, NQO1, Trx1	Anti-oxidative stress	Cerebral ischemia/reperfusion	[142]
	Rh2	45 uM	Human cervical cancer cells, HeLa	ROS, Bax, PARP, Beclin 1, LC3B II	Nrf2, Bcl-2	Anti-apoptosis	Cervical cancer	[179]
	Ginseng root	1 μg/ml	Macrophage cells, RAW 264.7	ROS	Nrf2	Anti-oxidative stress	Immune system	[101]

Bax, Bcl-2-associated protein x; Bcl-2, B-cell lymphoma 2; HO-1, Heme oxygenase-1; ILs, Interleukins; Keap1, Kelch-like ECH-associated protein 1; LC3B II, Light chain 3 beta-II LDH, Lactate dehydrogenase; NQO1, NAD(P)H quinone dehydrogenase 1; Nrf2, Nuclear factor erythroid 2-like 2; PARP, Poly(ADP-ribose) polymerase; ROS, Reactive oxygen species; Runx2, Runt-related transcription factor 2; SOD, Superoxide dismutase; Trx1, Thioredoxin-1.

The loss of Nrf2 function has been observed in various diseases [181]. To address the underlying molecular mechanism underlying Nrf2 activation, some researchers have applied the Nrf2 chemical inhibitor ML385 to Nrf2 regulatory processes involved in pathological progression [182]. The role of Nrf2 in the effects of ginseng bioactive compounds and extracts was demonstrated by ML385 in *in vitro* and *in vivo* models (Table 4). ML385 is an Nrf2 inhibitor that binds to Neh1 and disrupts the DNA binding regulator, altering Nrf2 activation and downstream target gene expression [195]. ML385 was reported to reverse ginseng-induced Nrf2 and antioxidant gene expressions in cardiovascular disease models [183,184,186,193]. In recent studies, ML385 has been combined with ginseng bioactive compounds (Rg3, Rg1, Notoginsenoside R1, Rg5), extracts (ginseng-derived nanoparticles, *P. ginseng, P. japonicus* saponin), and formulations (*P. ginseng* Lizhong decoction) to determine the effects on oxidative stress markers, inflammatory markers, and ferroptosis markers [93,94,98,112,119,141,185,192]. Failure of Nrf2 activation due to the ML385 treatment contributed to the upregulation of some of the pro-inflammatory and pro-apoptotic markers, which were exacerbated in *in vitro* and *in vivo* models under ginseng treatment markers [93,94,99,112,115,119,138,141,185,187,191,192]. In addition, MG132 is a potent proteasome inhibitor that inhibits the degradation of Nrf2 by blocking the activity of β subunits of the 26S proteasome [196]. In the Keap1-Nrf2 system under normal conditions, the 26S proteasome constitutively degrades Nrf2 as a consequence of Keap1 binding to Neh2 [41]. Rg1 up-regulated Nrf2 and down-regulated Keap1 in bone marrow mesenchymal stem cells and this was reversed by MG132 [165]. Recent studies have reported that MG132 prolonged the Nrf2 half-life and accommodated the nuclear translocation, thereby providing positive feedback [[197], [198], [199]].

**Table 4:** Molecular mechanisms under chemical inhibitors.

Inhibitors	Compounds	Dose range	Models	Related molecular targets		Roles	Diseases	References
				Up-regulation	Down-regulation			
ML385	**Bioactive compounds – PPD**							
	Rb1	1 uM	Human liver cells, HL-7702		Nrf2	Anti-oxidative stress	Inflammation, rheumatic, neoplastic, neurodegenerative	[146]
	Rb3	2 – 8 uM	Rat cardiomyocytes cells, H9C2	Apoptotic rate	Nrf2, HO-1, TEAC	Anti-oxidative stress, anti-apoptosis	Myocardial infarction	[183]
	Rc	20 – 40 mg/kg	Swiss mice	MDA, TNF-α, IL-1β, CK-MB, Troponin T	Nrf2, HO-1, GCLC, GCLM, GSH	Anti-oxidative stress, anti-inflammation	Myocardial ischemic injury	[184]
	Rd	40 mg/kg	C57BL/6 mice	ROS, NLRP3, ASC, caspase 1, GSDMD-N, p20, pyroptotic cells	Nrf2, HO-1, NQO1, Trx1	Anti-oxidative stress, anti-pyroptosis	Cerebral ischemia/reperfusion	[142]
	Rg3	10 uM	Human neuroblastoma cells, SK-N-SH	Keap1, VP16	Nrf2, NQO1	Anti-oxidative stress, anti-inflammation	Neuroinflammation	[185]
	Ginsenosides (20S)-Rg3	25 uM	Rat cardiomyocytes cells, H9C2	Keap1	Nrf2, HO-1	Anti-oxidative stress	Cardiovascular disease	[186]
	Ginsenosides (20R)-Rg3	25 uM	Rat cardiomyocytes cells, H9C2	Keap1	Nrf2, HO-1	Anti-oxidative stress	Cardiovascular disease	[186]
	**Bioactive compounds – PPT**							
	Rg1	5 – 100 uM	Bone marrow mesenchymal stem cells	p21, p16		Anti-oxidative stress	Senescence	[165]
		10 uM	Mouse hippocampal neuronal cells, HT22	ROS, MDA, lipid peroxidation, IL-1β, IL-6	Nrf2, HO-1, NQO1, xCT, GPX4, TFR, DMT1, FSP1	Anti-inflammation, anti-oxidative stress, anti-ferroptosis	Neurodegenerative disease	[94]
		10 uM	Human hepatocellular carcinoma cells, HepG2	ROS, NLRP1, NLRP3, caspase 1, IL-1β, IL-6, AIM2	Nrf2, HO-1, NQO1	Anti-oxidative stress, anti-inflammation, anti-pyroptosis	Chronic liver disease	[93]
		10 mg/kg	Sprague-Dawley rats	Keap1, TNF-α, IL-6, osteoclast numbers, alveolar bone loss, local inflammation	Nrf2, TGF-β1, RUNX2, OCN, bone regeneration, osteogenesis, periodontal tissue recovery	Anti-oxidative stress, anti-inflammation	Periodontitis	[115]
		50, 200 uM	Human nucleus pulposus cells, NP	ROS, MDA, MMP3, Fe^2+^	Nrf2, SOD, GPx, FTL1, SLC7A11, collagen II	Anti-oxidative stress, anti-ferroptosis	Intervertebral disc degeneration	[187]
	Notoginsenoside R1	10 – 1000 uM	Mouse pre-odontoblast cells, mDPC6T	ROS, Bax, caspase 3, apoptotic rate	Nrf2, p62, MMP, p-Akt, LC3 II	Anti-apoptosis	Dental pulp injury	[188]
		50 – 100 mg/kg	Sprague Dawley rats	Keap1, TFRC, break up of myocardial fibers, expansion of endoplasmic reticulum	Nrf2, HO-1, GPX4, SLC7A11, FTH	Anti-oxidative stress, anti-ferroptosis	High-altitude myocardial injury	[98]
	**Bioactive compounds – Derivatives from PPD and PPT**							
	Rg5	5 – 10 uM	Human neuroblastoma cells, SK-N-SH	Keap1, ROS, p-IκB, NF-κB, TNF-α, IL-6, iNOS, COX-2, ICP0, ICP27, ICP24, VP16	Nrf2, HO-1, NQO1, GCLM, NFE2L2, SQSTM1, p-AMPKα	Anti-oxidative stress, anti-inflammation	Neuroinflammation	[185]
		0.2 – 5 uM	Human hepatocellular carcinoma cells, HepG2	Nrf2, p62, HO-1, SOD, GSH, GPX4, FTH1, FSP1	ROS, MDA, p-mTOR, NOX4, LC3 II	Anti-oxidative stress, anti-ferroptosis	Acute liver injury	[138]
		15 – 30 mg/kg	C57BL/6 mice	ROS, MDA, p-mTOR, NOX4, ALT, AST, liver damage	Nrf2, p62, HO-1, SOD, GSH, GPX4, FTH1, FSP1, SIRT1, LC3 II	Anti-oxidative stress	Acute liver injury	[138]
	**Extract compounds**							
	Ginseng-derived nanoparticles	5 – 20 μg/mL	Macrophage cells, RAW 264.7	TLR4, MYD88, p- p-ERK, P38, p-JNK, TNF-α, IL-6, NO, PGD2, PGE2	Nrf2, Keap1, p-p62, HO-1, NQO1, GCLC, GCLM, IL-10	Anti-oxidative stress, anti-inflammation	Intestinal inflammation	[119]
		0.5 – 80 μg/ml	Human hepatocyte cells, L-02	ROS, MDA, NF-κB, TNF-α, IL-1β, iNOS, COX-2, NO, Cyp2e1, HIF-1α	Nrf2, Keap1, HO-1, NQO1, GSH, OPA1	Anti-oxidative stress, anti-inflammation	Liver injury	[112]
	Gintonin	1 μg/ml	Striatal neuron progenitor cells, STHdh		Nrf2, HO-1	Anti-oxidative stress	Huntington's disease	[189]
	*P. ginseng*	40 μg/ml	Human bronchial epithelial cells, BEAS-2B	MDA, Fe^2+^, TNF-α, IL-1β, IL-6	GSH, GPX4	Anti-oxidative stress, anti-inflammation, anti-ferroptosis	Acute lung injury/acute respiratory distress syndrome	[190]
		25 – 100 μg/ml	Neuronal schwann cells, RSC96; Rat-mouse neuronal hybrid cells, ND7/23	ROS, MDA, TNF-α, IL-1β, IL-6, caspase 3, p-IκB, p-NF-κB, RAGE, Apoptosis rate	Nrf2, SOD, Bcl-2, PPARγ, JC10	Anti-oxidative stress, anti-inflammation, anti-apoptosis	Diabetic peripheral neuropathy	[191]
	*P. japonicus* saponin	-	Human hepatocellular carcinoma cells, HepG2	ROS	Nrf2, p62	Anti-oxidative stress	Alcoholic liver injury	[192]
	Panaxatriol saponin	10 μg/ml	Rat cardiomyocytes cells, H9C2	Bax, cytochrome *c*, caspase 3, PARP1		Anti-apoptosis	Myocardial ischemia-reperfusion injury	[193]
	Red ginseng	1 μg/ml	Human gastric adenocarcinoma cells, AGS	ROS, IL-8	HO-1, SOD1	Anti-oxidative stress	Atrophic gastritis	[194]
	**Formulations**							
	*P. ginseng* in Lizhong decoction	100 μg/ml	Human colorectal adenocarcinoma cells, Caco-2		Nrf2, SLC7A11, GPX4	Anti-oxidative stress, anti-ferroptosis	Ulcerative colitis	[141]
	*P. ginseng* in Shengmai injection	15 – 240 μl/l	Rat cardiomyocytes cells, H9C2	LDH, MDA, Keap1, Bax, caspase 3, CK	Nrf2, HO-1, SOD, Bcl-2	Anti-oxidative stress, anti-apoptosis	Cardiotoxicity	[109]
	*P. notoginseng* in Fufang Lurong Juangu capsule	50 – 500 μg/ml	Osteoblast cells		Nrf2, HO-1	Anti-oxidative stress	Osteoporosis	[111]
	*P. quinquefolium* in Yixin-Fumai granules	0.5 – 10 g/L	Cardiac muscle cells, HL-1	MDA, apoptotic rate, collage volume fraction	Nrf2, HO-1, SOD, CAT, GPx, HCN4	Anti-oxidative stress	Sick sinus syndrome	[110]
MG132	Rg1	5 – 100 uM	Bone marrow mesenchymal stem cells	Nrf2	Keap1	Anti-oxidative stress	Senescence	[165]

AIM2, Absent in melanoma 2; ALT, Alanine aminotransaminase; AMPKα, Adenosine monophosphate-activated protein kinase alpha; ASC, Apoptosis-associated speck-like protein containing a CARD; AST, Aspartate aminotransferase; Bax, Bcl-2-associated protein x; Bcl-2, B-cell lymphoma 2; CK, Creatine kinase; CK-MB, Creatine kinase-myocardial band; COX-2, Cyclooxygenase-2; Cyp2e1, Cytochrome P450 family 2 subfamily E member 1; DMT1, Divalent metal transporter 1; ERK, Extracellular signal-related kinase; FSP1, Ferroptosis suppressor protein 1; FTH1, Ferritin heavy chain 1; FTL1, Ferritin light chain 1; GCLC, Glutamate-cysteine ligase catalytic subunit; GCLM, Glutamate-cysteine ligase modifier subunit; GPx, Glutathione peroxidase; GPX4, Glutathione peroxidase 4; GSDMD-N, Gasdermin D N-terminal; GSH, Glutathione; HCN4, Hyperpolarization activated cyclic nucleotide gated potassium channel 4; HIF-1α, Hypoxia-inducible factor 1; HO-1, Heme oxygenase-1; ICPs, Infected-cell proteins; ILs, Interleukins; iNOS, Inducible nitric oxide synthase; IκB, Inhibitor of κB; JNK, C-Jun N-terminal kinase; Keap1, Kelch-like ECH-associated protein 1; LC3 II, Light chain 3 II; MDA, Malondialdehyde; MMPs, Matrix metalloproteinases; mTOR, Mammalian target of rapamycin; MYD88, Myeloid Differentiation primary response 88; NFE2L2, Nuclear factor, erythroid-derived 2-like 2; NF-κB, Nrf2 and nuclear factor-κB; NLRPs, Nucleotide-binding oligomerization domain (NOD)-like receptor pyrin domain containing proteins; NO, Nitric oxide; NOX4, NADPH oxidase 4; NQO1, NAD(P)H quinone dehydrogenase 1; Nrf2, Nuclear factor erythroid 2-like 2; OCN, Osteocalcin; OPA1, Optic atrophy 1; PARP, Poly(ADP-ribose) polymerase; PGD2, Prostaglandin D2; PGE2, PGE, Prostaglandin E2; PPARγ, Peroxisome proliferator-activated receptor gamma; RAGE, Receptor for advanced glycation end-products; ROS, Reactive oxygen species; Runx2, Runt-related transcription factor 2; SIRT1, Sirtuin 1; SLC7A11, Cystine/glutamate antiporter Solute carrier family 7 member 11; SOD, Superoxide dismutase; SQSTM1, Sequestosome 1; TEAC, Trolox equivalent antioxidant capacity; TFRC, Transferrin receptor C; TGF-β1, Transforming growth factor beta 1; TLR4, Toll-like receptor 4; TNF-α, Tumor necrosis factor-alpha; Trx1, Thioredoxin-1; VPs, Vacuolar protein sorting proteins.

## 8 Overview of clinical studies

This section is a review of the clinical studies on a total of 1744 and 3450 participants, respectively (Table 5). These studies consistently demonstrated the protective effects of ginseng in randomized, double-blind, placebo-controlled trials involving 3739 participants, with the majority receiving KRG and SMI. These clinical evaluations span a wide range of therapeutic areas, including inflammation, metabolic disorders, neurodegenerative diseases, cerebrovascular diseases, cardiovascular diseases, lung diseases, cancer, and COVID-19. The study populations were notably diverse, including healthy individuals, elderly patients, postmenopausal woman, and those with chronic or acute illness. This coverage of clinical contexts underscores the broad therapeutic interest in ginseng and its potential for systemic health benefits.

**Table 5:** Clinical efficacy of ginseng.

Compounds	Research design	Populations	Route of administration	Dose	Duration	Efficacy	Disease	References
Extract compounds								
Ginseng	Randomized, double-blind, placebo-controlled trial	30 postmenopausal women participants with osteopenia, ≥80 years old	Oral	1 g and 3 g	Single dose, 6 capsules per day, 12 weeks	↑ improvement knee symptoms, serum osteocalcin ↓ bone resorption, arthritis symptoms	Osteopenia and arthritis	[200]
Hydrolyzed ginseng	Randomized, double-blind, placebo-controlled trial	226 participants with prediabetes, ≥20 years old	Oral	960 mg	Single dose, once per day, 6 months	↑ HDL cholesterol, insulin sensitivity ↓ LDL cholesterol, fasting glucose, HbA1c, triglycerides	Prediabetes	[201]
Korean Red ginseng	Randomized, single-center, placebo-controlled trial	35 participants of men and postmenopausal women with type 2 diabetes ≥6 months ago, 45-75 years old	Oral	500 mg	Single dose, twice per day, 24 weeks	↑ improvement of serum levels of sex hormone binding globulin, lean body mass ↓ GDF-15	Type 2 diabetes	[202]
	Randomized, double-blind, placebo-controlled trial	28 participants with type 2 diabetes more than 6 months and those taking oral antidiabetic agents at least 3 months, 19-75 years old	Oral	500 mg	Single dose, twice per day, 24 weeks	↑ index of insulin resistance, improvement of diabetic peripheral neuropathy	Type 2 diabetes	[203]
	Randomized, placebo-controlled pilot trial	20 postmenopausal women participants with hypercholesterolemia, 50-70 years old	Oral	2 g	Single dose, twice per day, 4 weeks	↑ cholesterol metabolism, HDL cholesterol ↓ total cholesterol, LDL cholesterol, lathosterol, cholestanol	Hypercholesterolemia	[204]
	Randomized, double-blind, placebo-controlled phase III trial	219 participants with colorectal cancer, ≥60 years old	Oral	500 mg	Single dose, twice per day, 6 months	↑ improvement of fatigue, quality of life ↓ fatigue scores	Colorectal cancer	[205]
	Randomized, double-blind, placebo-controlled	25 participants with moderate chronic fatigue, ≥50 years old	Oral	3 g	Single dose, once per day, 6 weeks	↑ improvement of fatigue, anti-oxidative stress markers ↓ fatigue scores	Chronic fatigue	[206]
	Randomized, double-blind, placebo-controlled trial	33 participants of postmenopausal women, 46-49 years old	Oral	2 g	Single dose, once per day, 6 weeks	↑ mtDNA copy number ↓ LDL cholesterol, fatigue severity scale	Postmenopausal women	[207]
	Randomized, double-blind, placebo-controlled trial	29 participants of postmenopausal women diagnosed with gynecologic cancer, ≥20 years old	Oral	3 g	Single dose, once per day, 12 weeks	↓ menopause rating scale	Postmenopausal women	[208]
	Randomized, double-blind, placebo-controlled trial	20 participants with allergic rhinitis from scale I-IV severity, ±33.7 years old	Oral	3 mg/kg	Single dose, 4 weeks	↑ improvement in rhinorrhea, nasal, and eye itching ↓ symptoms, total serum IgE level, serum IL-4 level, eosinophil counts in nasal smears	Allergic rhinitis	[209]
	Randomized, prospective, controlled trial	13 participants with bile duct or pancreatic cancer, 62.2 ± 11.2 years old	Oral	3 g	Single dose, once per day, during adjuvant chemotherapy	↑ CD4^+^, quality of life scores ↓ fatigue, symptoms scores	Bile duct and pancreatic cancer	[210]
	Randomized, double-blind, placebo-controlled trial	174 participants with COVID-19 vaccine, ≥30 years old	Oral	3 g	Single dose, once per day, 2-22 weeks	↑ LDL cholesterol, anti-S-Ab, anti-N-Ab ↓ vitamin D	COVID-19	[211]
	Randomized, double-blind, placebo-controlled trial	60 participants with rheumatic diseases, mean age 50.9 ± 11,6 years and 97.5% were women	Oral	2 g	Single dose, once per day, 12-24 weeks	↑ improvement rate in fatigue, FACIT-F ↓ fatigue VAS	Rheumatic diseases	[212]
	Randomized, double-blind, placebo-controlled trial	100 participants with long COVID symptoms, 20-65 years old	Oral	3 g	Single dose, twice per day, 8 weeks	↑ improvement fatigue scores, cognitive function, better sleep quality ↓ brain fog, anxiety, physical exhaustion	COVID-19	[213]
	Randomized, double-blind, placebo-controlled trial	80 participants of healthy Japanese, 25-60 years old	Oral	2 g	Single dose, twice per day, 8 weeks	↑ improvement fatigue resistance, physical performance ↓ perceived fatigue, tiredness levels	Fatigue	[214]
	Randomized, double-blind, placebo-controlled trial	50 participants with mild chronic pancreatitis symptoms, 30-65 years old	Oral	3 g	Single dose, three times per day, 12 weeks	↑ quality of life scores ↓ fatigue, abdominal pain, digestive discomfort	Pancreatitis	[215]
*P. ginseng*	Randomized, double-blind, placebo-controlled trial	30 women participants with non-metastatic breast cancer	Oral	1 g	Single dose, once per day	↑ left ventricular ejection fraction at the eight cycles of chemotherapy ↓ left ventricular ejection fraction at the fourth cycle of chemotherapy	Breast cancer	[216]
	Randomized, double-blind, placebo-controlled trial	40 women participants with non-metastatic breast cancer	Oral	1 g	Single dose, once per day, 2 weeks	↑ social well-being, emotional well-being, functional well-being, breast cancer subscale ↓ physical-well being	Breast cancer	[217]
	Randomized, double-blind, placebo-controlled trial	30 men participants with recreational athletes, ≥18 years old	Oral	500 mg	Single dose, once per day, 2 weeks	↑ spare respiratory capacity of isolated mitochondria, mitochondrial function, bioenergetics, improvement of exercise performance ↓ fasting blood triacyl glycerides, non-esterified fatty acids, blood lactate levels, oxygen consumption, carbon dioxide production, IL-6, IL-8, IL-10	Metabolic health	[218]
*P. ginseng* sprout	Randomized, double-blind, placebo-controlled trial	80 participants with subjective memory impairment	Oral	450 mg	Single dose, twice per day, 4 weeks	↑ improvement in immediate recall total score, delayed recall, recognition on SVLT, total score and visuospatial-executive domain of the Korean version of MoCa-K, sleep quality ↓ AChE	Memory improvement	[219]
*P. notoginseng*	Randomized, double-blind, controlled trial	106 participants with ischemic stroke, 45-65 years old	Oral	3 g	Single dose, once per day, 4 weeks	↑ improvement in aspirin resistance ↓ TLR4, MYD88, NF-κB, IL-6, COX-2, TXA2	Stroke	[4]
	Randomized, prospective, controlled trial	53 participants with ischemic stroke, aspirin resistance, and undergoing secondary prevention therapy, 45-65 years old	Oral	3 g	Single dose, once per day, 4 weeks	↑ platelet sensitivity to aspirin ↓ platelet aggregation, TNF-α, IL-6, TLR4, MYD88, NF-κB	Ischemic stroke	[4]
*P. notoginseng* saponin	Randomized, single-center, assessor-blinded, two-arm, parallel-group trial	20 participants with stable coronary heart disease and chronic gastritis, 35-75 years old	Oral	100 mg	Single dose, twice per day, 2 months	↑ platelet inhibition rate, platelet cAMP, serum motilin, gastrin levels ↓ COX-1, CD62p, GPIIb-IIIa, platelet aggregation, dyspepsia, pain scores	Stable coronary heart disease, chronic gastritis	[220]
*P. quinquefolium*	Randomized, double-blind, placebo-controlled trial	7 healthy female participants, 40-60 years old	Oral	200 mg	Single dose, once per day, 6 h	↑ recruitment of prefrontal brain regions ↓ time delay between visual stimulus and brain response	Cognitive performance	[221]
	Randomized, double-blind, placebo-controlled trial	17 participants with head and neck cancer, ≥18 years old	Oral	500 mg	Single dose, twice per day, 8 weeks	↑ energy levels, functional well-being, improvement in fatigue symptoms ↓ fatigue scores	Head and neck cancer	[222]
	Randomized, double-blind, placebo-controlled trial	7 men participants with active college students, 22.4 ± 1.7 years old	Oral	1.6 g	Single dose, once per day, 3 weeks	↑ IL-10, improvement in recovery ↓ IL-4, plasma creatine kinase, lipid peroxidation, muscle soreness scores	Exercise-induced muscle damage	[223]
*P. quinquefolius* in polyphenolic rich supplement	Randomized, double-blind, placebo-controlled trial	52 healthy participants, 35-65 years old	Oral	50 mg	Single dose, twice per day, 4 weeks	↑ cognitive memory, prefrontal cortex activation, serum BDNF levels ↓ cognitive fatigue, emotional stress	Cognitive and emotional health	[224]
Red ginseng	Randomized, double-blind, placebo-controlled trial	72 participants with moderate perceived stress, 18-60 years old	Oral	200 mg	Single dose, once per day, 3 weeks	↑ first-attempt memory score, cognitive performance ↓ perceived stress scale, negative effect, depression score, visual memory errors, response latency in spatial planning	Moderate physiological stress	[225]
Red ginseng (KGC05pg)	Randomized, single-center, double-blind, placebo-controlled trial	49 participants with prediabetes, ≥19 years old	Oral	500 mg	Single dose, twice per day, 12 weeks	↑ C-peptide, insulinogenic index, glucagon, adiponectin, GLP-1 ↓ fasting blood glucose, blood glucose, HbA1c, glucose AUC, insulin resistance, dipeptidyl peptidase-4 levels	Prediabetes	[226]
White ginseng	Randomized, double-blind, placebo-controlled trial	42 participants with self-reported elevated stress or tension, 20-60 years old	Oral	495 mg	Two doses of three capsules, six capsules per day, 12 weeks	↑ tolerability ↓ self-reported stress, skin conductance, heart rate, stress relief, tension, anxiety symptoms	Physiological stress	[227]
**Formulations**								
Coptis root and ginseng	Randomized, double-blind, placebo-controlled trial	30 participants with diabetes, 18-65 years old	Oral	11 g	Single dose, once per day, 12 weeks	↑ Improvement in gut microba species abundance ↓ glycosylated hemoglobin, fasting blood glucose, postprandial blood glucose, IL-6, TNF-α, CRP, insulin resistance index	Type 2 diabetes	[228]
Ginseng berry saponin in Zhenyuan capsule	Randomized, double-blind, placebo-controlled trial	195 participants with prediabetes, 18-65 years old	Oral	50 mg	Single dose, three times per day, 4 weeks	↑ QUICKI index, high density lipoprotein cholesterol ↓ 2-h PG, fasting and 2-h postprandial levels of insulin and C-peptide, HOMA-IR index	Type 2 diabetes	[229]
Ginseng Guben Oral Liquid	Randomized, double-blind, placebo-controlled trial	120 participants with cancer-related fatigue, 35-70 years old	Oral	20 ml	Single dose, twice per day, 6 weeks	↑ improvement fatigue scores, physical activity, emotional well-being ↓ fatigue severity, sleep disturbances	Cancer	[230]
Ginseng in Daikenchuto	Randomized, open-label, controlled trial	20 participants with esophageal cancer resection, ≥20 years old	Oral	15 g	Single dose, once per day, 3 weeks	↑ gastrointestinal motility, recovery of bowel function ↓ postoperative ileus incidence, inflammatory markers, first flatus and bowel movement	Esophageal cancer	[231]
	Randomized, double-blind, placebo-controlled trial	17 participants with poststroke patients and functional constipation, ≥20 years old	Oral	15 g	Single dose, three times per day, 4 weeks	↑ defecation frequency, stool consistency, improvement of bowel movement ↓ constipation severity, abdominal bloating, discomfort	Poststroke functional constipation	[232]
	Randomized, open-label, controlled pilot trial	20 participants undergoing hepatectomy with postoperative abdominal pain, ≥20 years old	Oral	5 g	Single dose, three times per day, 2 weeks	↑ gastrointestinal motility, recovery speed ↓ abdominal pain, bloating scores	Postoperative abdominal symptoms	[233]
	Randomized, controlled trial	8 participants with colon cancer, ≥20 years old	Oral	5 g	Single dose, three times per day, 3 weeks	↑ bowel function ↓ abdominal pain, bloating scores	Colon cancer	[234]
Ginseng in Ninjin'yoeito	Randomized, double-blind, placebo-controlled trial	40 participants with anemia, ≥20 years old	Oral	7.5 g	Single dose, once per day, 10 days	↑ hemoglobin levels, hematocrit levels, ferritin levels ↓ Physical fatigue, affective fatigue, cognitive fatigue, overall fatigue, anxiety	Anemia	[235]
Ginseng in Tianjiang Xueshuantong Wan pill	Randomized, double-blind, placebo-controlled trial	36 participants with ischemia reperfusion, 64.80 ± 5.61 years old	Oral	-	-	↑ SOD level, NIHSS score ↓ serum levels of MDA, TNF, IL-6	Ischemia- reperfusion injury	[5]
*P. ginseng* in G115	Randomized, double-blind, placebo-controlled trial	100 participants with moderate to severe chronic obstructive pulmonary disease, ≥40 years old	Oral	200 mg	Single dose, twice per day, 24 weeks	↑ lung function, quality of life scores ↓ acute exacerbations, inflammatory markers	Chronic obstructive pulmonary disease	[236]
*P. ginseng* in Maekmundong-Tang	Randomized, double-blind, placebo-controlled trial	60 participants with nonspecific chronic cough, 19-75 years old	Oral	3 g	Single dose, three times per day, 4 weeks	↑ improvement in cough-related quality of life, lung function parameters ↓ cough severity, cough frequency, TNF-α, IL-6	Nonspecific chronic cough	[237]
*P. ginseng* in Renshen Yangrong Tang	Randomized, double-blind, controlled phase II trial	41 participants with cancer survivors and moderate to severe fatigue, 53.3 ± 6.6 years old	Oral	5 g	Single dose, twice per day, 6 weeks	↑ improvement of fatigue ↓ fatigue severity, symptoms	Cancer-related fatigue	[238]
*P. ginseng* in Saengmaeksan	Randomized, double-blind, placebo-controlled trial	9 men with college tennis player, 20-30 years old	Oral	110 ml	Single dose, seven times per day, 4 weeks	↑ IgA level ↓ ammonia level, ALT level, AST level, liver enzymes, IgM level	Fatigue	[239]
*P. ginseng* in Sailuotong	Randomized, double-blind, placebo-controlled trial	238 participants with mild to moderate vascular dementia or mixed with Alzheimer's disease, 40-85 years old	Oral	120 mg	Single dose, twice per day, 52 weeks	↑ cognitive function, daily living ability, quality of life ↓ neuropsychiatric symptoms, executive dysfunction	Vascular dementia, Alzheimer's disease with cerebrovascular disease	[240]
*P. ginseng* in Shengmai injection	Randomized, controlled trial	20 participants with coronary artery bypass grafting, 46-74 years old	Injection	60 ml	Single dose, once per day, 60 min	↑ cardiac output, stroke volume, ejection fraction	Coronary artery disease	[241]
	Randomized, controlled trial	44 participants with left ventricular ejection fraction ≥0.3, preoperative MoCA-BJ score ≥26, body mass index of 18-29 kg/m2, 40-69 years old	Intravenous	0.6 ml/kg	Single dose, 30 days	↑ blood gas, Ngb, HIF-1α, cognitive function ↓ blood pressure, NSE	Postoperative cognitive dysfunction	[242]
	Randomized, controlled trial	47 participants with non-small cell lung cancer patients undergoing chemotherapy, hemoglobin ≥90 g/L, 18-75 years old	Intravenous	100 ml	Single dose, once per day, 5 days	↓ adverse drug reactions	Lung cancer	[243]
	Randomized, controlled trial	83 participants with coronary artery bypass grafting, ≥18 years old	Injection and oral	100 ml and 3.6 g	Single dose, once per day, 30 days	↑ exercise tolerance	Coronary artery disease	[244]
	Randomized, controlled trial	29 participants with colorectal cancer at Duke's A, B, and C stage, 20-80 years old	Intravenous	80 ml	Single dose, once per day, 24 h	↑ α-SMA/CD31 ↓ FGF, PAI-1, VEGF	Tumor	[245]
*P. ginseng* in Shengmai Yin	Randomized, double-blind, multicenter controlled trial	160 participants with COVID-19 convalescent, 18-70 years old	Oral	10 ml	Single dose, three times per day, 2 weeks	↑ pulmonary function, cardiac function. quality of life scores ↓ fatigue scores, shortness of breath, chest tightness and palpitations, inflammatory markers, cardiac stress marker	Post-COVID-19	[246]
*P. ginseng* in Shenlian	Randomized, double-blind, placebo-controlled trial	16 participants with type 2 diabetes mellitus, 18-65 years old	Oral	1 g	Single dose, twice per day, 12 weeks	↑ improvement gut microbiota structure ↓ postprandial blood glucose, body mass index, fasting insulin levels, insulin resistance, C-reactive protein, gut microbiota species richness and evenness	Type 2 diabetes mellitus	[247]
*P. ginseng* in Tadalafil	Randomized, double-blind, placebo-controlled trial	50 male participants with erectile dysfunction, 38-69 years old	Oral	5 mg	Single dose, once per day, 3 months	↑ nitric oxide, improvement erectile function ↓ symptoms	Erectile dysfunction	[248]
*P. notoginseng* in Dan Qi Tong Mai Tablet	Randomized, double-blind, placebo-controlled phase I trial	84 healthy participants, 18-45 years old	Oral	90 to 5760 mg for a single dose, 360 to 2160 mg for multiple doses	single and multiple doses, twice per day, 2 weeks	↑ safety, tolerability, pharmacodynamic effects ↓ platelet aggregation, thromboxane B2 levels	Coronary heart disease	[249]
*P. notoginseng* in Fufang Xueshuantong capsule	Randomized, prospective, controlled pilot trial	38 participants with exudative age-related macular degeneration, ≥19 years old	Oral		Single dose, once per day, 3 months	↑ improvement of retinal structure, functional responsive ↓ abnormal blood vessel growth beneath the retina, disease activity, leakage	Exudative age-related macular degeneration	[250]
*P. notoginseng* in Jishe Quishi	Randomized, controlled trial	49 participants with rheumatoid arthritis, 24-75 years old	Oral	0.45 g	Single dose, three times per day	↑ improvement in clinical symptoms, quality of life, delay the progression of rheumatoid arthritis disease ↓ morning stiffness, joint pain, VAS score, ESR, CRP, RF, anti-CCP, TNF-α, IL-6, IL-7, Th17/Treg balance	Rheumatoid arthritis	[251]
*P. notoginseng* saponin in Xuesaitong soft capsule	Randomized, double-blind, placebo-controlled trial	1535 participants with ischemic stroke, 18-75 years old	Oral	60 mg	Single dose, twice per day, 3 months	↑ improvement in neurological deficits, Barthel index, EQ-5D score	Ischemic stroke	[252]
*P. notoginseng* in Xueshuantong capsule	Randomized, double-blind, multicenter, placebo-controlled trial	300 patients with acute myocardial infarction, 18-75 years old	Oral	-	Single dose, three times per day, 3 months	↑ left ventricular ejection fraction, cardiac function ↓ chest pain, fatigue, palpitations, cardiac stress, heart failure	Acute myocardial infarction	[253]
*P. quinquefolius* in Ceroboost	Randomized, double-blind, placebo-controlled trial	61 healthy participants, 18-40 years old	Oral	200 mg	Single dose, 2 weeks	↑ accuracy on the attention network test, performance, short-chain fatty acids acetate level, propionate level, butyrate level, beneficial gut bacteria ↓ commission errors on the RVIP, mental fatigue	Mood and cognition health	[254]

α-SMA, Alpha-smooth muscle actin; AChE, Acetylcholinesterase; ALT, Alanine aminotransaminase; AST, Aspartate aminotransferase; AUC, Area under the curve; BDNF, Brain-derived neurotrophic factor; CCP, Cyclic citrullinated peptide; CD, Cluster of differentiation; CD4^+^, CD4 positive T-cells; COX-1, Cyclooxygenase-1; COX-2, Cyclooxygenase-2; CRP, C-reactive protein; EQ-5D, EuroQoL group 5-dimension; ESR, Erythrocyte sedimentation rate; FACIT-F, Functional assessment of chronic illness therapy fatigue; FGF, Fibroblast growth factor; GDF-15, Growth differentiation factor 15; GLP-1, Glucagon-like peptide 1; GPIIb-IIIa, Glycoprotein integrin alpha-IIb/integrin beta-3; HbA1c, Hemoglobin A1c; HDL, High-density lipoprotein; HIF-1α, Hypoxia-inducible factor 1; HOMA-IR, Homeostatic model assessment-insulin resistance; ILs, Interleukins; LDL, Low-density lipoprotein; MDA, Malondialdehyde**;** MoCa-K, Montreal cognitive assessment-Korean; mtDNA, mitochondrial DNA; MYD88, Myeloid Differentiation primary response 88; N-Ab, Vaccine-induced neutralizing antibodies; NF-κB, Nrf2 and nuclear factor-κB; Ngb, Neuroglobin; NIHSS, National institutes of health stroke scale; NSE, Neuron-specific enolase; PAI-1, Plasminogen activator inhibitor-1; PG, Prostaglandin; QUICKI, Quantitative insulin sensitivity check index; RF, Rheumatoid factor; RVIP, Rapid visual information processing; S-Ab, Vaccine-induced antibodies to the spike protein receptor-binding domain; SOD, Superoxide dismutase; SVLT, Seoul verbal learning test; Th17, Helper T cells; TLR4, Toll-like receptor 4; TNF-α, Tumor necrosis factor-alpha; Treg, Regulatory T cells; TXA2, Thromboxane 2; VAS, Visual analog scale; VEGF, Vascular endothelial growth factor.

KRG has been evaluated in randomized, placebo-controlled clinical trials involving 599 participants with type 2 diabetes, postmenopausal symptoms, allergic rhinitis, rheumatic diseases, fatigue-related syndromes, pancreatitis, and COVID-19 (Table 5). In patients with type 2 diabetes, twice-daily administration of 500 mg of KRG for 24 weeks improved insulin resistance, diabetic peripheral neuropathy, and lean body mass [202,203]. Postmenopausal women receiving 2-3/day for 6 to 12 weeks showed reductions in low-density lipoprotein cholesterol levels, fatigue severity, and the menopause rating scale scores in both postmenopausal and premenopausal women [208,255]. In a 4-week trial in patients with allergic rhinitis, KRG significantly reduced serum IgE, IL-4, and eosinophil counts, indicating anti-inflammatory effects [209]. In fatigue-related conditions, including chronic fatigue, rheumatic conditions, and long COVID-19, KRG improved antioxidant capacity, cognitive function, sleep quality and symptom relief over 6 to 24 weeks [206,213,214]. In mild chronic pancreatitis, a dose of 3 g/day for 12 weeks was effective in alleviating abdominal pain and digestive discomfort [215]. A recent 24-week pilot study in COVID-19 vaccinated subjects demonstrated high rates of antibody formation, as well as an increase in sustained vaccine responses [256]. Furthermore, a phase III trial in 219 colorectal cancer patients demonstrated significant improvements in fatigue and health-related quality of life [205]. Taken together, these findings highlight the systemic therapeutic potential of KRG, particularly in modulating metabolic, inflammatory, and immune pathways, which align with downstream targets of the Nrf2 signaling pathways [225,257].

In randomized, double-blind, placebo-controlled trials, *P. ginseng* extracts administered for up to 2 weeks improved the breast cancer subscale of life assessments and enhanced social, emotional, and functional well-being and mitigated chemotherapy-induced cardiotoxicity in 70 patients with non-metastatic breast cancer (Table 5) [216,217]. A randomized clinical trial of 30 participants found that *P. ginseng* extract improved metabolic health by reducing lipid levels and inflammatory markers in athletes [258]. In another study with 80 individuals, administration of *P. ginseng* sprout extracts improved memory impairment and sleep quality [259]. *P. notoginseng* extracts reduced aspirin resistance and the NF-κB-mediated inflammatory pathway in patients with ischemic stroke [4,220].

Intravenous administration of SMI has been shown to enhance cardiac function in participants with coronary artery disease, as demonstrated in randomized controlled trials involving 103 participants undergoing coronary artery bypass grafting [241,244]. The previous study showed that SMI treatment in patients with cardiomyopathy led to increased Nrf2-mediated antioxidant gene expression and elevated levels of anti-apoptotic markers [109,241,244]. Improvements in chemotherapy-related fatigue and vascular remodeling and postoperative cognitive impairment due to cardiopulmonary bypass was alleviated by intravenous SMI [242,243,245]. Additionally, a randomized trial in 29 colorectal cancer patients demonstrated modulation of angiogenic markers after SMI infusion [245]. SMI is administered over a period of 60 min to 30 days and has shown clinical benefits in coronary artery disease, cognitive dysfunction, tumor progression. and lung cancer.

Clinical trials using a wide range of ginseng formulations, such as pills, capsules, injections and liquid, have also been reported. Formulations such as Coptis root and ginseng mixture, and *Zhenyuan* capsule containing ginseng berry saponin have been shown to effectively treat type 2 diabetes by lowering glycosylated hemoglobin, fasting blood glucose, postprandial blood glucose, and insulin resistance index and reducing pro-inflammatory markers [228,229]. Ginseng in *Tianjiang Xueshuantong Wan* pills exhibited beneficial effects on ischemic injury via its anti-oxidative and anti-inflammatory effects and *P. notoginseng* saponin in *Xuesaitong* soft capsules improved neurological deficits in a large trial involving 1535 subjects [5,252]. *P. notoginseng* radix et rhizome rhei in *Guanxin Danshen* dripping pills were shown to improve depressive symptoms in 1428 participants [260]. Recent study of Ginseng Guben oral liquid improved cancer-related fatigue symptoms in 120 participants, including physical activity and emotional well-being [230].

In this clinical study, SMI showed efficiency in short-term administration, while KRG can be applied to short and long-term administration to treat diseases. Preclinical studies suggest that diverse ginseng formulations warrant further exploration for their potential clinical benefits. Future clinical research should focus on well-designed, large-scale clinical trials using standardized ginseng formulations and unified protocols to improve the reliability of existing clinical evidence.

While Nrf2 activation confers cytoprotective effects, emerging evidence indicates that sustained or excessive activation of Nrf2 may contribute to tumor progression, metabolic reprogramming, and therapeutic resistance, highlighting its dual role as a double-edged regulator [261]. To date, however, there is no direct clinical evidence demonstrating that ginseng administration induces cancer metastasis or therapeutic resistance. Most preclinical studies suggest that ginseng and its ginsenosides exert anti-tumor effects, including enhanced chemosensitivity and reversal of drug resistance in various cancer models, likely mediated through their pleiotropic mechanisms of action [262]. In addition, certain ginsenosides exhibit biphasic dose–response characteristics, showing cytoprotective effects at low doses but potential cytotoxicity at higher concentrations [263]. Collectively, these findings highlight the need for further studies to define the therapeutic window and long-term safety of Nrf2-targeting strategies in the context of chronic disease management.

## 9 Future research directions

Ginseng, a potential traditional medicine, mediates Nrf2 activation, which in turn enhances the expression of the ARE-mediated gene battery. Upstream and downstream modifications of Nrf2 by ginseng play a key role in maintaining cellular homeostasis by regulating oxidative stress, inflammation, apoptosis, ferroptosis, and pyroptosis, which are disrupted in various diseases (Fig. 2). Bioactive compounds including PPD and PPT, ginseng extracts and their formulations have been shown to participate in Nrf2 signaling to inhibit the progression of several pathologies in *in vitro* and *in vivo* studies. For this reason, Nrf2 activation and stabilization play a significant role in drug development and therapeutics. Future research on ginseng-mediated Nrf2 activation should explore the molecular mechanisms in more detail, particularly focusing on how various bioactive compounds interact with the Nrf2 regulatory pathways. Thus, ginseng-mediated Nrf2 regulation offers promising advantages for current research. Additionally, pharmacokinetic profiling after ginseng administration and pharmacodynamics studies including efficacy and potency of various compounds, will strengthen the potential for drug development and therapeutics. Furthermore, understanding the synergistic effects of ginseng with other therapeutic agents could open new avenues for combination therapy in the treatment of chronic diseases. Larger-scale clinical trials to assess the long-term effects of ginseng extracts and formulations will provide valuable insights into their therapeutic potential and safety profile for medical application.

## CRediT authorship contribution statement

Bella Rhea Lavifa Sanjaya: Data curation, analysis, validation, visualization, writing original preparation, editing & review.

Lilik Duwi Wahyudi: Data curation, analysis, methodology, visualization.

Min Kyung Cho: Conceptualization, writing original preparation, review, editing, supervision & funding acquisition.

## Funding

This work was supported by the Basic Science Research Program in 10.13039/501100003725National Research Foundation of Korea (NRF) grants funded by the Korean government (10.13039/501100014188Ministry of Science and ICT) (2020R1A2C1012313) and funded by the Ministry of Education (RS-2025-25405230).

## Declaration of competing interest

The authors have declared no conflict of interest.

## Appendix A Supplementary data

Supplementary Fig. 1. Chemical structures of protopanaxadiol, protopanaxatriol, and derivatives of protopanaxadiol and protopanaxatriol.
