# Supplementary material for: Nrf2 as a therapeutic target of ginseng: A comprehensive review from preclinical evidence to clinical applications
Source: J Ginseng Res. 2026 Jun 24;50(5):101079. doi: 10.1016/j.jgr.2026.101079 (PMC13554444; doi:10.1016/j.jgr.2026.101079)
Supplement: Supplementary file 1 [file mmc1.docx]

**Supplementary information**

**Nrf2 as a therapeutic target of ginseng: A comprehensive review from preclinical evidence to clinical applications**

Bella Rhea Lavifa Sanjaya^a^, Lilik Duwi Wahyudi^a^, Min Kyung Cho^a^*

^a^Department of Pharmacology, College of Oriental Medicine, Dongguk University, Gyeongju 38066, Korea

* Corresponding authors

E-mail addresses

Bella Rhea Lavifa Sanjaya: [bellarhea78@gmail.com](mailto:bellarhea78@gmail.com)

Lilik Duwi Wahyudi: [lilik920101@gmail.com](mailto:lilik920101@gmail.com)

Min Kyung Cho: [mkcho@dongguk.ac.kr](mailto:mkcho@dongguk.ac.kr)

**Supplementary Table 1.** The role of ginseng on Nrf2 signaling pathway *in vitro*

| **Compounds** | **Dose range** | **Models** | **Mechanism action** | | **Roles** | **Diseases** | **References** |
| --- | --- | --- | --- | --- | --- | --- | --- |
|  |  |  | **Up-regulation** | **Down-regulation** |  |  |  |
| **Bioactive compounds - PPD** | | | | | | | |
| Rb1 | 1 – 20 uM | Human bronchial epithelial cells, BEAS-2B | Nrf2, HO-1, NQO1, GCLC, SOD, CAT, Bcl-2 | ROS, MDA, LDH, p-IκBα, NF-κB, TNF-α, IL-1β, IL-6, caspase 3, apoptotic rate | Anti-oxidative stress, anti-inflammation, anti-apoptosis | Chronic obstructive pulmonary disease | [1] |
|  | 20 uM | Macrophage cells, RAW264.7 | Nrf2, SLC7A11, GSH, GPX4, FTH | Keap1, ROS, TFR1, DMT1, Fe^2+^, NCOA4, ACSL-4, PTGS2, USP2 | Anti-oxidative stress, anti-ferroptosis | Atherosclerosis | [2] |
| Rb2 | 10 – 100 uM | Cardiomyoblast cells, H9C2 | Nrf2, HO-1 | LDH, CK-MB, cTn-1, apoptotic rate | Anti-apoptosis | Coronary heart disease | [3] |
| Rb3 | 2 – 8 uM | Rat myocardial cells, H9C2 | Nrf2, HO-1, Bcl-2, p-ERK | Keap1, ROS, Bax, apoptotic rate | Anti-apoptosis | Myocardial infarction | [4] |
| Rc | 20 uM | Mouse hippocampal neuronal cells, HT22 | pS40-Nrf2, SOD, CAT, GSH, GSR, GPx, GPX4, SIRT1, PGC1α, pY705-STAT3, pS727-STAT3, Foxo3a, mtFA, complex 1-V, ATP, NAD^+^ | pS253-Foxo3a | Anti-oxidative stress, anti-apoptosis | Vascular Dementia | [5] |
| Rd | 5 – 20 uM | Primary cortical neurons | Nrf2, HO-1, NQO1, Trx1 | Keap1, ROS, LDH, NLRP3, ASC, caspase 1, GSDMD-N, IL-1β, IL-18, TXNIP | Anti-pyroptosis | Cerebral ischemia/ reperfusion injury | [6] |
|  | 20 uM | Macrophage cells, RAW264.7 | Nrf2, SLC7A11, GSH, GPX4, FTH | Keap1, ROS, TFR1, DMT1, Fe^2+^, NCOA4, ACSL-4, PTGS2, USP2 | Anti-oxidative stress, anti-ferroptosis | Atherosclerosis | [2] |
| Rg3 | 10 – 40 uM | Human cardiomyocytes cells, C16; human cardiac myocytes cells, HCM | Nrf2, HO-1, SOD, SIRT1 | MDA, NF-κB, NLRP3, ASC, caspase 1, IL-1β, IL-18, TGF-β1, ANP, BNP, β-MHC, MyHc, collagen I | Anti-oxidative stress, anti-inflammation, anti-pyroptosis | Myocardial hypertrophy | [7] |
|  | 1 – 20 uM | Human embryonic lung cells, HEL | Nrf2, GST | BPDE-DNA adducts | Anti-oxidative stress | Lung tumorigenesis | [8] |
|  | 5 – 20 uM | Cardiomyoblast cells, H9C2 | Nrf2, HO-1, NQO1, GSH, GPX4, FTH, SLC7A11 | Keap1, ROS, MDA, ACSL-4, Fe^2+^ | Anti-oxidative stress, anti-ferroptosis | Myocardial ischemia/ reperfusion | [9] |
| Rh2 | 5 – 20 uM | Basophilic leukemia cells, RBL-2H3; rat peritoneal mast cells, RPMC | Nrf2 | Keap1, p-PI3K, MAPK, p-ERK1/2, p38, NF-κB, TNF-α, IL-1β, IL-4, IL-18, p-Lyn, Syk, LAT, p-PLCr2, Raf-1, uptake of calcium, histamine release, degranulation | Anti-inflammation | Asthma | [10] |
|  | 5 – 40 uM | Neonatal rat cardiomyocyte | Nrf2, HO-1, SOD, GPx | ROS, MDA, LDH, TNF-α, IL-1β, IL-18, NLRP3, ASC, caspase 1, CK-MB | Anti-oxidative stress, anti-inflammation, anti-pyroptosis | Myocardial ischemia-reperfusion injury | [11] |
| F2 | 0.1 – 40 uM | Cardiomyoblast cells, H9C2 | Nrf2, HO-1, NQO1, SOD, CAT, GSH, Bcl-2, p-PI3K, p-AKT | Keap1, ROS, LDH, MDA, caspase 3, CK-MB, CTn-1, apoptotic rate | Anti-oxidative stress, anti-apoptosis | Myocardial infarction | [12] |
| Compound K | 2.5 – 30 uM | Human retinal pigment epithelial cells, ARPE-19 | Nrf2, HO-1, Bcl-2, PARP, p-γH2AX | ROS, Bax, caspase 3, apoptotic rate | Anti-oxidative stress, anti-apoptosis | Retinal damage | [13] |
| **Bioactive compounds – PPT** | | | | | | | |
| Re | 1 – 10 uM | Human neuroblastoma cells, SH-SY5Y | Nrf2, HO-1, NQO1, GCLM, SOD, GSH, GPx, p-PI3K, p-AKT, p-ERK | ROS, MDA, Bax, mitochondrial dysfunction, caspase 9/8/3, apoptotic rate | Anti-oxidative stress, anti-inflammation, anti-apoptosis | Parkinson’s disease | [14] |
|  | 25 – 100 ug/ml | Porcine oocytes | Nrf2, SOD1, GSH, MMP | ROS, Bax, cytochrome c, caspase 3, apoptotic rate | Anti-oxidative stress, anti-apoptosis | Heat stress | [15] |
|  | 20 uM | Macrophage cells, RAW264.7 | Nrf2, SLC7A11, GSH, GPX4, FTH | Keap1, ROS, TFR1, DMT1, Fe^2+^, NCOA4, ACSL-4, PTGS2, USP2 | Anti-oxidative stress, anti-pyroptosis | Atherosclerosis | [2] |
| Rg1 | 0.01 – 10 uM | Rat pheochromocytoma cells, PC12 | Nrf2, HO-1, NQO1, GCLC, GCLM | Keap1 | Anti-oxidative stress | Ischemic/ reperfusion neuronal injury | [16] |
|  | 0.001 – 10 uM | Human hepatocellular carcinoma cells, HepG2; human embryonic kidney cells, HEK293 | Nrf2, ARE, HO-1, NQO1, CAT, GSH, GPx | ROS, cytochrome c, caspase 9/8/3, PARP-1/2, apoptotic rate | Anti-oxidative stress, anti-apoptosis | Hepatic necrosis | [17] |
|  | 5 – 20 uM | Rat pheochromocytoma cells, PC12 | Nrf2, HO-1, SOCS3 | ROS, TNF-α, IL-1β, iNOS, COX-2, GAS5 | Anti-oxidative stress, anti-inflammation | Depression | [18] |
|  | 10 uM | Human hepatocellular carcinoma cells, HepG2 | Nrf2, HO-1, NQO1 | ROS, NLRP1, NLRP3, caspase 1, IL-1β, IL-6, AIM2 | Anti-oxidative stress, anti-inflammation, anti-pyroptosis | Chronic liver disease | [19] |
|  | 10 uM | Mouse hippocampal neuronal cells, HT22 | Nrf2, HO-1, NQO1, xCT, GPX4, TFR, DMT1, FSP1 | ROS, MDA, lipid peroxidation, IL-1β, IL-6 | Anti-oxidative stress, anti-inflammation, anti-pyroptosis | Neurodegenerative disease | [20] |
|  | 10 – 500 uM | Neuroblastoma cells, SH-SY5Y | Nrf2, p62, GST, Beclin-1, cathepsin B, cathepsin L, Aβ, carbonyls, OGG1 | ROS, LC3 II, JC-1 | Anti-oxidative stress | Alzheimer’s disease | [21] |
|  | 50, 200 uM | Human nucleus pulposus cells, NP | Nrf2, SOD, GPx, FTL1, SLC7A11, collagen II | ROS, MDA, MMP3, Fe^2+^ | Anti-oxidative stress, anti-ferroptosis | Intervertebral disc degeneration | [22] |
| Rg2 | 10 – 500 uM | Neuroblastoma cells, SH-SY5Y | Nrf2, p62, HO-1, Beclin-1, cathepsin L, Aβ, carbonyls, OGG1 | ROS, LC3-II, cathepsin B, JC-1 | Anti-oxidative stress | Alzheimer’s disease | [21] |
| F1 | 3.125 – 50 uM | Human proximal tubule cells, HK-2 | Nrf2, HO-1, mitochondrial transmembrane potential | ROS, LDH, Bax, caspase 9/3, TGF-β1, collagen I, apoptotic rate | Anti-oxidative stress, anti-apoptosis | Diabetic nephropathy | [23] |
|  | 10 – 80 uM | Cardiomyoblast cells, H9C2 | Nrf2, HO-1, GCLM, SOD, GSH, GST, Bcl-2, p-AKT | Keap1, MDA, caspase 3/9 | Anti-oxidative stress, anti-apoptosis | Cardiotoxicity | [24] |
| Notoginsenoside R1 | 1 – 50 uM | Cardiomyoblast cells, H9C2 | Nrf2, HO-1, p-AMPK | Apoptotic rate, ANP, BNP | Anti-apoptosis | Diabetic cardiomyopathy | [25] |
|  | 10 – 1000 uM | Preodontoblast cells, mDPC6T | Nrf2, p62, p-Akt, LC3 II | ROS, Bax, depolarized mitochondria, caspase 3, apoptotic rate | Anti-apoptosis | Dental pulp injury | [26] |
|  | 10 – 40 uM | Human umbilical vein endothelial cells, HUVEC | Nrf2, HO-1, NQO1, GSH, GPX4, p-Akt, glucose uptake | ROS, MDA, lipid peroxidation, p-IRS-1 | Anti-oxidative stress | Metabolic disorders, cardiovascular disease | [27] |
|  | 0.1 – 10 uM | Porcine embryos | Nrf2, SOD2, CAT, GSH, Bcl-2, OCT4 | ROS, caspase 3, LC3B | Anti-oxidative stress, anti-apoptosis | Embryonic development | [28] |
|  | 20 uM | Macrophage cells, RAW264.7 | Nrf2, SLC7A11, GSH, GPX4, FTH | Keap1, ROS, TFR1, DMT1, Fe^2+^, NCOA4, ACSL-4, PTGS2, USP2 | Anti-oxidative stress, anti-ferroptosis | Atherosclerosis | [2] |
|  | 0.1 – 10 uM | Pig embryos | Nrf2, SOD2, CAT, GSH, GPx, PGC-1α, OCT4, lipid droplet, blastocyst | ROS, Bax, caspase 3, GRP78 | Anti-oxidative stress, anti-apoptosis | Oocyte maturation, embryonic development | [29] |
|  | 10 – 40 uM | Cardiomyoblast cells, H9C2 | Nrf2, HO-1, PGC-1α, SIRT3, Opa1, Mfn1, Mfn2 | ROS, Bax, caspase-3, Drp-1, Fis1, PINK1, Parkin, BNIP3 | Anti-oxidative stress, anti-apoptosis | Myocardial ischemia/reperfusion injury | [30] |
| **Bioactive compounds – Derivatives from PPD and PPT** | | | | | | | |
| Rg5 | 2 – 8 uM | Human ovarian cancer cells, A2780 | Nrf2, AKT, ERK | p-AKT, apoptotic rate | Anti-oxidative stress, anti-apoptosis | Cancer | [31] |
|  | 0.15 – 20 uM | Human neuroblastoma cells, SK-N-SH; epithelial cells, Vero | Nrf2, NFE2L2, SQSTM1, HO-1, NQO1, GCLM, p-AMPKα | Keap1, ROS, p-IκBα, p-NF-κB, iNOS, COX-2, TNF-α, IL-6, HSV-1 | Anti-oxidative stress, anti-inflammation | Neuroinflammation | [32] |
|  | 0.2 – 5 uM | Human hepatocellular carcinoma cells, HepG2 | Nrf2, p62, HO-1, SOD, GSH, GPX4, FTH1, FSP1 | ROS, MDA, p-mTOR, NOX4, LC3 II | Anti-oxidative stress, anti-ferroptosis | Acute liver injury | [33] |
| Rh4 | 2.5 – 80 uM | Rat skeletal muscle cells, L6 | Nrf2, SOD, GPx, p-AMPK, COX IV, SIRT1, PGC-1α, c-Myc, TFAM | MDA, COX-2, AGEs, HIF-1α | Anti-oxidative stress | Skeletal muscle aging | [34] |
| Rk1 | 5 – 125 uM | Human embryonic kidney cells, HEK293 | Nrf2, HO-1, GSH, Bcl-2 | ROS, MDA, Bax, caspase 3/9, apoptotic rate | Anti-oxidative stress, anti-apoptosis | Kidney disease | [35] |
|  | 100 – 400 uM | Human melanocyte cells, PIG1 | Nrf2, HO-1, SOD, CAT, GPx, p-AKT, Bcl-2 | Bax, caspase 3, apoptotic rate | Anti-oxidative stress, anti-apoptosis | Vitiligo | [36] |
|  | 10 – 30 uM | Rat pheochromocytoma cells, PC12 | Nrf2, HO-1, SOD, Bcl-2, SIRT3 | ROS, LDH, TNF-α, IL-1β, IL-6, Bax, caspase 3, apoptosis rate | Anti-oxidative stress, anti-inflammation, anti-apoptosis | Parkinson’s disease | [37] |
|  | 0.625 – 1.25 uM | Rat pheochromocytoma cells, PC12; primary neuronal cells | Nrf2, Keap1, HO-1, NQO1, Bcl-2, PARP, p-AMPK | LDH, Bax, caspase 3, apoptotic rate | Anti-oxidative stress, anti-apoptosis | Alzheimer’s disease | [38] |
| Rk3 | 2.5 – 10 uM | Rat pheochromocytoma cells, PC12 | Nrf2, Keap1, HO-1, NQO1, Bcl-2, PARP, p-AMPK | LDH, Bax, caspase 3, apoptotic rate | Anti-oxidative stress, anti-apoptosis | Alzheimer’s disease | [39] |
| **Extracts** | | | | | | | |
| 12-one-Pseudoginsenoside F11 | 10 – 100 uM | Human lung carcinoma cells, A549 | Nrf2, HO-1, SOD, GSH | MDA | Anti-oxidative stress | Lung cancer | [40] |
| Compound C-Y | 1 – 50 uM | Normal human dermal fibroblasts cells, NHDF | Nrf2, HO-1, NQO1, TGF-β1, TGF-β, p-Smad 2/3, p-NFATc1, Type I procollagen | ROS, p-ERK, p-38, p-JNK, TNF-α, IL-6, MMP-1, VEGF, p-c-fos, p-c-Jun, Smad7, NFATc1, melamine, MITF, TRP-1, TRP-2 | Anti-oxidative stress, anti-inflammation | Skin disorder | [41] |
| Forest ginseng | 6.25 – 25 ug/ml | Human foreskin fibroblasts cells, HFF-1 | Nrf2, SOD, CAT, GSH, GPx, COL1 | Keap1, ROS, MDA, SA-β-Gal, p53, p21, p16 | Anti-oxidative stress | Skin disease | [42] |
| Ginseng-derived nanoparticles | 5 – 20 ug/mL | Macrophage cells, RAW264.7 | Nrf2, Keap1, p-p62, HO-1, NQO1, GCLC, GCLM, IL-10 | TLR4, MYD88, p-ERK, p-P38, p-JNK, TNF-α, IL-6, NO, PGD2, PGE2 | Anti-oxidative stress, anti-inflammation | Inflammatory bowel disease | [43] |
|  | 0.5 – 80 ug/ml | Human hepatocyte, LO2 | Nrf2, Keap1, HO-1, NQO1, GSH, OPAI | ROS, MDA, NF-κB, TNF-α, IL-1β, iNOS, COX-2, NO, Cyp2e1, HIF-1α | Anti-oxidative stress, anti-inflammation | Liver injury | [44] |
| Ginseng berry kombucha fermented | 40 – 80 ug/ml | Human hepatocellular carcinoma cells, HepG2 | Nrf2, HO-1, SOD1, CAT, GPx, ADH, ALDH | Keap1, MDA, Cyp2e1 | Anti-oxidative stress | Liver damage | [45] |
| Ginseng oligosaccharides | - | Rat pheochromocytoma cells, PC12 | Nrf2, HO-1, GSH, CAT, LDH, Bcl-2 | ROS, Bax, cytochrome c | Anti-oxidative stress, anti-apoptosis | Neuronal oxidative injury | [46] |
| Ginseng root | 1 ug/ml | Macrophage cells, RAW264.7 | Nrf2, HO-1, GCLC, GCLM, IL-10, LC3 II, ATG-7, Beclin-1 | p62, ROS, p-AKT, NF-κB, TNF-α, IL-6, IL-1β, iNOS, COX-2, O_2_^-^, NO, TAK1, p-mTOR | Anti-oxidative stress, anti-inflammation | Inflammatory bowel disease | [47] |
| Ginsengin-S2 | 100 uM | Human lung carcinoma cells, A549 | Nrf2, SOD, GSH, HDAC2 | ROS, IL-8 | Anti-oxidative stress, anti-inflammation | Chronic obstructive pulmonary disease | [48] |
| Ginsenoside C-Mc | 1 – 20 uM | Normal human dermal fibroblasts cells, NHDF | Nrf2, HO-1, NQO1, GSH, IκBα, TGF-β1, Smad2/3 | ROS, LDH, p-MAPK, p-ERK, p-p38, p-JNK, NF-κB, TNF-α, IL-6, iNOS, MMP-1, MMP-3, VEGF, p-c-Jun, p-c-Fos | Anti-oxidative stress, anti-inflammation | Skin aging | [49] |
| Gintonin | 0.01 – 1 ug/ml | Striatal neuron progenitor cells, STHdh | Nrf2, HO-1, LPAR1, LPAR3, PLCβ3, β-III-tubulin | Apaf-1, caspase 3 | Anti-oxidative stress, anti-apoptosis | Huntington’s disease | [50] |
|  | 1 – 3 uM | Human colorectal adenocarcinoma cells, Caco-2 | Nrf2, HO-1, SOD, CAT, GPx, ZO-1, occluding, claudin-1 | ROS, MDA, TNF-α, IL-6 | Anti-oxidative stress, anti-inflammation | Intestinal injury | [51] |
| Korean Black ginseng | 25 – 50 ug/ml | Murince cell line, BV2 microglial cells | Nrf2, HO-1, TLR2, TLR4 | TNF-α, IL-1β, p38, MAPK, NF-κB, STAT3, NLRP3 | Anti-oxidative stress, anti-inflammation, anti-pyroptosis | Alzheimer’s disease | [52] |
| Korean Red ginseng | 25 – 100 ug/ml | Human monocytes cells, THP-1 | Nrf2, HO-1, IκBα | Keap1, NF-κB, CD11β, CD36 | Anti-oxidative stress, anti-inflammation | Chronic inflammation | [53] |
| Korean White ginseng | 50 – 1000 ug/ml | Human hepatoma cells, HepG2 | Nrf2, HO-1 | ROS | Anti-oxidative stress | Liver injury | [54] |
| Maltol | 25 – 100 uM | Mouse chondrocytes | Nrf2, HO-1, IκBα, Collagen II | TNF-α, IL-6, iNOS, COX-2, MMP-13, PGE2, NO, ADAMTS5, p65 | Anti-oxidative stress, anti-inflammation | Osteoarthritis | [55] |
|  | 2 – 8 uM | - | Nrf2, p62, HO-1, Bcl-2, SLC7A11, GSH, GPX4, NOX4, ATG14, ATG7, ATG5, PINK1, LC3 II/I | Keap1, MDA, COX-2, Bax, cytochrome c, caspase 3, Fe^2+^, ACSL-4, p53, p-PI3K, p-AKT, Parkin, NOX4 | Anti-oxidative stress, anti-inflammation, anti-apoptosis, anti-ferroptosis | Liver damage | [56] |
| Mountain-cultivated ginseng | 6.25 – 100 ug/ml | Primary human umbilical vein endothelial cells, HUVEC | Nrf2, Keap1, SOD, Bcl-2, PI3K, AKT, eNOS, VEGFA | ROS, MDA, Bax, cytochrome c, caspase 3 | Anti-oxidative stress, anti-apoptosis | Ischemic stroke | [57] |
| *P. ginseng* | 5 – 25 ug/ml | Human proximal tubule cells, HK-2 | Nrf2, HO-1, SOD, CAT, GPx | TNF-α, IL-1β, p-NF-κB, Bax, caspase 3 | Anti-oxidative stress, anti-inflammation, anti-apoptosis | Ischemia-reperfusion induced acute kidney injury | [58] |
| *P. ginseng* fruit | 50 – 200 ug/ml | Human periodontal ligament cells, HPDL | Nrf2, HO-1, ALP, COL1, OPN, RUNX2 | TNF-α, IL-1β, IL-6, iNOS, COX-2, NO, PGE2 | Anti-oxidative stress, anti-inflammation | Periodontal disease | [59] |
| *P. ginseng* metabolite | 0.05 – 500 uM | Sertoli cells, HSec | Nrf2, SOD, CAT, GPx, SIRT1 | Caspase 3, 8-OHdG | Anti-oxidative stress, anti-apoptosis | Male infertility | [60] |
| *P. ginseng* saponin | 20 ug/ml | Rat pheochromocytoma cells, PC12 | Nrf2, HO-1, SOD, CAT, GPx, p-ERK, Bcl-2 | ROS, p-38, p-JNK, Bax, cytochrome c, caspase 3/9 | Anti-oxidative stress, anti-inflammation, anti-apoptosis | Neurodegenerative disease | [61] |
| *P. japonicus* saponin | 100 ug/ml | Alpha mouse liver 12 cells, AML-12; human hepatic stellate cells line, LX-2 | Nrf2, p-GSK-3β, HO-1, NQO1, GCLC, p-AKT | ROS, collagen I, α-SMA | Anti-oxidative stress | Liver disease | [62] |
|  | 100 – 500 ug/ml | Human hepatoma cells, HepG2 | Nrf2, NQO1, GCLC, SQSTM1 | Keap1, ROS, Cyp2e1 | Anti-oxidative stress | Alcoholic liver injury | [63] |
| *P. notoginseng* saponin | 200 – 400 ug/ml | Mouse microvascular cerebral endothelial cells, bEnd.3 | Nrf2, HO-1, p-Akt, ZO-1, Claudin-5 | ROS, NF-κB, TNF-α, IL-1β, VCAM- 1 | Anti-oxidative stress, anti-inflammation | Cerebral diseases | [64] |
|  | 10 – 20 umol/l | Kupffer cells, KCs | Nrf2, ARE | Keap1, IRAK4, p-IKKα, p-IκBα, p-p65 | Anti-oxidative stress, anti-inflammation | Liver transplantation | [65] |
|  | 25 – 800 ug/ml | House ear institute-organ of Corti 1 cells, HEI-OC1 | Nrf2, HO-1, NQO1, GCLC, Bcl-2, p-AKT | ROS, caspase 3, apoptotic rate | Anti-oxidative stress, anti-apoptosis | Ototoxicity | [66] |
| *P. quinquefolius* saponin | 25 – 200 ug/ml | Aortic smooth muscle cells | Nrf2, HO-1, SM22α | ROS, ALP, Runx2, BMP2 | Anti-oxidative stress | Cardiovascular disease | [67] |
| Panaxydol | 10 – 40 ug/ml | Human lung epithelial cells, BEAS-2B | Nrf2, HO-1, MMP | Keap1, TNF-α, IL-1β, IL-6 | Anti-inflammation | Acute lung injury | [68] |
| Panaxadiol | 40 – 320 ug/l | Human hepatoma cells, HepG2 | Nrf2, HO-1, p-AMPK, PPARα | ROS, p-NF-κB, SREBP1C | Anti-oxidative stress, anti-inflammation | Non-alcoholic fatty liver disease | [69] |
|  | 10 ug/ml | Cardiomyoblast cells, H9C2 | Nrf2, HO-1, NQO1, PGC-1α, OPA1, Mfn1, Mfn2 | Keap1, ROS, MDA, TNF-α, IL-1β, IL-6, IL-18, Bax, cytochrome c, caspase 3/9, p-Drp1 | Anti-oxidative stress, anti-inflammation, anti-apoptosis | Septic cardiomyopathy | [70] |
| Rgx365 | 25 – 200 ug/ml | Primary human umbilical vein endothelial cells, HUVEC | Nrf2, HO-1 | NF-κB, TNF-α, IL-1β, iNOS, COX-2, NO, PGE, nitrite, p-STAT1 | Anti-oxidative stress, anti-inflammation | Vascular inflammatory disorders | [71] |
| **Formulations** | | | | | | | |
| *P. ginseng* in Lizhong decoction | 0.001 – 1000 ug/ml | Human colorectal adenocarcinoma cells, Caco-2 | Nrf2, SOD, SLC7A11, GSH, GPX4, occludin, claudin 1 | ROS, MDA, LDH, iron, GSSG | Anti-oxidative stress, anti-ferroptosis | Ulcerative colitis | [72] |
| *P. ginseng* in Shengmai injection | 60 – 240 ul/l | Cardiomyoblast cells, H9C2 | Nrf2, HO-1, SOD, Bax, caspase 3 | Keap1, MDA, LDH, Bcl-2, CK, cell apoptosis | Anti-apoptosis | Cardiomyopathy | [73] |
| *P. notoginseng* in Fufang Lurong Juangu capsule | 50 – 500 ug/ml | Osteoblast cells | Nrf2, HO-1, SOD, CAT, GPx, Bcl-2, COL1, Runx2, Osx, ALP, BMP2, OCN, ALP | ROS, cytochrome c, caspase 3 | Anti-oxidative stress, anti-apoptosis | Osteoporosis | [74] |
| *P. notoginseng* Radix Et Rhizoma in Yinao Fujian | 50 – 200 uM | Mouse microgial cells, BC2 | Nrf2, HO-1, NQO1, SOD, IκBα | Keap1, ROS, MDA, p-IκBα, p-p65, TNF-α, IL-6, iNOS, COX-2 | Anti-oxidative stress, anti-inflammation | Ischemic stroke | [75] |
| *P. quinquefolium* in Yixin-Fumai granules | 500 – 10.000 ug/ml | Cardiac muscle cells, HL-1 | Nrf2, HO-1, HCN4 | ROS | Anti-oxidative stress | Sick sinus syndrome | [76] |

β-MHC, Beta-myosin heavy chain; γ-H2AX, Gamma-H2A histone family member X; 8-OHdG, 8-hydroxy-2'-deoxyguanosine; ACSL-4, Acyl-CoA synthetase long-chain family member 4; ADAMTS5, A disintegrin and metalloproteinase with thrombospondin motifs-5;

AGEs, Advanced glycation end-products; AIM2, Absent in melanoma 2; ALP, Alkaline phosphatase; AMPK, Adenosine monophosphate-activated protein kinase; AMPKα, Adenosine monophosphate-activated protein kinase alpha; ANP, Auricular natriuretic peptide; Apaf-1, Apoptotic protease activating factor-1; ARE, Antioxidant response element; ASC, Apoptosis-associated speck-like protein containing a CARD; ATGs, Autophagy related-genes; Bax, Bcl-2-associated protein x; Bcl-2, B-cell lymphoma 2; BMP2, Bone morphogenetic protein 2; BNIP3, Bcl-2 interacting protein 3; BNP, Brain natriuretic peptide; BPDE, Benzo[a]pyrene diol epoxide; CAT, Catalase; CD, Cluster of differentiation; CK, Creatine kinase; CK-MB, Creatine kinase-myocardial band; c-Myc, cellular myelocytomatosis oncogene; COL1, Collagen type 1; COX-2, Cyclooxygenase-2; cTn-1, Cardiac troponin I; Cyp2e1, Cytochrome P450 family 2 subfamily E member 1; DMT1, Divalent metal transporter 1; Drp-1, Dynamin-related protein 1; ERK, Extracellular signal-related kinase; Fis1, Mitochondrial fission 1; Foxo, Forkhead box O; FSP1, Ferroptosis suppressor protein 1; FTH, Ferritin heavy chain;

FTL1, Ferritin light chain 1; GAS5, Growth arrest specific 5; GCLC, Glutamate-cysteine ligase catalytic subunit; GCLM, Glutamate-cysteine ligase modifier subunit; GPx, Glutathione peroxidase; GPX4, Glutathione peroxidase 4; GRP78, Glucose-regulated protein 78;

GSDMD-N, Gasdermin D N-terminal; GSH, Glutathione; GSK-3β, Glycogen synthase kinase-3 beta; GSSG, Glutathione disulfide; GST, Glutathione S-transferases; HCN4, Hyperpolarization activated cyclic nucleotide gated potassium channel 4; HDAC2, Histone deacetylase 2; HIF-1α, Hypoxia-inducible factor 1; HO-1, Heme oxygenase-1; HSV-1, Herpes simplex virus type 1; IKKα, Inhibitor of nuclear factor kappa B kinases; ILs, Interleukins; iNOS, Inducible nitric oxide synthase; IRAK4, Interleukin 1 receptor associated kinase 4; IRS-1, Insulin receptor substrate-1; IκB, Inhibitor of κB; JNK, C-Jun N-terminal kinase; Keap1, Kelch-like ECH-associated protein 1; LAT, Linker for activation of T cells; LC3 II, Light chain 3 II; LC3B, Light chain 3; LDH, Lactate dehydrogenase; LPARs, Lysophosphatidic acid receptors; Lyn, Lck/Yes-related novel tyrosine kinase; MAPK, Mitogen-activated protein kinase; MDA, Malondialdehyde; Mfns, Mitochondrial fusion proteins; MITF, Melanocyte inducing transcription factor; MMPs, Matrix metalloproteinases; mTOR, Mammalian target of rapamycin; MYD88, Myeloid Differentiation primary response 88; MyHc, Myocardial fibrosis-associated proteins; NAD, Nicotinamide adenine dinucleotide; NFATc1, NLR family pyrin domain containing 3; NFE2L2, Nuclear factor, erythroid-derived 2-like 2; NF-κB, Nrf2 and nuclear factor-κB; NLRPs, Nucleotide-binding oligomerization domain (NOD)-like receptor pyrin domain containing proteins; NO, Nitric oxide; NOX4, NADPH oxidase 4; NQO1, NAD(P)H quinone dehydrogenase 1; Nrf2, Nuclear factor erythroid 2-like 2; OCT4, Octamer-binding transcription factor 4; OGG1, 8-Oxoguanine DNA glycosylase 1; OPA1, Optic atrophy 1; OPN, Osteopontin; Osx, Osterix; PARP, Poly(ADP-ribose) polymerase; PGC-1α, Peroxisome proliferator-activated receptor gamma coactivator 1-alpha; PGD2, Prostaglandin D2; PGE2, PGE, Prostaglandin E2; PI3K, Phosphoinositide 3-kinase; PINK1, PTEN-induced putative kinase 1; PLCr2, Putative calcium-binding proteins; PLCβ3, Phospholipase C beta 3; PPARα, Peroxisome proliferator-activated receptor alpha; PTGS2, Prostaglandin-endoperoxide synthase 2; Raf-1, Rapidly accelerated fibrosarcoma-1; ROS, Reactive oxygen species; Runx2, Runt-related transcription factor 2; SA-β-Gal, Senescence-associated beta-galactosidase; SIRT1, Sirtuin 1; SLC7A11, Cystine/glutamate antiporter Solute carrier family 7 member 11; SM22α, Smooth muscle protein 22 alpha; SMA, Smooth muscle actin; Smad, Suppressor of mothers against decapentaplegic; SOCS3, Suppressor of cytokine signaling 3; SOD, Superoxide dismutase; SQSTM1, Sequestosome 1; SREBP1C, Sterol regulatory element-binding protein 1 C; STATs, Signal transducer and activator of transcriptions; Syk, Spleen tyrosine kinase; TAK1, Transforming growth factor beta-activated kinase 1; TFAM, Mitochondrial transcription factor A; TFR1, Transferrin receptor 1; TGF-β1, Transforming growth factor beta 1; TLR4, Toll-like receptor 4; TNF- α, Tumor necrosis factor-alpha; TRP-1, Tyrosinase related protein-1; Trx1, Thioredoxin-1; TXNIP, Thioredoxin interacting protein; USP2, Ubiquitin specific protease 2; VCAM-1, Vascular cell adhesion molecule 1; VEGF, Vascular endothelial growth factor; ZO-1, Zonula occludens-1.
**Supplementary Table 2**. The role of ginseng on Nrf2 signaling pathway *in vivo*

| **Compounds** | **Dose range** | **Models** | **Mechanism action** | | **Roles** | **Diseases** | **References** |
| --- | --- | --- | --- | --- | --- | --- | --- |
|  |  |  | **Up-regulation** | **Down-regulation** |  |  |  |
| **Bioactive compounds – PPD** | | | | | | | |
| Rb1 | 15 mg/kg | Sprague Dawley rats | Nrf2, SOD, p-p38, p-Akt | MDA, TNF-α, IL-1β, IL-6, D-lactate, DAO, endotoxin, 8-iso-PGF | Anti-oxidative stress, anti-inflammation | Intestinal ischemia/ reperfusion injury | [77] |
|  | 35 – 70 mg/kg | C57BL/6 mice | Nrf2, HO-1, SOD, CAT, SIRT1, longer immobility | TNF-α, IL-1β, IL-18, NLRP3, ASC, caspase 1 | Anti-oxidative stress, anti-inflammation, anti-pyroptosis | Depression | [78] |
|  | 20 mg/kg | Sprague Dawley rats | Nrf2, HO-1, NQO1, GCLC | p-IκBα, NF-κB, TNF-α, IL-1β, IL-6 | Anti-oxidative stress, anti-inflammation | Chronic obstructive pulmonary disease | [1] |
| Rb3 | 50 mg/kg | Sprague Dawley rats | Nrf2, HO-1, p-ERK, Bcl-2 | ROS, Bax, apoptotic rate | Anti-oxidative stress, anti-apoptosis | Myocardial infarction | [4] |
| Rc | 10 – 40 mg/kg | Swiss mice | Nrf2, HO-1, GCLC, GCLM, GSH | MDA, TNF-α, IL-1β, CK-MB, TNT, inflammatory cell, necrosis, myofibrillar fracture | Anti-oxidative stress, anti-inflammation | Myocardial ischemia | [79] |
|  | 20 mg/kg | C57BL/6J mice | Nrf2, HO-1, SOD, GSH, SIRT6, AST, ALT, PPARα | TNF-α, IL-6, H_2_O_2_, Acc, Fasn, SREBP1C | Anti-oxidative stress, anti-inflammation | Alcoholic liver disease | [80] |
| Rd | 10 – 40 mg/kg | C57BL/6 mice | Nrf2, HO-1, NQO1, Trx1, neurological impairment | Keap1, ROS, TXNIP, NLRP3, ASC, caspase 1, GSDMD-N, IL-1β, IL-18, MCAO/R, p20, brain infarction | Anti-oxidative stress, anti-pyroptosis | Cerebral ischemia/ reperfusion injury | [6] |
|  | 20 mg/kg | C57BL/6J mice | Nrf2, HO-1, GPX4, mitochondrial integrity, muribaculum intestinale | ROS, MDA, iron, firmicutes, faecalibaculum rodentium, liver steatosis and liver injury | Anti-oxidative stress, anti-ferroptosis | Metabolism-associated fatty liver disease | [81] |
| Rg3 | 30 mg/kg | Sprague-Dawley rats | Nrf2, HO-1, SIRT1 | NF-κB, NLRP3, ASC, caspase 1, IL-1β, IL-18, ANP, BNP, β-MHC, TGF-β1, heart weight, collagen | Anti-oxidative stress, anti-inflammation, anti-pyroptosis | Myocardial hypertrophy | [7] |
|  | 10 – 20 mg/kg | Wistar rats | Nrf2, SOD, Bcl-2 | ROS, MDA, LDH, IFNγ, IκBα, NF-κB, p65, TNF-α, IL-1β, IL-6, Bax, cytochrome c, caspase 3/9, apoptotic rate, AST, CHOP, XBP-1, kidney weight, blood nitrogen, creatinine | Anti-oxidative stress, anti-inflammation, anti-apoptosis | Acute kidney injury | [82] |
|  | 5 – 20 mg/kg | Sprague-Dawley rats | Nrf2, HO-1, GPX4, SLC7A11, improved ejection fraction and fractional shortening | Keap1, ROS, MDA, TNF-α, IL-1β, IL-6, Fe^2+^, infarct size | Anti-oxidative stress, anti-inflammation, Anti-ferroptosis | Myocardial ischemia/ reperfusion | [9] |
|  | 15 – 30 mg/kg | X57BL/6 mice | Nrf2, HO-1, GPX4, SLC7A11, FTH1, FLC, improved lung tissue | ROS, MDA, TNF-α, IL-1β, IL-6, COX-2, TFRC, alveolar hemorrhage, infiltration | Anti-oxidative stress, anti-ferroptosis | Hypoxia-induced pulmonary edema | [83] |
| Rh2 | 10 – 20 mg/kg | Sprague-Dawley rats | Nrf2, HO-1, SOD, GPx, LVEF, LVFS | MDA, LDH, TNF-α, IL-1β, IL-18, NLRP3, ASC, caspase 1, CK, CK-MB, LVEDD, LVESD, infarcted area | Anti-oxidative stress, anti-inflammation, anti-pyroptosis | Myocardial ischemia-reperfusion injury | [11] |
| F2 | 5 – 20 mg/kg | Sprague Dawley rats | Nrf2, HO-1, NQO1, SOD, CAT, GSH, Bcl-2, p-PI3K, p-Akt | Keap1, MDA, LDH, Bax, caspase 3, CK-MB, cTn-1 | Anti-oxidative stress, anti-apoptosis | Myocardial infarction | [12] |
| Compound K | 20 – 40 mg/kg | ICR mice | Nrf2, HO-1, SOD, GSH, Bcl-2, IDE, improved neuronal injury | Keap1, MDA, Bax, caspase 3, apoptotic rate, APP, Aβ, BACE1, PS1, cognitive decline | Anti-oxidative stress, anti-apoptosis | Alzheimer’s disease | [84] |
|  | 30 – 60 mg/kg | Kunming mice | Nrf2, HO-1, GCLM, SOD, GSH, Bcl-2, SIRT1 | MDA, TLR4, p-IκBα, NF-κB, TNF-α, IL-1β, IL-6, Bax, caspase 3/9, ALT, AST, ALP | Anti-oxidative stress, anti-inflammation, anti-apoptosis | Autoimmune hepatitis | [85] |
| **Bioactive compounds – PPT** | | | | | | | |
| Re | 10 – 40 mg/kg | C57BL/6J mice | Nrf2, HO-1, SOD, CAT, GSH, BDNF, SYP, PSD95, locomotor activity, memory | MDA, NLRP3, ASC, caspase 1, IL-1β, IL-18 | Anti-oxidative stress, anti-pyroptosis | Chronic stress | [86] |
|  | 10 – 25 mg/kg | C57BL/6 mice | Nrf2, GCLC, GCLM, GSH, p-JAK2, p-STAT3 | ROS, GSSG, NOX, AT1 receptor | Anti-oxidative stress | Memory impairments | [87] |
|  | 2 mg/kg | - | Nrf2, GCLC, GCLM, GSH | PKCδ, 5HT turnover rate, serotonergic behaviors | Anti-oxidative stress | Serotonin syndrome behavior | [88] |
| Rg1 | 20 mg/kg | Wistar rats | Nrf2, HO-1, SOD, CAT, GSH, GPx, p-AMPK, PGC-1α | ROS, LDH, TLR4, NF-κB, TNF-α, IL-1β, IL-6, NLRP3, ASC, MAP, CK-MB, AST, TBARS, heart damage, hypertrophy, blood glucose imbalance | Anti-oxidative stress, anti-inflammation, anti-pyroptosis | Diabetes | [89] |
|  | 10 – 40 mg/kg | C57BL/6J mice | Nrf2, HO-1, NQO1, GCLC, GCLM, methylation of H3K9 | Keap1, NLRP3, IL-1β, IL-18, ALT, AST, blood glucose level, hepatic cell damage | Anti-oxidative stress, anti-pyroptosis | Liver injury | [90] |
|  | 20 mg/kg | Sprague- Dawley rats | Nrf2, ATP, SOCS3, EZH2, behavior, immobility time | TNF-α, IL-1β, IL-6, GAS5, Iba-1, sucrose consumption | Anti-oxidative stress, anti-inflammation | Depression | [18] |
|  | 10 – 20 mg/kg | Wistar rats | Nrf2, HO-1, SOD, Bcl-2 | ROS, MDA, LDH, IFNγ, IκBα, p65, NF-κB, TNF-α, IL-1β, IL-6, Bax, cytochrome c, caspase 3/9, apoptotic rate, AST, CHOP, XBP-1, kidney damage | Anti-oxidative stress, anti-inflammation, anti-apoptosis | Acute kidney injury | [82] |
|  | 40 mg/kg | C57BL/6 male | Nrf2, p-GSK-3β, HO-1, NQO1, GCLC, GCLM, SOD, GSH, Bcl-2, p-Akt | ROS, MDA, caspase 3, C-PARP, apoptosis, Bad, p-FYN, testis injury | Anti-oxidative stress, anti-apoptosis | Testicular toxicity | [91] |
|  | 45 mg/kg | C57BL/6J mice | Nrf2, HO-1, NQO1, GCLC, GCLM, SOD, GSH, IL-10, SIRT1, HDL-C | MDA, p65, TNF-α, IL-1β, IL-6, ALT, AST, ALP, TBA, TBIL, TC, TG, LDL-C, body weight | Anti-oxidative stress, anti-inflammation | Liver injury | [92] |
|  | 5 – 20 mg/kg | C57BL/6J mice | Nrf2, HO-1, NQO1, xCT, GPX4, TFR, DMT1, FSP1 | ROS, MDA, lipid peroxidation, IL-1β, IL-6, behavioral and cognitive dysfunction | Anti-oxidative stress, anti-inflammation, anti-ferroptosis | Neurodegenerative disease | [20] |
|  | 5 – 20 mg/kg | C57BL/6J mice | Nrf2, HO-1 | Keap1, TNF-α, IL-1β, IL-6, NLRP1, NLRP3, caspase 1, ALT, AST, TGF-β1, AIM2, p-Smad, glycogen deposition, liver fibrosis | Anti-oxidative stress, anti-inflammation, anti-pyroptosis | Chronic liver disease | [19] |
|  | 10 mg/kg | Sprague-Dawley rats | Nrf2, TGF-β1, Runx2, OCN, bone regeneration, osteogenesis and periodontal tissue recovery | Keap1, TNF-α, IL-6, osteoclast numbers, alveolar bone loss, local inflammation | Anti-oxidative stress, anti-inflammation | Periodontitis | [93] |
|  | 20 – 40 mg/kg | Sprague-Dawley rats | Nrf2, SOD, GPx, GPX4, FTL1, SLC7A11, collagen II, DHI | MDA, MMP3, IVD | Anti-oxidative stress, anti-ferroptosis | Intervertebral disc degeneration | [22] |
| F1 | 25 – 50 mg/kg | Wistar rats | Nrf2, HO-1, GCLM, SOD, GSH, GST, p-Akt, Bcl-2 | Keap1, MDA, LDH, cytochrome c, caspase 3/9, CK-MB, c-TnT, BNP, myocardial damage | Anti-oxidative stress, anti-apoptosis | Cardiotoxicity | [24] |
| Notoginsenoside R1 | 12.5 – 50 mg/kg | Sprague-Dawley rats | Nrf2, HO-1, NQO1, GSH, Bcl-2, Bcl-xl, XIAP, c-IAP-1, c-IAP-2, p-AMPK, survivin, Sestrin-2 | ROS, MDA, Bax, caspase 3, Bad, apoptotic rate | Anti-oxidative stress, anti-apoptosis | Neurotoxicity | [94] |
|  | 50 – 100 mg/kg | Sprague-Dawley rats | Nrf2, HO-1, SLC7A11, GSH, GPX4, FTH1, blood pH levels, PaO_2_, SaO_2_, PaCO_2_, sodium level | Keap1, ROS, MDA, LDH, TNF-α, IL-1β, BNP, CK, CK-MB, TFR, iron content, myocardial damage, cardiac dysfunction | Anti-oxidative stress, anti-inflammation, anti-ferroptosis | High-altitude myocardial injury | [95] |
| **Bioactive compounds – Derivatives from PPD and PPT** | | | | | | | |
| Rg5 | 15 – 30 mg/kg | C57BL/6 mice | Nrf2, p62, HO-1, SOD, GSH, GPX4, FTH1, FSP1, SIRT1, LC3 II | ROS, MDA, p-mTOR, NOX4, ALT, AST, liver damage | Anti-oxidative stress | Acute liver injury | [33] |
| Rk1 | 1 – 10 mg/kg | APP/PS1 mice | Nrf2, SOD, GSH, Bcl-2, p-AMPK, cognitive function | MDA, caspase 3, escape latency, APP, p-tau | Anti-oxidative stress | Alzheimer’s disease | [38] |
| **Extracts** | | | | | | | |
| Fresh ginseng pulp | 450 mg/kg | BALB/c mice | Nrf2, Keap1, HO-1, NQO1, SOD, CAT, GPx, GSH, Bcl-2 | MDA, TNF-α, LPS, TLR4, MYD88, p-p65, p-IκB, p-NF-κB, p-ERK, p-JNK, p-p38, Bax, caspase 3, ALT, AST, body weight, food intake, index of liver, spleen and thymus | Anti-oxidative stress, anti-inflammation, anti-apoptosis | Liver injury | [96] |
| Ginseng-derived nanoparticles | 5 – 10 mg/ml | C57BL/6J mice | Nrf2, p-p62, GCLM, SOD | Keap1, MDA, p-p38, p-JNK, TNF-α, IL-6 | Anti-oxidative stress, anti-inflammation | Inflammatory bowel disease | [43] |
|  | 5 – 10 mg/ml | C57BL/6J mice | Nrf2, p-p62, HO-1, NQO1, GSH | MDA, TLR4, MYD88, NF-κB, TNF-α, IL-1β, IL-6, iNOS, COX-2, NO, AST, ALT, Cyp2e1, HIF, liver injury | Anti-oxidative stress, anti-inflammation | Liver injury | [44] |
| Ginseng flower | 1.000 g/kg | BALB/c mice | Nrf2, HO-1, NQO1, SOD1, SOD2, CAT, body weight, thymus health, immunity |  | Anti-oxidative stress | Immunosuppression | [97] |
| Ginseng leaf | 1.000 g/kg | BALB/c mice | Nrf2, HO-1, NQO1, SOD1, SOD2, CAT, body weight, thymus health, immunity | IFN-γ, TNF-α, IL-1β, IL-4, IL-6 | Anti-oxidative stress, anti-inflammation | Immunosuppression | [97] |
| Ginseng root | 1.000 g/kg | BALB/c mice | Nrf2, HO-1, NQO1, SOD1, SOD2, CAT, body weight, thymus health, immunity | IFN-γ, TNF-α, IL-1β, IL-4, IL-6 | Anti-oxidative stress, anti-inflammation | Immunosuppression | [97] |
|  | 200 – 400 mg/kg | Wister albino rats | Nrf2, GSH, heart function, kidney health | MDA, NO, NF-κB, iNOS, PKC, kidney damage, necrosis, pyknosis, macrophage activity | Anti-oxidative stress, anti-inflammation | Myocardial infarction | [98] |
|  | - | C57BL/6J mice | Nrf2, p-p62, HO-1, GCLC, LC3 II, ATG7 | p-JNK, NF-κB, TNF-α, IL-6, COX-2, colon injury | Anti-oxidative stress, anti-inflammation | Inflammatory bowel disease | [47] |
| Ginseng stem and leaf total saponin | 100 – 200 mg/kg | Mice | Nrf2, HO-1, SOD, CAT, XIAP, metabolism | MDA, NF-κB, Bax, TIMP, ALT, AST, γGT, liver injury | Anti-oxidative stress, anti-inflammation, anti-apoptosis | Liver injury | [99] |
| Gintonin | 25 – 100 mg/kg | C57BL/6NTac mice | Nrf2, HO-1, NQO1, GCLs, LPAR-1, LPAR-3, LPAR-5, LPAR-6, PLCβ3, IP3R, survival rate, body weight, β-III-tubulin, striatal SDH activity | Keap1, p-ERK, p-p38, p-JNK, p-IκBα, p-65, TNF-α, IL-1β, IL-6, iNOS, COX-2, caspase 3, Iba-1, OX-42, LPAR-2, LPAR-4, brain damage, MitoSOX intensity | Anti-oxidative stress, anti-inflammation, anti-apoptosis | Huntington’s disease | [50] |
|  | 100 mg/kg | Sprague-Dawley rats | Nrf2, Mn-SOD, Bcl-2, BDNF, SIRT1, MBP, GAD67, LPAR1, synaptophysin, cerebellar Purkinje cell | TNF-α, IL-1β, Bax, caspase 3, NMDAR1, blood glucose | Anti-oxidative stress, anti-inflammation, anti-apoptosis | Cerebellar impairments | [100] |
|  | 50 – 100 mg/kg | C57BL/6NTac mice | Nrf2, HO-1, NQO1, ZO-1, occluding behavioral activity | Keap1, IFN-γ, p-NF-κB, p-ERK, p-p38, p-JNK, TNF-α, IL-1β, IL-6, IL-17A, ICAM-1, VCAM-1, iNOS, COX-2, MCP-1, MIP-1α, RANTES, Iba-1, T-bet, RORγt, FOXP3, TGF-β, GFAP, PEXAM, CD3, CD4, CD8, inflammation, macrophages, brain microglia | Anti-oxidative stress, anti-inflammation | Multiple sclerosis | [101] |
|  | 50 – 100 mg/kg | BALB/c and C57BL/6 mice | Nrf2, HO-1, NQO1, LPAR1, LPAR3 | IL-1β, IL-6, iNOS, COX-2, caspase 3, Iba-1, GFAP, neuronal death, sum of seizure score | Anti-oxidative stress, anti-inflammation, anti-apoptosis | Epilepsy | [102] |
|  | 25 mg/kg | C57BL/6 mice | Nrf2, HO-1, NQO1, ZO-1, occluding, claudin-1, improved intestinal structure | ROS, MDA, TNF-α, IL-1β, IL-6, apoptotic cells | Anti-oxidative stress, anti-inflammation, anti-apoptosis | Intestinal injury | [51] |
| Heat-treated fresh ginseng | 40 mg/kg | SPF BALB/c mice | Nrf2, HO-1, SOD, GSH, GPx | MDA, TLR4, p-ERK, p-p38, p-JNK, TNF-α, ALT, AST | Anti-oxidative stress, anti-inflammation | Liver injury | [103] |
| Irradiated ginseng adventitious roots | 50 – 500 mg/kg | C57BL/6 mice | Nrf2, HO-1, NQO1, CAT, GSH, metabolism, body weight | Keap1, ROS, MDA, blood glucose, glucose tolerance, lipid metabolism | Anti-oxidative stress | Type 1 diabetes mellitus | [104] |
| Korean Black ginseng | 100 mg/kg | 5xFAD transgenic mice | Nrf2, HO-1, cognitive performance | TNF-α, IL-1β, IL-6, p38, MAPK, NF-κB, STAT3, NLRP3, ASC, caspase-1, amyloid-β deposition | Anti-oxidative stress, anti-inflammation, anti-pyroptosis | Alzheimer’s disease | [52] |
| Korean Red ginseng | 50 – 200 mg/kg | Sprague-Dawley rats | Nrf2, Keap1, p-Akt, BDNF | Body weight, serum corticosterone | Anti-oxidative stress | Depression | [105] |
| Maltol | 50 – 100 mg/kg | ICR mice | Nrf2, HO-1, SOD, CAT, GSH, p-PI3K, p-Akt | ROS, MDA, AChE, latency, swim path length | Anti-oxidative stress | Brain aging | [106] |
|  | 50 mg/kg | C57BL/6 mice | Nrf2, Collagen II | MMP-13, OA scores of cartilages | Anti-oxidative stress | Osteoarthritis | [55] |
|  | 50 – 100 mg/kg | C57BL/6 mice | Nrf2, HO-1, Bcl-2, p-PI3K, p-AKT, SLC7A11, GSH GPX4, ATG14, ATG7, ATG5, PINK1, Parkin, LC3 II/I | Keap1, p62, MDA, COX-2, Bax, cytochrome c, caspase 3, Fe^2+^, NOX4, ACSL-4, p53 | Anti-oxidative stress, anti-inflammation, anti-apoptosis, anti-ferroptosis | Liver damage | [56] |
| Mountain-cultivated ginseng | 20 mg/kg | C57BL/6J mice | Nrf2, GSH, p-ERK, ChAT, learning, memory and cognitive function | ROS, lipid peroxidation, NF-κB, GSSG, protein oxidation | Anti-oxidative stress, anti-inflammation | Brain aging | [107] |
|  | 12.5 – 50 ug/ml | Zebrafish larvae | Nrf2, SOD, PI3K, Akt, Bcl-2, eNOS, VEGFA, blood level, physical activity | MDA, Bax, cytochrome c, caspase 3, neurologic deficit, brain damage | Anti-oxidative stress, anti-apoptosis | Ischemic stroke | [57] |
| Nano ginseng | 20 mg/kg | Albino rats | Nrf2, CYP11A1, ARO, HSD-3b, StAR | DNA fragmentation | Anti-oxidative stress | Reproductive system | [108] |
| *P.* *ginsen*g | 300 mg/kg | Albino Wistar Wistar rats | Nrf2, HO-1, SOD, CAT, GSH, GPx, Bcl-2, TP, albumin, TG, AchE, paraxonase | MDA, LDH, IFN-γ, TNF-α, IL-1β, Bax, NO, 8-OHG, ACO, cholesterol | Anti-oxidative stress, anti-inflammation, anti-apoptosis | Hepatorenal injury | [109] |
|  | 100 mg/kg | C57BL/6 | Nrf2, NQO1, p-AMPK, claudin1, occludin, ZO-1 | TNF-α, IL-1β, IL-6, iNOS, COX-2, p-p65, p-p38, p-ERK, neutrophil infiltration, colon shortening | Anti-oxidative stress, anti-inflammation | Ulcerative colitis | [110] |
|  | 100 – 200 mg/kg | Sprague-Dawley rats | Nrf2, Bcl-2, PPARγ | TNF-α, IL-1β, IL-6, caspase 3, p-IκB, p-NF-κB, RAGE | Anti-oxidative stress, anti-inflammation, anti-apoptosis | Diabetic peripheral neuropathy | [111] |
|  | 25 mg/kg | C57BL/6 | Nrf2, HO-1, SOD, CAT, GPx | TNF-α, IL-1β, p-NF-κB, Bax, caspase 3 | Anti-oxidative stress, anti-inflammation, anti-apoptosis | Ischemia-reperfusion induced acute kidney injury | [58] |
| *P. japonicus* saponin | 60 – 100 mg/kg | Sprague- Dawley rats | Nrf2, HO-1, NQO1, SOD, CAT, GSH, GPx, MMP-2, MMP-9 | MDA, TNF-α, IL-1β, IL-6, TIMP1, TIMP2, TGF-β1, Smad2, Smad3, Smad4, LPO, serum UA, Cys C, β2-MG, collagen I, collagen III, collagen IV, fibronectin, α-SMA, kidney damage | Anti-oxidative stress, anti-inflammation | Renal fibrosis | [112] |
|  | 75 – 100 mg/kg | C57BL/6 mice | Nrf2, p62, NQO1, GCLC, SOD, CAT, GSH, AMPK-Acc/PPARα | ROS, MDA, TNF-α, IL-6, ALT, AST, TG, Cyp2e1, hepatic lipidosis | Anti-oxidative stress, anti-inflammation | Alcoholic liver injury | [63] |
| *P. notoginseng* polysaccharide | 25 – 100 mg/kg | Kunming mice | Nrf2, NQO1, SOD, CAT, GPx, ADH, Cyp2e1 | MDA, iNOS, COX-2, AST, ALT, TG | Anti-oxidative stress, anti-inflammation | Alcoholic liver damage | [113] |
| *P. notoginseng* saponin | 50 – 200 mg/kg | C57BL/6 mice | Nrf2, HO-1, SOD, GSH | MDA, IL-6, CRP, TGF-β1, blood glucose, kidney damage | Anti-oxidative stress | Diabetic nephropathy | [114] |
|  | 80 mg/kg | Sprague- Dawley rats | Nrf2, GSH, GPX4, behavioral and motor function | MDA, TNF-α, IL-1β, IL-6, Fe^2+^, LPO, MPO, neurological impairment | Anti-oxidative stress, anti-inflammation, anti-ferroptosis | Cerebral ischemia–reperfusion injury | [115] |
|  | 100 mg/kg | Sprague- Dawley rats | Nrf2, HO-1, SOD, GSH, SIRT1, Bcl-2, neurological function | ROS, MDA, Bax, caspase 3, infarct volume, neurological deficit | Anti-oxidative stress, anti-apoptosis | Cerebral ischemia/ reperfusion injury | [116] |
| Panaxadiol | 10 – 20 mg/kg | Wistar rats | Nrf2, HO-1, NQO1, SOD, CAT, GPx | 4-HNE, kidney damage | Anti-oxidative stress | Chronic kidney disease | [117] |
|  | 80 mg/kg | C57BL/6 mice | Nrf2, HO-1, SOD, Cpt1, Cpt2, PPARα, PPARγ | MDA, p-NF-κB, TNF-α, IL-1β, IL-6, NLRP3, TC, LDL, HDL, TG, ALT, AST, Acc, Fas, SREBP1C, body weight, liver damage, blood glucose | Anti-oxidative stress, anti-inflammation | Non-alcoholic fatty liver disease | [69] |
|  | - | C57BL/6J mice | Nrf2, HO-1, NQO1, SOD, GSH, Bcl-2, Cyc1, NDUFV1, ATP5F1, ATP5A1, Mfn1, Mfn2, OPA1, PGC1-α | Keap1, MDA, LDH, TNF-α, IL-1β, IL-6, IL-18, Bax, cytochrome c, caspase 3, apoptosis, CK-MB, NT-proBNP, HMGB1, GSSG, DRP-1, heart index | Anti-oxidative stress, anti-inflammation, anti-apoptosis | Cardiomyopathy | [70] |
|  | 20 mg/kg | C57BL/6 mice | Nrf2, SOD2, GPX4, PGC-1α, TFAM, PINK1, Parkin, OPA1, Mfn1, Mfn2 | Keap1, TNF-α, IL-6, Bax, caspase 3, ACSL-4, PTGS2, p-DRP-1 | Anti-oxidative stress, anti-inflammation, anti-apoptosis, anti-ferroptosis | Sepsis cardiomyopathy | [70] |
| Red ginseng | 100 – 400 mg/kg | ICR mice | Nrf2, HO-1, NQO1, SOD, CAT, GPx, repaired heart | Keap1, ROS, MDA, LDH, TNF-α, IL-6, CK, CK-MB, c-TnT, cTn-1, AST, heart damage | Anti-oxidative stress, anti-inflammation | Myocardial ischemia | [118] |
|  | 200 – 400 mg/kg | ICR mice | Nrf2, HO-1, SOD, p-P13K, p-Akt, Bcl-2, hormonal balance | Keap1, MDA, Bax, caspase 3/9, p53, p21, p16, body weight, level FSH | Anti-oxidative stress, anti-apoptosis | Premature ovarian failure | [119] |
|  | 500 mg/kg | DBA/2 mice | Nrf2, HO-1, Bcl-2 | TFR | Anti-oxidative stress, anti-apoptosis | Cardiomyopathy | [120] |
| Sterilized ginseng pulp | 450 mg/kg | BALB/c mice | Nrf2, Keap1, HO-1, NQO1, SOD, CAT, GPx, GSH, Bcl-2 | MDA, TNF-α, LPS, TLR4, MYD88, p-p65, p-IκB, p-NF-κB, p-ERK, p-JNK, p-p38, Bax, caspase 3, ALT, AST, body weight, food intake, index of liver, spleen and thymus | Anti-oxidative stress, anti-inflammation, anti-apoptosis | Liver injury | [96] |
| **Formulations** | | | | | | | |
| *P. ginseng* in Lizhong decoction | 1.820 – 5.460 mg/kg | C57BL/6J mice | Nrf2, SOD, SLC7A11, GSH, GPx, occludin, claudin-1, repaired colon | MDA, GSSH, TNF-α, IL-1β, IL-6, iron, body weight, disease activity | Anti-oxidative stress, anti-ferroptosis | Ulcerative colitis | [72] |
| *P. ginseng* in Shengmai injection | 2.7 – 10.8 ml/kg | Sprague- Dawley rats | Nrf2, HO-1, SOD, CAT, improve pathological changes | Keap1, MDA, LDH, CK, apoptotic rate, heart damage, fibrosis | Anti-oxidative stress, anti-apoptosis | Cardiomyopathy | [73] |
|  | 9 ml/kg | Sprague- Dawley rats | Nrf2, SOD, GPX4 | Fe^2+^, ACSL-4, myocardial injury | Anti-oxidative stress, anti-ferroptosis | Myocardial ischemia/ reperfusion injury | [121] |
| *P. ginseng* in SPTC | 130 mg/kg | C57BL/6 mice | Nrf2, HO-1, NQO1, Trxn, GLUT4, antioxidant | Keap1, HOMA-IR, liver weight, glucose tolerance, insulin | Anti-oxidative stress | Type 2 diabetes mellitus | [122] |
| *P. notoginseng* in Danshen Dripping pill | 170 – 340 mg/kg | Sprague-Dawley rats | Nrf2, SOD, GPX1, AQP1 | TNF-α, IL-1β, IL-6, ICAM-1, MMP-9, lung injury | Anti-oxidative stress, anti-inflammation | High-altitude pulmonary edema | [123] |
| *P. notoginseng* in Danshen Hedansanqi Tiaozhi Tang | 350 – 1.400 mg/kg | Sprague- Dawley rats | Nrf2, HO-1, SOD, CAT, GPx, T-AOC, liver function | MDA, TC, TG, LDL-C, AST, ALT, body weight, liver injury | Anti-oxidative stress | Non-alcoholic fatty liver disease | [124] |
| *P. notoginseng* in Guan Xin Dan Shen | 45 – 180 mg/kg | C57BLKS/J mice | Nrf2, HO-1, NQO1, γ-GCS, p-Akt, Bcl-2, insulin sensitivity, cardiac function | LDH, Bax, caspase 3/9, apoptotic rate, LDL, LVIDd, ANP, BNP, β-MHC, α-MHC, CK-MB, AST body weight, blood glucose, triglycerides, cholesterol, heart damage | Anti-oxidative stress, anti-apoptosis | Diabetic cardiomyopathy | [125] |
| *P. notoginseng* in Tianhuang | 100 mg/kg | C57BL/6J mice | Nrf2, HO-1, NQO1, GCLC, Trx1, SOD, GSH | Keap1, MDA, TC, TG, LDL-C, ALT, AST | Anti-oxidative stress | Non-alcoholic fatty liver disease | [126] |
| *P. notoginseng* Radix Et Rhizoma in Yinao Fujian | 0.21 – 1.64 mg/kg | Sprague-Dawley rats | Nrf2, HO-1, NQO1, SOD, IκBα | Keap1, MDA, p-IκBα, p-p65, TNF-α, IL-6, iNOS, COX-2, infarct volume | Anti-oxidative stress, anti-inflammation | Ischemic stroke | [75] |

α-MHC, Alpha-myosin heavy chain; β2-MG, Beta-2 microglobulin; β-MHC, Beta-myosin heavy chain; γ-GCS, Gamma-glutamylcysteine synthetase; γGT, Gamma-glutamyl transferase; 4-HNE, 4-hydroxynonenal; 5HT, 5-hydroxytryptamine; 8-iso-PGF, 8-isoprostane-prostaglandin F2α; 8-OHG, 8-iso-prostaglandin F; Acc, acetyl-CoA carboxylase; AChE, Acetylcholinesterase; ACO, Aconitase; ACSL-4, Acyl-CoA synthetase long-chain family member 4; AIM2, Absent in melanoma 2; ALP, Alkaline phosphatase; ALT, Alanine aminotransaminase; AMPK, Adenosine monophosphate-activated protein kinase; ANP, Auricular natriuretic peptide; APP, Amyloid precursor protein; AQP1, Aquaporin-1; ARO, Aromatase; ASC, Apoptosis-associated speck-like protein containing a CARD;

AST, Aspartate aminotransferase; AT1, Angiotensin 1; ATGs, Autophagy related genes; ATP5A1, ATP synthase F1 subunit alpha 1; ATP5F1, ATP synthase F1 subunit gamma; Aβ, Amyloid beta; BACE1, Beta-site APP cleaving enzyme 1; Bad, Bcl-2 associated death promoter; Bax, Bcl-2-associated protein x; Bcl-2, B-cell lymphoma 2; Bcl-xl, B-cell lymphoma-extra large; BDNF, Brain-derived neurotrophic factor; BNP, Brain natriuretic peptide; CAT, Catalase; CDs, Cluster of differentiations; ChAT, Choline acetyltransferase; CHOP, C/EBP homologous protein; CK, Creatine kinase; CK-MB, Creatine kinase-myocardial band; COX-2, Cyclooxygenase-2; Cpt, Carnitine palmitoyl transferase; CRP, C-reactive protein; cTn-1, Cardiac troponin I; c-TnT, Cardiac troponin T; Cyc1, Cytochrome c1;

CYP11A1, Cytochrome P450 family 11 subfamily A member 1; Cyp2e1, Cytochrome P450 family 2 subfamily E member 1; Cys C, Cystatin C; DAO, Diamine oxidase; DHI, 5,6-Dihydroxyindole; DMT1, Divalent metal transporter 1; Drp-1, Dynamin-related protein 1; eNOS, Endothelial nitric oxide synthase; ERK, Extracellular signal-related kinase; EZH2, Enhancer of zeste homolog 2; Fas, Fas cell surface death receptor; Fasn, Fatty acid synthase; FLC, Free light chain; FOXP3, Forkhead box P3; FSH, Follicle-stimulating hormone;

FSP1, Ferroptosis suppressor protein 1; FTH, Ferritin heavy chain; FTH1, Ferritin heavy chain 1; FTL1, Ferritin light chain 1; FYN, Fyn proto-oncogene; GAD67, Glutamic acid decarboxylase 67; GAS5, Growth arrest specific 5; GCLC, Glutamate-cysteine ligase catalytic subunit; GCLM, Glutamate-cysteine ligase modifier subunit; GLUT4, Glucose transporter type 4; GPx, Glutathione peroxidase;

GPX4, Glutathione peroxidase 4; GSDMD-N, Gasdermin D N-terminal; GSH, Glutathione; GSK-3β, Glycogen synthase kinase-3 beta;

GSSG, Glutathione disulfide; GST, Glutathione S-transferases; HDL-C, High-density lipoprotein cholesterol; HIF, Hypoxia-inducible factor; HMGB1, High mobility group box 1; HO-1, Heme oxygenase-1; HOMA-IR, Homeostatic model assessment-insulin resistance;

HSD-3b, 3β-hydroxysteroid dehydrogenase; IAPs, Inhibitor of apoptosis proteins; Iba-1, Ionized calcium-binding adaptor molecule 1; ICAM-1, Intercellular adhesion molecule 1; IDE, Insulin-degrading enzyme; IFNγ, Interferon gamma; ILs, Interleukins; iNOS, Inducible nitric oxide synthase; IVD, Intervertebral disc; IκB, Inhibitor of κB; IκBα, Inhibitor of κB alpha; JAK2, Janus kinase 2; JNK, C-Jun N-terminal kinase; Keap1, Kelch-like ECH-associated protein 1; LC3 II, Light chain 3 II; LDH, Lactate dehydrogenase; LDL, Low-density lipoprotein; LDL-C, Low-density lipoprotein cholesterol; LPARs, Lysophosphatidic acid receptors; LPO, Lipid peroxidation; LPS, Lipopolysaccharide; LVED, Left ventricular end-diastolic; LVEDD, Left ventricular end-diastolic dimension; LVEF, Left ventricular ejection fraction; LVESD, Left ventricular end-systolic dimension; LVFS, Left ventricular fractional shortening; LVIDd, Left ventricular internal diameter in diastole; MAP, Mean arterial pressure; MAPK, Mitogen-activated protein kinase; MBP, Myelin basic protein; MCAO/R, Middle cerebral artery occlusion/reperfusion; MCP-1, Monocyte chemoattractant protein-1; MDA, Malondialdehyde; Mfns, Mitochondrial fusion proteins; MIP-1α, Macrophage inflammatory protein-1 alpha; MitoSOX, Mitochondrial superoxide indicator; MMPs, Matrix metalloproteinases; Mn-SOD, Manganese superoxide dismutase; MPO, Myeloperoxidase; mTOR, Mammalian target of rapamycin; MYD88, Myeloid Differentiation primary response 88; NDUFV1, NADH:ubiquinone oxidoreductase core subunit V1; NF-κB, Nrf2 and nuclear factor-κB; NLRP3, Nucleotide-binding oligomerization domain (NOD)-like receptor pyrin domain containing protein 3; NMDAR1, N-methyl-D-aspartate receptor subunit 1; NO, Nitric oxide; NOX, NADPH oxidase; NOX4, NADPH oxidase 4; NQO1, NAD(P)H quinone dehydrogenase 1; Nrf2, Nuclear factor erythroid 2-like 2; NT-proBNP, N-terminal pro B-type natriuretic peptide; OA, Osteoarthritis; OCN, Osteocalcin; OPA1, Optic atrophy 1; PaCO_2_, Partial pressure of carbon dioxide; PaO_2_, Partial pressure of oxygen; PARP, Poly(ADP-ribose) polymerase; PEXAM, Physical examination; PGC-1α, Peroxisome proliferator-activated receptor gamma coactivator 1-alpha; PI3K, Phosphoinositide 3-kinase; PINK1, PTEN-induced putative kinase 1;

PKC, Protein kinase C; PKCδ, Protein kinase C delta; PLCβ3, Phospholipase C beta 3; PPARα, Peroxisome proliferator-activated receptor alpha; PPARγ, Peroxisome proliferator-activated receptor gamma; PS1, Presenilin 1; PSD95, Postsynaptic density protein 95; PTGS2, Prostaglandin-endoperoxide synthase 2; RAGE, Receptor for advanced glycation end-products; RANTES, Regulated on activation normal T cell expressed and secreted; RORγt, Retinoic acid receptor-related orphan receptor gamma T; ROS, Reactive oxygen species; Runx2, Runt-related transcription factor 2; SDH, Succinate dehydrogenase; Serum UA, Serum uric acid; SIRTs, Sirtuins; SLC7A11, Cystine/glutamate antiporter Solute carrier family 7 member 11; SMA, Smooth muscle actin; Smads, Suppressor of mothers against decapentaplegics; SOCS3, Suppressor of cytokine signaling 3; SOD, Superoxide dismutase; SREBP1C, Sterol regulatory element-binding protein 1 C; StAR, Steroidogenic acute regulatory protein; STATs, Signal transducer and activator of transcriptions; SYP, Synaptophysin; TBA, Total bile acids; TBARS, Thiobarbituric acid reactive substances; T-bet, T-box transcription factor TBX21;

TBIL, Total bilirubin; TC, Total cholesterol; TFAM, Mitochondrial transcription factor A; TFR, Transferrin receptor; TFRC, Transferrin receptor C; TG, Triglycerides; TGF-β1, Transforming growth factor beta 1; TIMP, Tissue inhibitor of metalloproteinases; TLR4, Toll-like receptor 4; TNF- α, Tumor necrosis factor-alpha; TNT, Troponin T; TP, Total protein; Trx1, Thioredoxin-1; Trxn, Thioredoxin; TXNIP, Thioredoxin interacting protein; VCAM-1, Vascular cell adhesion molecule 1; VEGFA, Vascular endothelial growth factor A; XBP-1, X-box binding protein 1; xCT, Cystine/glutamate transporter; XIAP, X-linked inhibitor of apoptosis protein; ZO-1, Zonula occludens-1.

References

1.	Li Z, Li L, Lv X, Hu Y, and Cui K. Ginseng Saponin Rb1 Attenuates Cigarette Smoke Exposure-Induced Inflammation, Apoptosis and Oxidative Stress via Activating Nrf2 and Inhibiting NF-kappaB Signaling Pathways*.* Int J Chron Obstruct Pulmon Dis 2023; 18: 1883-97.

2.	Zhao Y, Zheng G, Yang S, Liu S, Wu Y, Miao Y, et al. The plant extract PNS mitigates atherosclerosis via promoting Nrf2-mediated inhibition of ferroptosis through reducing USP2-mediated Keap1 deubiquitination*.* Br J Pharmacol 2024; 181(23): 4822-44.

3.	Li Y and Zhang W. Effect of Ginsenoside Rb2 on a Myocardial Cell Model of Coronary Heart Disease through Nrf2/HO-1 Signaling Pathway*.* Biol Pharm Bull 2022; 45(1): 71-6.

4.	Sun J, Yu X, Huangpu H, and Yao F. Ginsenoside Rb3 protects cardiomyocytes against hypoxia/reoxygenation injury via activating the antioxidation signaling pathway of PERK/Nrf2/HMOX1*.* Biomed Pharmacother 2019; 109: 254-61.

5.	Wu Y, Zhang Z, and Lian X. Ginsenoside Rc mitigates hippocampal neuronal damage and cognitive impairment in vascular dementia rats via the pY705-Stat3/Foxo3a and pS727-Stat3/GRIM-19 pathways*.* J Ginseng Res 2025; 49(6): 702-13.

6.	Yao Y, Hu S, Zhang C, Zhou Q, Wang H, Yang Y, et al. Ginsenoside Rd attenuates cerebral ischemia/reperfusion injury by exerting an anti-pyroptotic effect via the miR-139-5p/FoxO1/Keap1/Nrf2 axis*.* Int Immunopharmacol 2022; 105: 108582.

7.	Ren B, Feng J, Yang N, Guo Y, Chen C, and Qin Q. Ginsenoside Rg3 attenuates angiotensin II-induced myocardial hypertrophy through repressing NLRP3 inflammasome and oxidative stress via modulating SIRT1/NF-kappaB pathway*.* Int Immunopharmacol 2021; 98: 107841.

8.	Xiong J, Yuan H, Fei S, Yang S, You M, and Liu L. The preventive role of the red gingeng ginsenoside Rg3 in the treatment of lung tumorigenesis induced by benzo(a)pyrene*.* Sci Rep 2023; 13(1): 4528.

9.	Zhong G, Chen J, Li Y, Han Y, Wang M, Nie Q, et al. Ginsenoside Rg3 attenuates myocardial ischemia/reperfusion-induced ferroptosis via the keap1/Nrf2/GPX4 signaling pathway*.* BMC Complement Med Ther 2024; 24(1): 247.

10.	Xu C, Li L, Wang C, Jiang J, Li L, Zhu L, et al. Effects of G-Rh2 on mast cell-mediated anaphylaxis via AKT-Nrf2/NF-kappaB and MAPK-Nrf2/NF-kappaB pathways*.* J Ginseng Res 2022; 46(4): 550-60.

11.	Fan ZX, Yang CJ, Li YH, Yang J, and Huang CX. Ginsenoside Rh2 attenuates myocardial ischaemia‑reperfusion injury by regulating the Nrf2/HO‑1/NLRP3 signalling pathway*.* Exp Ther Med 2023; 25(1): 35.

12.	Cui Y, Wu J, Wang Y, Li D, Zhang F, Jin X, et al. Protective effects of ginsenoside F(2) on isoproterenol-induced myocardial infarction by activating the Nrf2/HO-1 and PI3K/Akt signaling pathways*.* Phytomedicine 2024; 129: 155637.

13.	Park C, Cha HJ, Song KS, Kim HS, Bang E, Lee H, et al. Nrf2-mediated activation of HO-1 is required in the blocking effect of compound K, a ginseng saponin metabolite, against oxidative stress damage in ARPE-19 human retinal pigment epithelial cells*.* J Ginseng Res 2023; 47(2): 311-8.

14.	Qiao J, Zhao Y, Liu Y, Zhang S, Zhao W, Liu S, et al. Neuroprotective effect of Ginsenoside Re against neurotoxininduced Parkinson's disease models via induction of Nrf2*.* Mol Med Rep 2022; 25(6): 215.

15.	Liu S, Ju AQ, Duan AY, Zhan CL, Gao LP, Ma X, et al. Presence of ginsenoside re during in vitro maturation protects porcine oocytes from heat stress*.* Reprod Domest Anim 2022; 57(12): 1572-83.

16.	Chu SF, Zhang Z, Zhou X, He WB, Chen C, Luo P, et al. Ginsenoside Rg1 protects against ischemic/reperfusion-induced neuronal injury through miR-144/Nrf2/ARE pathway*.* Acta Pharmacol Sin 2019; 40(1): 13-25.

17.	Gao Y, Chu SF, Zhang Z, Ai QD, Xia CY, Huang HY, et al. Ginsenoside Rg1 prevents acetaminophen-induced oxidative stress and apoptosis via Nrf2/ARE signaling pathway*.* J Asian Nat Prod Res 2019; 21(8): 782-97.

18.	Li J, Gao W, Zhao Z, Li Y, Yang L, Wei W, et al. Ginsenoside Rg1 Reduced Microglial Activation and Mitochondrial Dysfunction to Alleviate Depression-Like Behaviour Via the GAS5/EZH2/SOCS3/NRF2 Axis*.* Mol Neurobiol 2022; 59(5): 2855-73.

19.	Zhou H, Liu Y, Su Y, Ji P, Kong L, Sun R, et al. Ginsenoside Rg1 attenuates lipopolysaccharide-induced chronic liver damage by activating Nrf2 signaling and inhibiting inflammasomes in hepatic cells*.* J Ethnopharmacol 2024; 324: 117794.

20.	Kong L, Liu Y, Li J, Wang Y, Ji P, Shi Q, et al. Ginsenoside Rg1 alleviates chronic inflammation-induced neuronal ferroptosis and cognitive impairments via regulation of AIM2 - Nrf2 signaling pathway*.* J Ethnopharmacol 2024; 330: 118205.

21.	Liu Z, Cecarini V, Cuccioloni M, Bonfili L, Gong C, Angeletti M, et al. Ginsenosides Rg1 and Rg2 Activate Autophagy and Attenuate Oxidative Stress in Neuroblastoma Cells Overexpressing Abeta(1-42)*.* Antioxidants (Basel) 2024; 13(3): 310.

22.	Gong D, Xia F, Wang F, Tong X, Yang Q, Shen K, et al. Does ginsenoside Rg1 promote intervertebral disc repair? An experimental study insights into ferroptosis mechanism*.* J Transl Med 2025; 23(1): 1231.

23.	Zhang B, Zhang X, Zhang C, Shen Q, Sun G, and Sun X. Notoginsenoside R1 Protects db/db Mice against Diabetic Nephropathy via Upregulation of Nrf2-Mediated HO-1 Expression*.* Molecules 2019; 24(2): 247.

24.	Zhang Y, Ma J, Liu S, Chen C, Li Q, Qin M, et al. Ginsenoside F1 attenuates pirarubicin-induced cardiotoxicity by modulating Nrf2 and AKT/Bcl-2 signaling pathways*.* J Ginseng Res 2023; 47(1): 106-16.

25.	Du F, Huang H, Cao Y, Ran Y, Wu Q, and Chen B. Notoginsenoside R1 Protects Against High Glucose-Induced Cell Injury Through AMPK/Nrf2 and Downstream HO-1 Signaling*.* Front Cell Dev Biol 2021; 9: 791643.

26.	Wang K, Wu D, Ren X, Yu Q, Liu R, Huang S, et al. Notoginsenoside R1 alleviates TEGDMA-induced mitochondrial apoptosis in preodontoblasts through activation of Akt/Nrf2 pathway-dependent mitophagy*.* Toxicol Appl Pharmacol 2021; 417: 115482.

27.	Wang J, He X, and Lv S. Notoginsenoside-R1 ameliorates palmitic acid-induced insulin resistance and oxidative stress in HUVEC via Nrf2/ARE pathway*.* Food Sci Nutr 2023; 11(12): 7791-802.

28.	He SY, Liu RP, Wang CR, Wang XQ, Wang J, Xu YN, et al. Improving the developmental competences of porcine parthenogenetic embryos by Notoginsenoside R1-induced enhancement of mitochondrial activity and alleviation of proapoptotic events*.* Reprod Domest Anim 2023; 58(11): 1583-94.

29.	He SY, Liu W, Huang CM, Huang HM, Cao QL, Li YX, et al. Enhancing antioxidant levels and mitochondrial function in porcine oocyte maturation and embryonic development through notoginsenoside R1 supplementation*.* Reprod Domest Anim 2024; 59(6): e14631.

30.	Xu Y, Wang P, Hu T, Ning K, and Bao Y. Notoginsenoside R1 Attenuates H/R Injury in H9c2 Cells by Maintaining Mitochondrial Homeostasis*.* Curr Issues Mol Biol 2025; 47(1): 44.

31.	Feng SL, Luo HB, Cai L, Zhang J, Wang D, Chen YJ, et al. Ginsenoside Rg5 overcomes chemotherapeutic multidrug resistance mediated by ABCB1 transporter: in vitro and in vivo study*.* J Ginseng Res 2020; 44(2): 247-57.

32.	Kim B, Kim YS, Li W, Kwon EB, Chung HS, Go Y, et al. Ginsenoside Rg5, a potent agonist of Nrf2, inhibits HSV-1 infection-induced neuroinflammation by inhibiting oxidative stress and NF-kappaB activation*.* J Ginseng Res 2024; 48(4): 384-94.

33.	Shan GY, Shi YP, Zhang YX, Wan H, Gao ZC, and Li HJ. Ginsenoside Rg5 ameliorates lipopolysaccharide (LPS)-induced acute liver injury via interfering Autophagy/Nrf2/Ferroptosis signal axis*.* Phytomedicine 2025; 144: 156941.

34.	Zhu A, Duan Z, Chen Y, Zhu C, and Fan D. Ginsenoside Rh4 delays skeletal muscle aging through SIRT1 pathway*.* Phytomedicine 2023; 118: 154906.

35.	Hu JN, Xu XY, Jiang S, Liu Y, Liu Z, Wang YP, et al. Protective effect of ginsenoside Rk1, a major rare saponin from black ginseng, on cisplatin-induced nephrotoxicity in HEK-293 cells*.* Kaohsiung J Med Sci 2020; 36(9): 732-40.

36.	Xiong J, Yang J, Yan K, and Guo J. Ginsenoside Rk1 protects human melanocytes from H2O2induced oxidative injury via regulation of the PI3K/AKT/Nrf2/HO1 pathway*.* Mol Med Rep 2021; 24(5): 821.

37.	Ren Y, Ye D, Ding Y, and Wei N. Ginsenoside Rk1 prevents 1-methyl-4-phenyl-1,2,3,6-tetrahydropyridine-induced Parkinson's disease via activating silence information regulator 3-mediated Nrf2/HO-1 signaling pathway*.* Hum Exp Toxicol 2023; 42: 9603271231220610.

38.	She L, Sun J, Xiong L, Li A, Li L, Wu H, et al. Ginsenoside RK1 improves cognitive impairments and pathological changes in Alzheimer's disease via stimulation of the AMPK/Nrf2 signaling pathway*.* Phytomedicine 2024; 122: 155168.

39.	She L, Xiong L, Li L, Zhang J, Sun J, Wu H, et al. Ginsenoside Rk3 ameliorates Abeta-induced neurotoxicity in APP/PS1 model mice via AMPK signaling pathway*.* Biomed Pharmacother 2023; 158: 114192.

40.	Qi Z, Wang Z, Zhou B, Fu S, Hong T, Li P, et al. A new ocotillol-type ginsenoside from stems and leaves of Panax quinquefolium L. and its anti-oxidative effect on hydrogen peroxide exposed A549 cells*.* Nat Prod Res 2020; 34(17): 2474-81.

41.	Liu XY, Xiao YK, Hwang E, Haeng JJ, and Yi TH. Antiphotoaging and Antimelanogenesis Properties of Ginsenoside C-Y, a Ginsenoside Rb2 Metabolite from American Ginseng PDD-ginsenoside*.* Photochem Photobiol 2019; 95(6): 1412-23.

42.	Sun G, Wang J, Xu X, Zhai L, Li Z, Liu J, et al. Panax ginseng Meyer cv. Silvatica phenolic acids protect DNA from oxidative damage by activating Nrf2 to protect HFF-1 cells from UVA-induced photoaging*.* J Ethnopharmacol 2023; 302(Pt A): 115883.

43.	Yang S, Li W, Bai X, Di Nunzio G, Fan L, Zhao Y, et al. Ginseng-derived nanoparticles alleviate inflammatory bowel disease via the TLR4/MAPK and p62/Nrf2/Keap1 pathways*.* J Nanobiotechnology 2024; 22(1): 48.

44.	Li W, Yang S, Zhao Y, Di Nunzio G, Ren L, Fan L, et al. Ginseng-derived nanoparticles alleviate alcohol-induced liver injury by activating the Nrf2/HO-1 signalling pathway and inhibiting the NF-kappaB signalling pathway in vitro and in vivo*.* Phytomedicine 2024; 127: 155428.

45.	Choi EJ, Kim H, Hong KB, Suh HJ, and Ahn Y. Hangover-Relieving Effect of Ginseng Berry Kombucha Fermented by Saccharomyces cerevisiae and Gluconobacter oxydans in Ethanol-Treated Cells and Mice Model*.* Antioxidants (Basel) 2023; 12(3): 774.

46.	Yan X, Chen X, Fu C, Jing C, Zhao D, and Sun L. Ginseng oligosaccharides protect neurons from glutamate-induced oxidative damage through the Nrf2/HO-1 signaling pathway*.* Food Funct 2022; 13(16): 8605-15.

47.	Yang S, Li F, Lu S, Ren L, Bian S, Liu M, et al. Ginseng root extract attenuates inflammation by inhibiting the MAPK/NF-kappaB signaling pathway and activating autophagy and p62-Nrf2-Keap1 signaling in vitro and in vivo*.* J Ethnopharmacol 2022; 283: 114739.

48.	Qi Z, Li Z, Guan X, Wang C, Wang F, Li P, et al. Four Novel Dammarane-Type Triterpenoids from Pearl Knots of Panax ginseng Meyer cv. Silvatica*.* Molecules 2019; 24(6): 1159.

49.	Liu XY, Li H, Hwang E, Park B, Xiao YK, Liu S, et al. Chemical Distance Measurement and System Pharmacology Approach Uncover the Novel Protective Effects of Biotransformed Ginsenoside C-Mc against UVB-Irradiated Photoaging*.* Oxid Med Cell Longev 2022; 2022: 4691576.

50.	Jang M, Choi JH, Chang Y, Lee SJ, Nah SY, and Cho IH. Gintonin, a ginseng-derived ingredient, as a novel therapeutic strategy for Huntington's disease: Activation of the Nrf2 pathway through lysophosphatidic acid receptors*.* Brain Behav Immun 2019; 80: 146-62.

51.	Jang H, Kim H, Oh SH, Son Y, Lee R, Nah SY, et al. Gintonin enhances epithelial barrier function by activating NRF2 pathway in radiation-induced intestinal injury*.* J Ginseng Res 2025; 49(3): 248-59.

52.	Ha Y, Jo HS, Kwon TW, Jeon SH, Moon SK, Jung JH, et al. Korean black ginseng extract alleviates Alzheimer's disease-related cognitive impairment by activating the Nrf2/HO-1 pathway and suppressing the p38 MAPK/NF-kappaB/STAT3 pathways and NLRP3 inflammasome via TLR2 and TLR4 modulation*.* J Ginseng Res 2025; 49(3): 294-305.

53.	Kang B, Kim CY, Hwang J, Sun S, Yang H, Suh HJ, et al. Red ginseng extract regulates differentiation of monocytes to macrophage and inflammatory signalings in human monocytes*.* Food Sci Biotechnol 2019; 28(6): 1819-28.

54.	Jo YH, Lee H, Oh MH, Lee GH, Lee YJ, Lee JS, et al. Antioxidant and hepatoprotective effects of Korean ginseng extract GS-KG9 in a D-galactosamine-induced liver damage animal model*.* Nutr Res Pract 2020; 14(4): 334-51.

55.	Zhu DC, Wang YH, Lin JH, Miao ZM, Xu JJ, and Wu YS. Maltol inhibits the progression of osteoarthritis via the nuclear factor-erythroid 2-related factor-2/heme oxygenase-1 signal pathway in vitro and in vivo*.* Food Funct 2021; 12(3): 1327-37.

56.	Liang Y, Wang Z, Huo D, Hu JN, Song L, Ma X, et al. Nanoplastic-Induced Liver Damage Was Alleviated by Maltol via Enhancing Autophagic Flow: An In Vivo and In Vitro Study*.* J Agric Food Chem 2024; 72(29): 16250-62.

57.	He X, Wu M, Chen L, Liu M, Hu X, Meng Y, et al. APMCG-1 attenuates ischemic stroke injury by reducing oxidative stress and apoptosis and promoting angiogenesis via activating PI3K/AKT pathway*.* Biomed Pharmacother 2024; 180: 117506.

58.	Wu Y, Zhang J, Tian Y, Chi Shing Cho W, Xu HX, Lin ZX, et al. 20(S)-Ginsenoside Rh2 overcomes gemcitabine resistance in pancreatic cancer by inhibiting LAMC2-Modulated ABC transporters*.* J Adv Res 2025; 73: 743-60.

59.	Kim EN, Kim TY, Park EK, Kim JY, and Jeong GS. Panax ginseng Fruit Has Anti-Inflammatory Effect and Induces Osteogenic Differentiation by Regulating Nrf2/HO-1 Signaling Pathway in In Vitro and In Vivo Models of Periodontitis*.* Antioxidants (Basel) 2020; 9(12): 1221.

60.	ATAG DEF, Figueiredo Pinho C, de Aquino AM, Fernandes AAH, Fantin Domeniconi R, Justulin LA, et al. Panax ginseng methabolit (GIM-1) prevents oxidative stress and apoptosis in human Sertoli cells exposed to Monobutyl-phthalate (MBP)*.* Reprod Toxicol 2019; 86: 68-75.

61.	Ji YJ, Kim HD, Lee ES, Jang GY, and Seong HA. Heat Treatment Enhances the Neuroprotective Effects of Crude Ginseng Saponin by Increasing Minor Ginsenosides*.* Int J Mol Sci 2023; 24(8): 7223.

62.	Dai C, Yusuf A, Sun H, Shu G, and Deng X. A characterized saponin extract of Panax japonicus suppresses hepatocyte EMT and HSC activation in vitro and CCl(4)-provoked liver fibrosis in mice: Roles of its modulatory effects on the Akt/GSK3beta/Nrf2 cascade*.* Phytomedicine 2021; 93: 153746.

63.	Qiu L, Feng R, Wu QS, Wan JB, and Zhang QW. Total saponins from Panax japonicus attenuate acute alcoholic liver oxidative stress and hepatosteatosis by p62-related Nrf2 pathway and AMPK-ACC/PPARalpha axis in vivo and in vitro*.* J Ethnopharmacol 2023; 317: 116785.

64.	Hu S, Liu T, Wu Y, Yang W, Hu S, Sun Z, et al. Panax notoginseng saponins suppress lipopolysaccharide-induced barrier disruption and monocyte adhesion on bEnd.3 cells via the opposite modulation of Nrf2 antioxidant and NF-kappaB inflammatory pathways*.* Phytother Res 2019; 33(12): 3163-76.

65.	Zhang X, Li X, Xiong P, Yi C, and Chen X. [Effects of Panax notoginseng saponins on liver graft rejection in rats and the mechanisms]*.* Nan Fang Yi Ke Da Xue Xue Bao 2019; 39(4): 394-400.

66.	Fei B, Liu Z, Xie L, Lv L, Zhu W, Liu J, et al. Panax notoginseng Saponins protect auditory cells against cisplatin‑induced ototoxicity by inducing the AKT/Nrf2 signaling‑mediated redox pathway*.* Mol Med Rep 2020; 22(4): 3533-40.

67.	Lu X, Liu X, Liang E, Yang R, Liu Y, Liu X, et al. Panax quinquefolius saponin inhibits vascular smooth muscle cell calcification via activation of nuclear factor-erythroid 2-related factor 2*.* BMC Complement Med Ther 2023; 23(1): 129.

68.	Li J, Lu K, Sun F, Tan S, Zhang X, Sheng W, et al. Panaxydol attenuates ferroptosis against LPS-induced acute lung injury in mice by Keap1-Nrf2/HO-1 pathway*.* J Transl Med 2021; 19(1): 96.

69.	Mi A, Hu Q, Liu Y, Zhao Y, Shen F, Lan J, et al. Hepatoprotective efficacy and interventional mechanism of the panaxadiol saponin component in high-fat diet-induced NAFLD mice*.* Food Funct 2024; 15(2): 794-808.

70.	Yang Z, Gao Y, Li D, Zhao L, and Du Y. Panaxadiol Saponin Alleviates Lps-Induced Cardiomyopathy Similar to Dexamethasone Via Improving Mitochondrial Quality Control*.* Shock 2025; 63(2): 282-91.

71.	Jeong SY, Kim JE, Song GY, and Bae JS. [Formula: see text], a Rare Protopanaxatriol-Type Ginsenoside Fraction from Black Ginseng, Suppresses Inflammatory Gene iNOS via the Iinhibition of p-STAT-1 and NF-[Formula: see text]B*.* Am J Chin Med 2020; 48(5): 1091-102.

72.	Li W, Wang Y, Zhang Y, Fan Y, Liu J, Zhu K, et al. Lizhong decoction ameliorates ulcerative colitis by inhibiting ferroptosis of enterocytes via the Nrf2/SLC7A11/GPX4 pathway*.* J Ethnopharmacol 2024; 326: 117966.

73.	Zhou P, Gao G, Zhao CC, Li JY, Peng JF, Wang SS, et al. In vivo and in vitro protective effects of shengmai injection against doxorubicin-induced cardiotoxicity*.* Pharm Biol 2022; 60(1): 638-51.

74.	Jin W, Zhu X, Yao F, Xu X, Chen X, Luo Z, et al. Cytoprotective effect of Fufang Lurong Jiangu capsule against hydrogen peroxide-induced oxidative stress in bone marrow stromal cell-derived osteoblasts through the Nrf2/HO-1 signaling pathway*.* Biomed Pharmacother 2020; 121: 109676.

75.	Lu J, Tang X, Zhang Y, Chu H, Jing C, Wang Y, et al. Exploring the molecular mechanism of Yinao Fujian formula on ischemic stroke based on network pharmacology and experimental verification*.* Heliyon 2024; 10(1): e23742.

76.	Zhang H, Li L, Hao M, Chen K, Lu Y, Qi J, et al. Yixin-Fumai granules improve sick sinus syndrome in aging mice through Nrf-2/HO-1 pathway: A new target for sick sinus syndrome*.* J Ethnopharmacol 2021; 277: 114254.

77.	Chen S, Li X, Wang Y, Mu P, Chen C, Huang P, et al. Ginsenoside Rb1 attenuates intestinal ischemia/reperfusion‑induced inflammation and oxidative stress via activation of the PI3K/Akt/Nrf2 signaling pathway*.* Mol Med Rep 2019; 19(5): 3633-41.

78.	Jiang N, Zhang Y, Yao C, Huang H, Wang Q, Huang S, et al. Ginsenosides Rb1 Attenuates Chronic Social Defeat Stress-Induced Depressive Behavior via Regulation of SIRT1-NLRP3/Nrf2 Pathways*.* Front Nutr 2022; 9: 868833.

79.	Shi L, Fu W, Xu H, Li S, Yang X, Yang W, et al. Ginsenoside Rc attenuates myocardial ischaemic injury through antioxidative and anti-inflammatory effects*.* Pharm Biol 2022; 60(1): 1038-46.

80.	Pan Z, Guo J, Tang K, Chen Y, Gong X, Chen Y, et al. Ginsenoside Rc Modulates SIRT6-NRF2 Interaction to Alleviate Alcoholic Liver Disease*.* J Agric Food Chem 2022; 70(44): 14220-34.

81.	Liu W, Zhou X, Xiao L, Huang X, Chang D, Zhong X, et al. The gut microbiota-mediated ferroptosis pathway: a key mechanism of ginsenoside Rd against metabolism-associated fatty liver disease*.* Chin Med 2025; 20(1): 83.

82.	Chang SN, Park JG, and Kang SC. Therapeutic propensity of ginsenosides Rg1 and Rg3 in rhabdomyolysis-induced acute kidney injury and renohepatic crosstalk in rats*.* Int Immunopharmacol 2023; 115: 109602.

83.	He Y, Wang Y, Duan H, Huang D, Jia N, Shen Z, et al. Pharmacological targeting of ferroptosis in hypoxia-induced pulmonary edema: therapeutic potential of ginsenoside Rg3 through activation of the PI3K/AKT pathway*.* Front Pharmacol 2025; 16: 1644436.

84.	Yang Q, Lin J, Zhang H, Liu Y, Kan M, Xiu Z, et al. Ginsenoside Compound K Regulates Amyloid beta via the Nrf2/Keap1 Signaling Pathway in Mice with Scopolamine Hydrobromide-Induced Memory Impairments*.* J Mol Neurosci 2019; 67(1): 62-71.

85.	Zhang J, Liu Y, An C, Liu C, Ma S, Zhang Q, et al. Protective Effect of Ginsenoside CK against Autoimmune Hepatitis Induced by Concanavalin A*.* Foods 2023; 12(24): 4379.

86.	Wang H, Lv J, Jiang N, Huang H, Wang Q, and Liu X. Ginsenoside Re protects against chronic restraint stress-induced cognitive deficits through regulation of NLRP3 and Nrf2 pathways in mice*.* Phytother Res 2021; 35(5): 2523-35.

87.	Nguyen BT, Shin EJ, Jeong JH, Sharma N, Nah SY, Ko SK, et al. Ginsenoside Re attenuates memory impairments in aged Klotho deficient mice via interactive modulations of angiotensin II AT1 receptor, Nrf2 and GPx-1 gene*.* Free Radic Biol Med 2022; 189: 2-19.

88.	Shin EJ, Jeong JH, Nguyen BT, Sharma N, Tran CNK, Nah SY, et al. Ginsenoside Re attenuates 8-OH-DPAT-induced serotonergic behaviors in mice via interactive modulation between PKCdelta gene and Nrf2*.* Drug Chem Toxicol 2022; 46(2): 281-96.

89.	Qin Q, Lin N, Huang H, Zhang X, Cao X, Wang Y, et al. Ginsenoside Rg1 ameliorates cardiac oxidative stress and inflammation in streptozotocin-induced diabetic rats*.* Diabetes Metab Syndr Obes 2019; 12: 1091-103.

90.	Gao Y, Li J, Chu S, Zhang Z, Chen N, Li L, et al. Ginsenoside Rg1 protects mice against streptozotocin-induced type 1 diabetic by modulating the NLRP3 and Keap1/Nrf2/HO-1 pathways*.* Eur J Pharmacol 2020; 866: 172801.

91.	Wang Z, Du K, Hou J, Xiao H, Hu L, Chen X, et al. Rg1 alleviates oxidative stress and spermatogonium apoptosis in D-gal-induced testicular toxicity by activating Akt*.* Redox Rep 2023; 28(1): 2206197.

92.	Gao Q, Li G, Zu Y, Xu Y, Wang C, Xiang D, et al. Ginsenoside Rg1 alleviates ANIT-induced cholestatic liver injury by inhibiting hepatic inflammation and oxidative stress via SIRT1 activation*.* J Ethnopharmacol 2024; 319(Pt 1): 117089.

93.	Zhou Y, Zhang Y, Wang L, Liu Y, Wang Z, and Guo L. Ginsenoside Rg1 regulating inflammatory response and bone-remodeling through Keap1/Nrf2 signaling pathway in rats with periodontitis*.* Sci Rep 2025; 15(1): 7478.

94.	Zhang Y, Zhao Y, Ran Y, Guo J, Cui H, and Liu S. Notoginsenoside R1 attenuates sevoflurane-induced neurotoxicity*.* Transl Neurosci 2020; 11(1): 215-26.

95.	Wang Y, Yin Y, Liu Y, Pei C, Shen Z, Zhao S, et al. Notoginsenoside R1 treatment facilitated Nrf2 nuclear translocation to suppress ferroptosis via Keap1/Nrf2 signaling pathway to alleviated high-altitude myocardial injury*.* Biomed Pharmacother 2024; 175: 116793.

96.	Nan B, Ren G, Ge S, Liu Y, Zhou L, Cui L, et al. Components Changes in Fresh Ginseng Pulp Treated With Commercial Sterilization and Its Potential Therapeutic in CTX-Induced Liver Injury via Apoptosis and Nrf2-MAPKs/NF-kappaB Pathways*.* Food Sci Nutr 2025; 13(11): e71190.

97.	Chen LX, Qi YL, Qi Z, Gao K, Gong RZ, Shao ZJ, et al. A Comparative Study on the Effects of Different Parts of Panax ginseng on the Immune Activity of Cyclophosphamide-Induced Immunosuppressed Mice*.* Molecules 2019; 24(6): 1096.

98.	Salama A, Mansour D, and Hegazy R. The cardio and renoprotective role of ginseng against epinephrine-induced myocardial infarction in rats: Involvement of angiotensin II type 1 receptor/protein kinase C*.* Toxicol Rep 2021; 8: 908-19.

99.	Pei H, Liu S, Zeng J, Liu J, Wu H, Chen W, et al. Ros-mediated mitochondrial oxidative stress is involved in the ameliorating effect of ginsenoside GSLS on chlorpyrifos induced hepatotoxicity in mice*.* Aging (Albany NY) 2022; 15(3): 675.

100.	Nam SM, Choi SH, Cho HJ, Seo JS, Choi M, Nahm SS, et al. Ginseng Gintonin Attenuates Lead-Induced Rat Cerebellar Impairments during Gestation and Lactation*.* Biomolecules 2020; 10(3): 385.

101.	Choi JH, Oh J, Lee MJ, Ko SG, Nah SY, and Cho IH. Gintonin mitigates experimental autoimmune encephalomyelitis by stabilization of Nrf2 signaling via stimulation of lysophosphatidic acid receptors*.* Brain Behav Immun 2021; 93: 384-98.

102.	Choi JH, Kwon TW, Jo HS, Ha Y, and Cho IH. Gintonin, a Panax ginseng-derived LPA receptor ligand, attenuates kainic acid-induced seizures and neuronal cell death in the hippocampus via anti-inflammatory and anti-oxidant activities*.* J Ginseng Res 2023; 47(3): 390-9.

103.	Tang P, Ren G, Zou H, Liu S, Zhang J, Ai Z, et al. Ameliorative effect of total ginsenosides from heat-treated fresh ginseng against cyclophosphamide-induced liver injury in mice*.* Curr Res Food Sci 2024; 8: 100734.

104.	Zhang L, Qi X, Lu XT, Cui CB, and Gao XF. Study on hypoglycemic effects of irradiated ginseng adventitious roots*.* Food Chem X 2022; 13: 100234.

105.	Chou TW, Huang HS, Panyod S, Huang YJ, and Sheen LY. Korean red ginseng water extract produces antidepressant-like effects through involving monoamines and brain-derived neurotrophic factor in rats*.* J Ginseng Res 2023; 47(4): 552-60.

106.	Sha JY, Zhou YD, Yang JY, Leng J, Li JH, Hu JN, et al. Maltol (3-Hydroxy-2-methyl-4-pyrone) Slows d-Galactose-Induced Brain Aging Process by Damping the Nrf2/HO-1-Mediated Oxidative Stress in Mice*.* J Agric Food Chem 2019; 67(37): 10342-51.

107.	Nguyen BT, Shin EJ, Jeong JH, Sharma N, Tran NKC, Nguyen YND, et al. Mountain-cultivated ginseng protects against cognitive impairments in aged GPx-1 knockout mice via activation of Nrf2/ChAT/ERK signaling pathway*.* J Ginseng Res 2023; 47(4): 561-71.

108.	Aziz RLA, Abdel-Wahab A, Abdel-Razik AH, Kamel S, Farghali AA, Saleh R, et al. Physiological roles of propolis and red ginseng nanoplatforms in alleviating dexamethasone-induced male reproductive challenges in a rat model*.* Mol Biol Rep 2024; 51(1): 72.

109.	Ghamry HI, Aboushouk AA, Soliman MM, Albogami SM, Tohamy HG, Okle OSE, et al. Ginseng((R)) Alleviates Malathion-Induced Hepatorenal Injury through Modulation of the Biochemical, Antioxidant, Anti-Apoptotic, and Anti-Inflammatory Markers in Male Rats*.* Life (Basel) 2022; 12(5): 771.

110.	Qin Y, Zhang RY, Zhang Y, Zhao YQ, Hao HF, and Wang JP. Network pharmacology and in vivo study: Unraveling the therapeutic mechanisms of Panax ginseng in potentially treating ulcerative colitis*.* World J Gastroenterol 2025; 31(9): 100271.

111.	Liu P, Yang T, Zhang J, Su B, Shi Q, Song Y, et al. Panax ginseng alleviates diabetic peripheral neuropathy by suppressing mitochondrial dysfunction and oxidative stress via modulation of the RAGE, NF-kappaB, and Nrf2 pathways*.* J Ginseng Res 2025; 49(6): 734-45.

112.	Gao Y, Yuan D, Gai L, Wu X, Shi Y, He Y, et al. Saponins from Panax japonicus ameliorate age-related renal fibrosis by inhibition of inflammation mediated by NF-kappaB and TGF-beta1/Smad signaling and suppression of oxidative stress via activation of Nrf2-ARE signaling*.* J Ginseng Res 2021; 45(3): 408-19.

113.	Wang C, Zheng L, Liu S, Guo X, Qu Y, Gao M, et al. A novel acidic polysaccharide from the residue of Panax notoginseng and its hepatoprotective effect on alcoholic liver damage in mice*.* Int J Biol Macromol 2020; 149: 1084-97.

114.	Mi W, Yu M, Yin S, Ji Y, Shi T, and Li N. Analysis of the Renal Protection and Antioxidative Stress Effects of Panax notoginseng Saponins in Diabetic Nephropathy Mice*.* J Immunol Res 2022; 2022: 3610935.

115.	Kang Z, Xiao Q, Wang L, Xiao L, and Tang B. The combination of astragaloside IV and Panax notoginseng saponins attenuates cerebral ischaemia-reperfusion injury in rats through ferroptosis and inflammation inhibition via activating Nrf2*.* J Pharm Pharmacol 2023; 75(5): 666-76.

116.	Wu J, Chen X, Lin X, Li Z, Cao Z, Huang W, et al. Protective Effects of Panax notoginseng Saponins on Cerebral Ischemia/Reperfusion Injury: Insights Into SIRT1/NRF2/HO-1 Pathway Activation*.* Physiol Res 2025; 74(2): 313-26.

117.	Guo Y, Hu M, Ma J, Chinnathambi A, Alharbi SA, Shair OHM, et al. Protective effect of panaxydol against repeated administration of aristolochic acid on renal function and lipid peroxidation products via activating Keap1-Nrf2/ARE pathway in rat kidney*.* J Biochem Mol Toxicol 2021; 35(1): e22619.

118.	Lian Y, Zhu M, Yang B, Wang X, Zeng J, Yang Y, et al. Characterization of a novel polysaccharide from red ginseng and its ameliorative effect on oxidative stress injury in myocardial ischemia*.* Chin Med 2022; 17(1): 111.

119.	Shang Z, Fan M, Zhang J, Wang Z, Jiang S, and Li W. Red Ginseng Improves D-galactose-Induced Premature Ovarian Failure in Mice Based on Network Pharmacology*.* Int J Mol Sci 2023; 24(9): 8210.

120.	Yoshikawa N, Hirata N, Kurone Y, and Shimoeda S. Red ginseng prevents doxorubicin-induced cardiomyopathy by inhibiting cell death via activating the Nrf2 pathway*.* Cardiooncology 2024; 10(1): 39.

121.	Mei SL, Xia ZY, Qiu Z, Jia YF, Zhou JJ, and Zhou B. Shenmai Injection Attenuates Myocardial Ischemia/Reperfusion Injury by Targeting Nrf2/GPX4 Signalling-Mediated Ferroptosis*.* Chin J Integr Med 2022; 28(11): 983-91.

122.	Rahimi G, Heydari S, Rahimi B, Abedpoor N, Niktab I, Safaeinejad Z, et al. A combination of herbal compound (SPTC) along with exercise or metformin more efficiently alleviated diabetic complications through down-regulation of stress oxidative pathway upon activating Nrf2-Keap1 axis in AGE rich diet-induced type 2 diabetic mice*.* Nutr Metab (Lond) 2021; 18(1): 14.

123.	Hu Y, Sun J, Wang T, Wang H, Zhao C, Wang W, et al. Compound Danshen Dripping Pill inhibits high altitude-induced hypoxic damage by suppressing oxidative stress and inflammatory responses*.* Pharm Biol 2021; 59(1): 1585-93.

124.	Qiu M, Xiao F, Wang T, Piao S, Zhao W, Shao S, et al. Protective effect of Hedansanqi Tiaozhi Tang against non-alcoholic fatty liver disease in vitro and in vivo through activating Nrf2/HO-1 antioxidant signaling pathway*.* Phytomedicine 2020; 67: 153140.

125.	Zhang B, Zhang CY, Zhang XL, Sun GB, and Sun XB. Guan Xin Dan Shen formulation protects db/db mice against diabetic cardiomyopathy via activation of Nrf2 signaling*.* Mol Med Rep 2021; 24(1): 531.

126.	Luo D, Yang L, Pang H, Zhao Y, Li K, Rong X, et al. Tianhuang formula reduces the oxidative stress response of NAFLD by regulating the gut microbiome in mice*.* Front Microbiol 2022; 13: 984019.
